# Supplementary material for: Inhibition of vascular calcification by inositol phosphates derivatized with ethylene glycol oligomers
Source: Nat Commun. 2020 Feb 5;11:721. doi: 10.1038/s41467-019-14091-4 (PMC7002685; doi:10.1038/s41467-019-14091-4)
Supplement: Supplementary file 1 — Supplementary information [file 41467_2019_14091_MOESM1_ESM.pdf]

Supplementary information

Inhibition of vascular calcification by inositol phosphates derivatized with ethylene glycol oligomers

Antonia E. Schantl[1,8], Anja Verhulst[2,8], Ellen Neven[2], Geert Behets[2], Patrick C. D'Haese[2], Marc Maillard[3], David Mordasini[3], Olivier Phan[3], Michel Burnier[3], Dany Spaggiari[4], Laurent A. Decosterd[4], Mark G. MacAskill[5], Carlos J. Alcaide-Corral[5], Adriana A.S. Tavares[5], David E. Newby[5], Victoria C. Beindl[1], Roberto Maj[6], Anne Labarre[7], Chrismita Hegde[7], Bastien Castagner[7], Mattias E. Ivarsson[6], and Jean-Christophe Leroux[1 *]*

[1] Institute of Pharmaceutical Sciences, Department of Chemistry and Applied Biosciences, ETH Zurich, Zurich, Switzerland

[2] Laboratory of Pathophysiology, University of Antwerp, Antwerp, Belgium

[3] Service of Nephrology and Hypertension, Lausanne University Hospital, Lausanne, Switzerland

[4] Division of Clinical Pharmacology, Lausanne University Hospital, Lausanne, Switzerland

[5] University-BHF Centre for Cardiovascular Science, Queen's Medical Research Institute, University of Edinburgh, Edinburgh, UK

[6] Inositec Inc., Zurich, Switzerland

[7] Department of Pharmacology & Therapeutics, McGill University, Montreal, Canada

[8] These authors contributed equally: Antonia E. Schantl, Anja Verhulst

[*] These authors jointly directed this work: Mattias E. Ivarsson, Jean-Christophe Leroux

e-mail: mattias@inositec.com, jleroux@ethz.ch

INDEX

1. **Supplementary Figures** 2
2. **Supplementary Tables** 22
3. **Supplemental Methods** 32
4. **Supplemental References** 65
5. **Appendix NMR Spectra** 40
6. **Appendix ESI-MS Spectra** 51
7. **Appendix HPLC MS/MS Spectrum** 64

1. Supplementary Figures

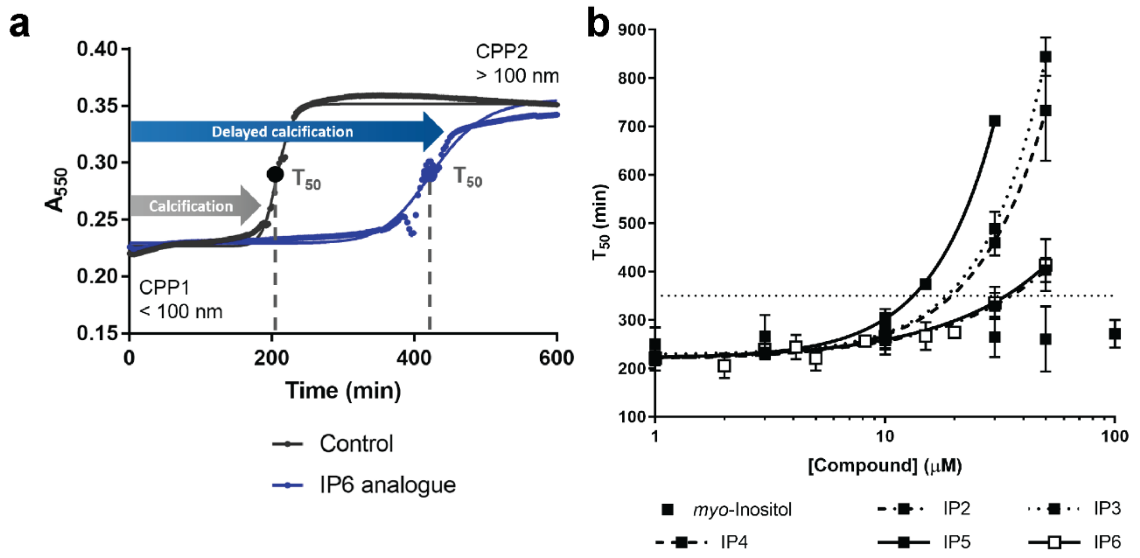

Supplementary Fig. 1: a, Schematic illustration of the assay principle. Human serum was spiked with 6 mM phosphate and 10 mM calcium. CPP structural rearrangement from CPP1 to CPP2, indicated by shift in absorbance (550 nm), was monitored over time at 37 °C. Addition of calcification inhibitors delays CPP transition. **b**, Delay in serum calcification propensity by lower phosphorylated inositol phosphate control compounds (n = 4 for *myo*-inositol, n = 3 for inositol phosphates). All data points represent the T_{50} (min) as mean ± s.d. from n independent experiments and the dotted horizontal line represents the c350, (i.e., the concentration necessary to delay T_{50} to 350 min), which was used to rank the inhibitor activity of all tested compounds.

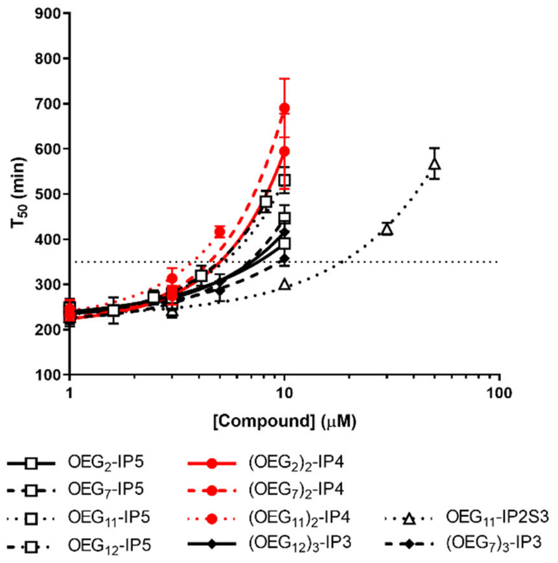

Supplementary Fig. 2. Delay in serum calcification propensity by functionalized inositol phosphates (n = 5 for OEG_{12}-IP5 and OEG_{11}-IP2S3, n = 6 for $(OEG_2)_2$-IP4, n = 26 for IP6, and n = 3 for all others). All data points represent the T_{50} (min) as mean ± s.d. from n independent experiments and the dotted horizontal line represents the c350, (i.e., the concentration necessary to delay T_{50} to 350 min), which was used to rank the inhibitor activity of all tested compounds.

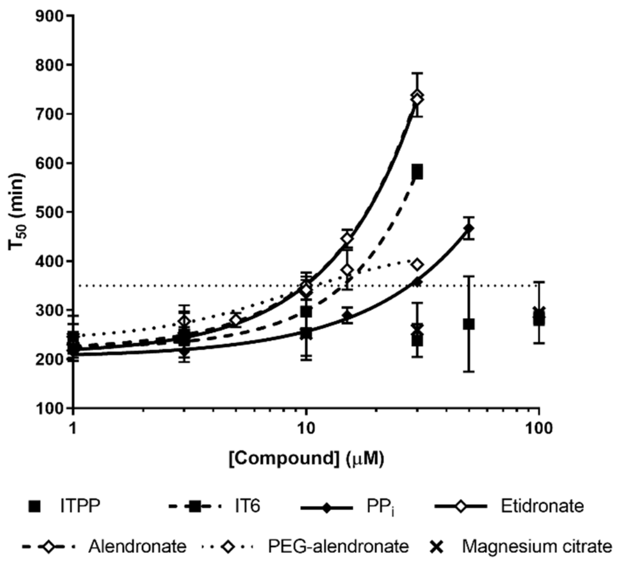

Supplementary Fig. 3 Delay in serum calcification propensity by control compounds (n = 3 for magnesium citrate, PEG-alendronate, IT6 and PP_i, n = 4 for etidronate and ITPP, and n = 6 for alendronate). All data points represent the T_{50} (min) as mean ± s.d. from n independent experiments and the dotted horizontal line represents the c350, (i.e., the concentration necessary to delay T_{50} to 350 min), which was used to rank the inhibitor activity of all tested compounds.

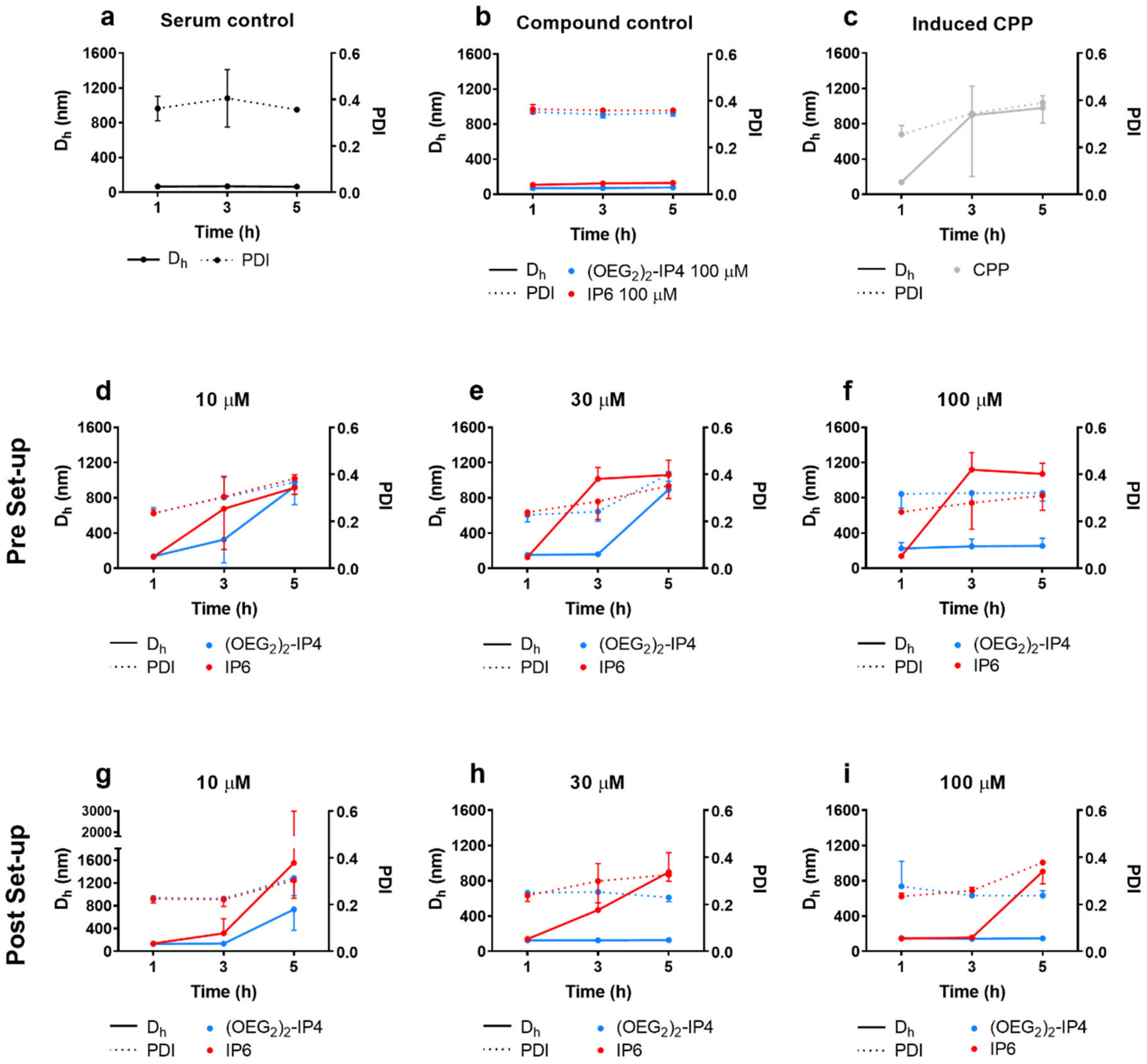

Supplementary Fig. 4: CPP hydrodynamic diameter (D_h) maturation and polydispersity index (PDI) analyzed by dynamic light scattering. **a**, Serum background particle size did not change over time and were polydisperse (D_h = 66 ± 5, 68 ± 2 and 64 ± 1 nm after 1, 3 and 5 h, respectively; PDI range 0.356 – 0.405). **b,** Compounds at 100 µM did not alter D_h of serum background particles. **c,** CPP formation was initiated by addition of 6 mM phosphate and 10 mM calcium. CPP1 present at 1 h (D_h = 137 ± 14 nm; PDI 0.217 – 0.295) and CPP2 present at 5 h (D_h = 979 ± 170 nm; PDI 0.355 – 0.409). **d,-f**, Human serum was mixed with increasing concentrations of compounds and subsequently spiked with calcium and phosphate to initiate CPP maturation (pre-setup). **g,-i**, Calcium and phosphate was added to human serum to initiate CPP maturation and subsequently, without further incubation time, increasing concentrations of test compounds were added (post-setup). Interestingly, CPP crystal growth

retardation was stronger when $(OEG_2)_2$-IP4 was added after formation of CPP1, compared to when the compound was added to serum before CPPs were formed. Data are expressed as mean ± s.d. (n = 3).

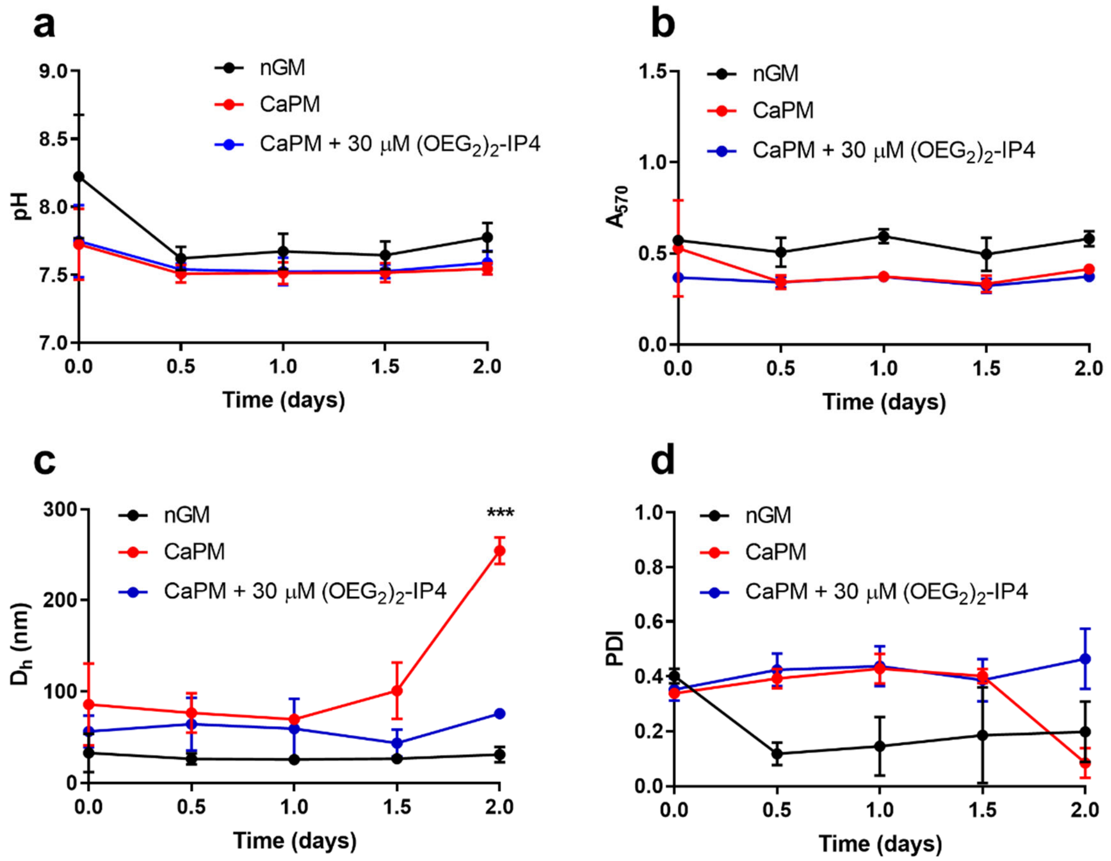

Supplementary Fig. 5: Measurement of cell-free particle growth and formation of precipitate in nGM (normal growth medium), CaPM (normal growth medium spiked with calcium and phosphate to final concentrations of 2.7 mM calcium and 2.5 mM phosphate) and CaPM + $(OEG_2)_2$-IP4. **a**, No difference in pH (37 °C) was observed between treatment groups that were used in VSMC studies. **b**, Turbidity studies do not indicate the development of secondary CPPs in the CaP calcification medium during the VSMC treatment period. **c**,**d**, Dynamic light scattering studies indicate the development of secondary CPPs in the CaP calcification medium after 2 days of incubation (the time point at which treatment medium was replaced) by increase in hydrodynamic diameter (D_h) (**c**) and drop in polydispersity index (PDI) (**d**). No difference between nGM and CaPM was observed. Data are expressed as mean ± s.d. (n = 3 independent experiments). Statistically significant differences were calculated by Student's *t*-test, *** $p < 0.001$ *vs.* nGM.

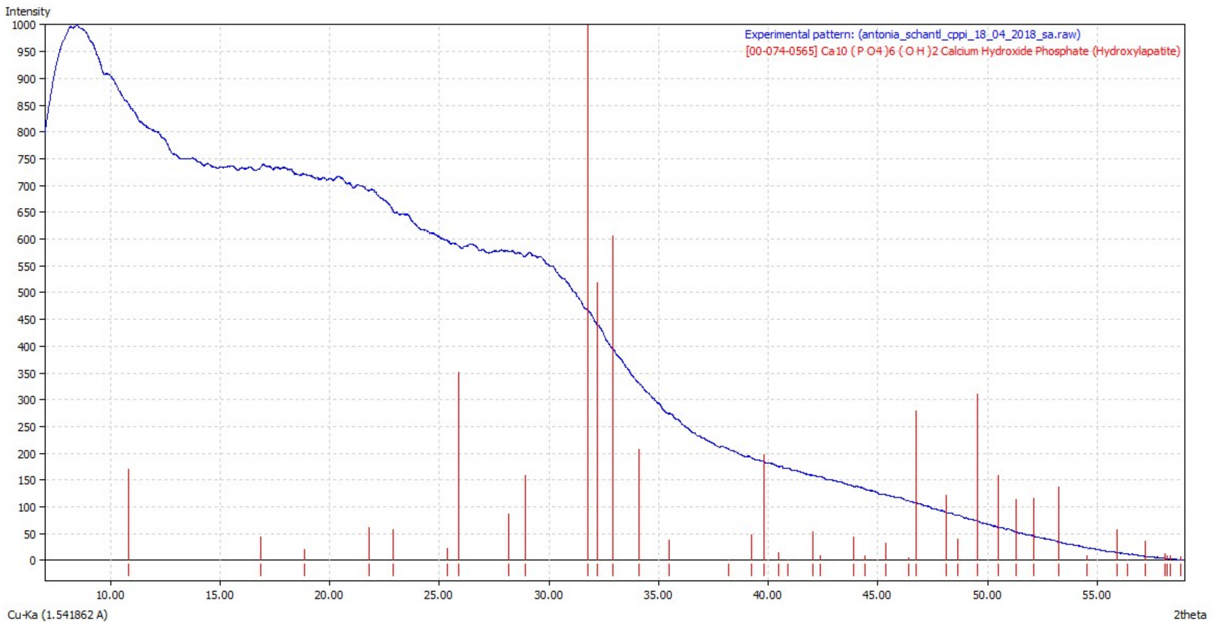

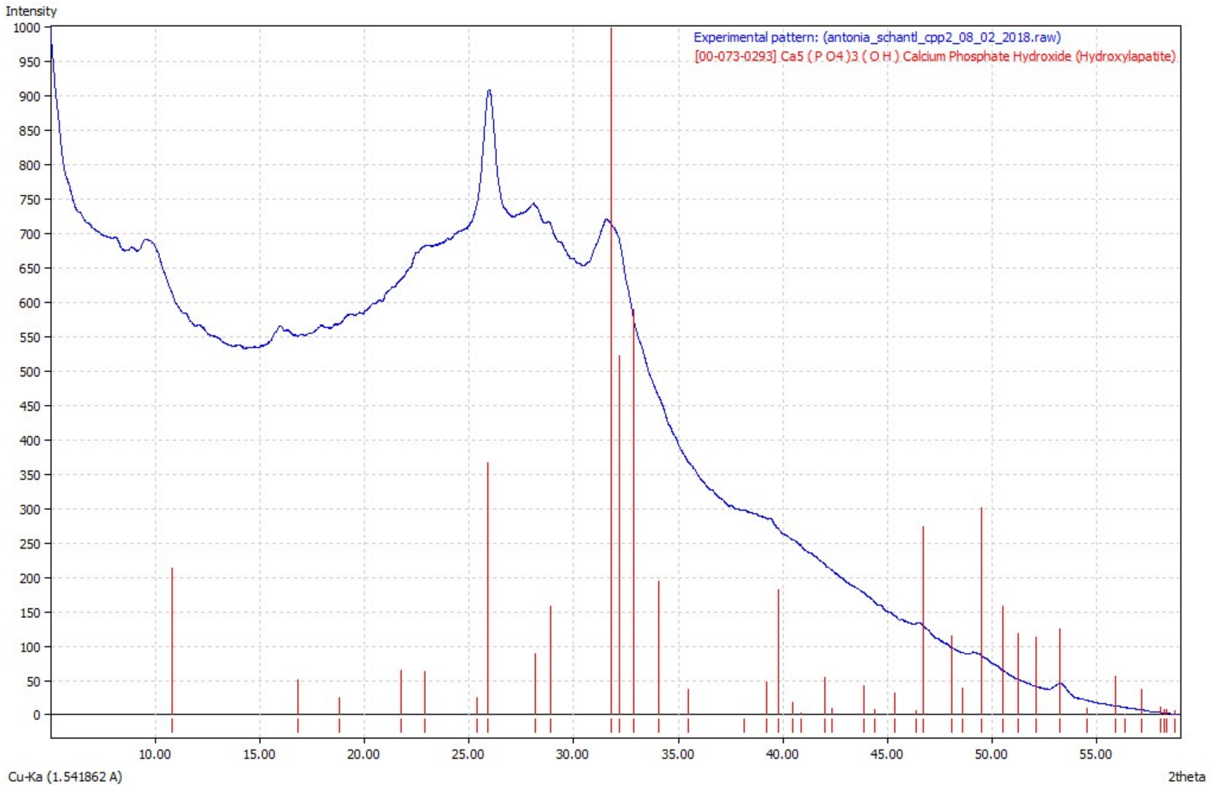

Supplementary Fig. 6: X-ray diffraction (XRD) analysis of CPP1 (top) and CPP2 (bottom). XRD diffraction patterns of CPP2 but not CPP1 correlated with XRD diffraction peaks of hydroxyapatite (in red) indicating presence of crystalline and amorphous material, respectively.

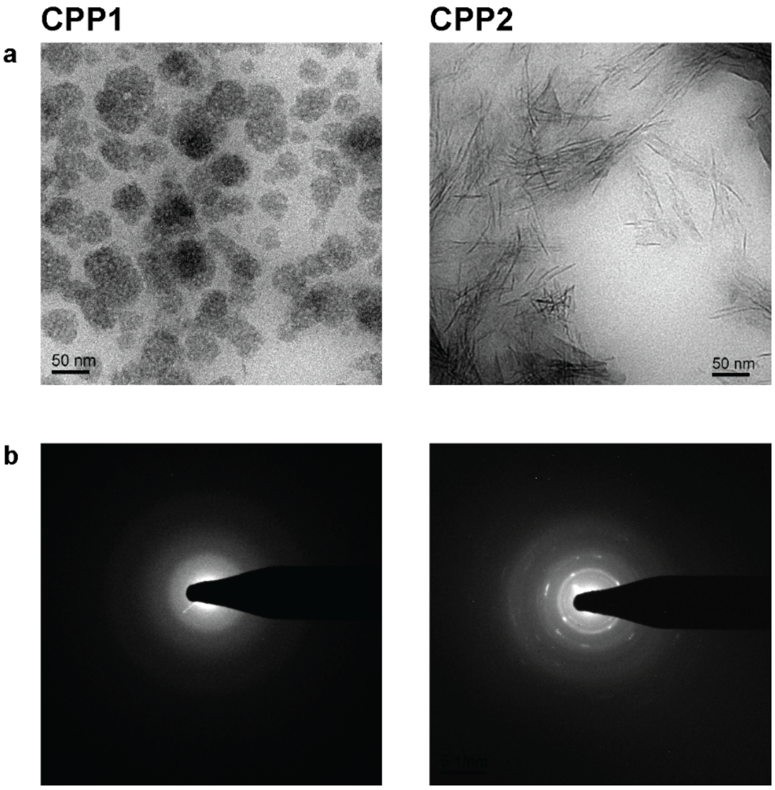

Supplementary Fig. 7: a, Transmission electron microscopy (TEM) images of CPP1 and CPP2. After spiking human serum with calcium (10 mM) and phosphate (6 mM) to initiate the CPP formation, CPP1 were incubated for 1 h, and CPP2 for 14 h. Scale bar = 50 nm **b**, Corresponding selected area electron diffraction (SAED) patterns of TEM images in (**a**). SAED images of CPP2 show bright spots and well defined rings, indicating crystalline material. The derived interplanar- or d-spacings were: d1 = 0.293 nm*, d2 = 0.360 nm†, and d3 = 0.499 nm for CPP2. These distances are in line with reported literature d-spaces for hydroxyapatite: 0.28 nm*, 0.32 nm, 0.34 nm†, 0.47 nm and 0.52 nm[1]. Matching d-spaces between samples are indicated with * and † and suggest identical material.

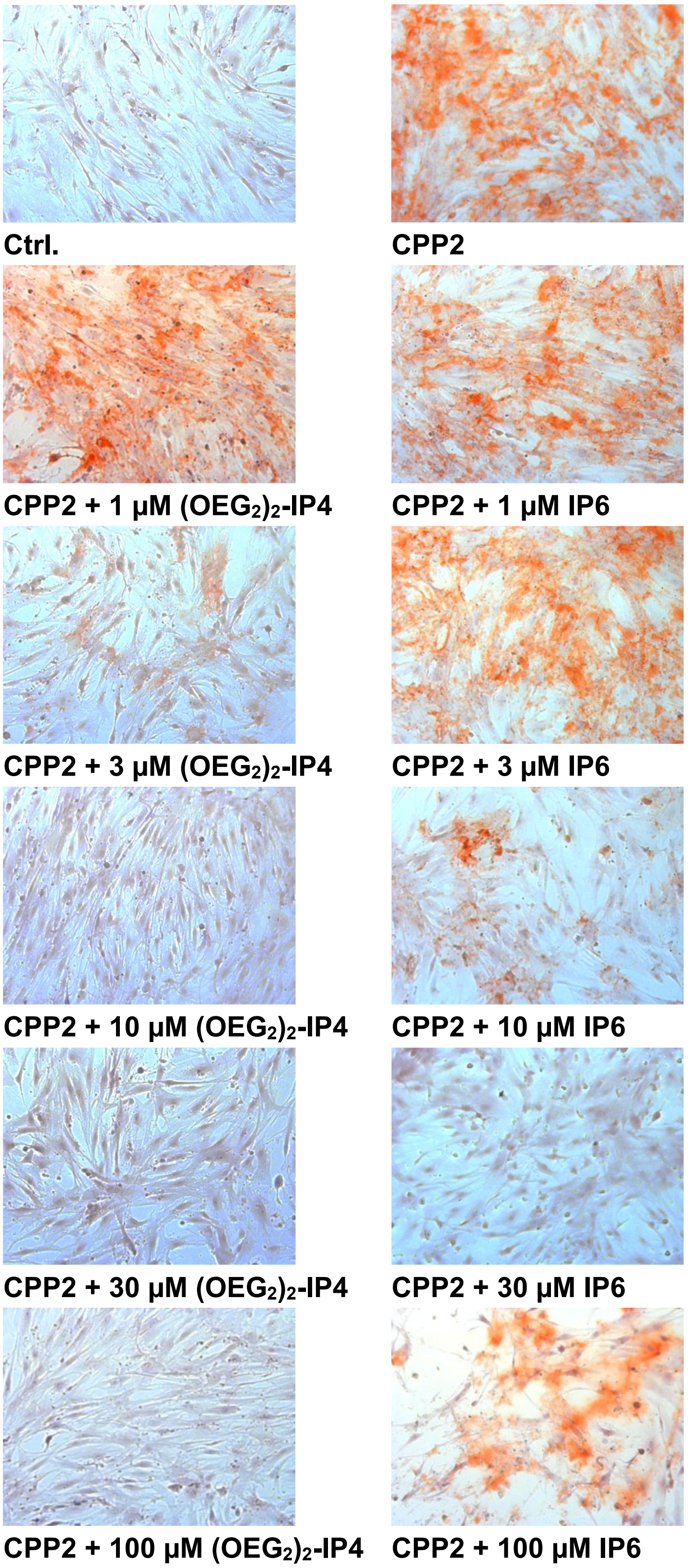

Supplementary Fig. 8: Alizarin Red-S staining for visualization of VSMC monolayer calcification after CPP2 and compound treatment. Treatment of control group was cell culture medium and treatment of CPP2 group was cell culture medium supplemented with 50 µg Ca/mL CPP2. Images were acquired with a Zeiss bright-field microscope at 100 x magnification.

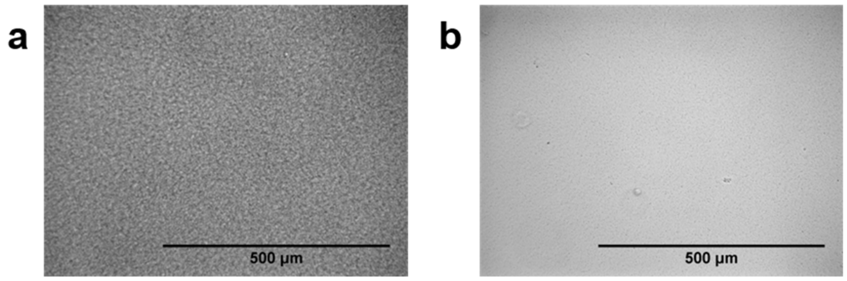

Supplementary Fig. 9: Microscopy images of VSMC after 48 h incubation with CPP medium with 100 µM IP6 (**a**) or 100 µM (OEG$_2$)$_2$-IP4 (**b**). Precipitation occurred in the IP6-containing treatment groups. Images are representative of n = 4 independent experiments (*cf.* Fig. 2e).

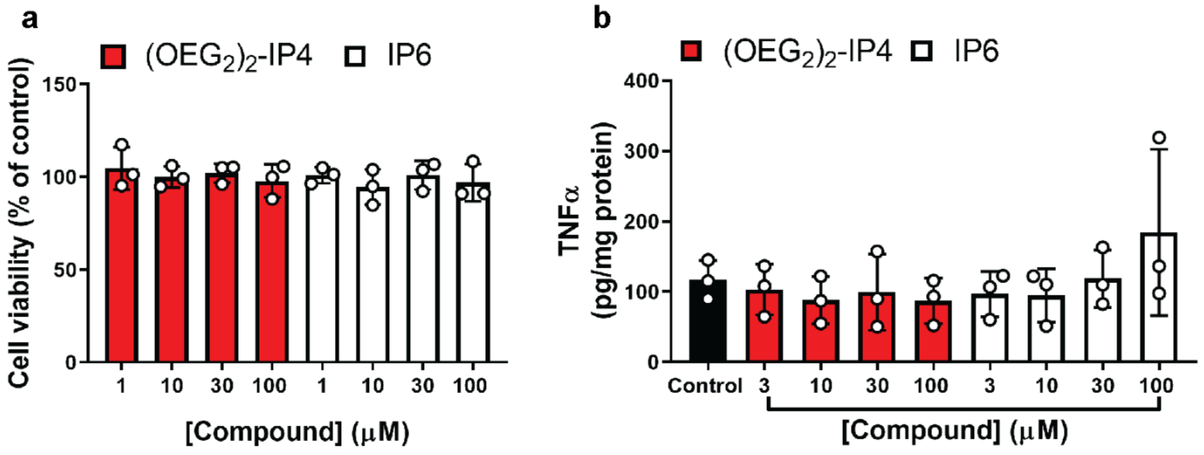

Supplementary Fig. 10: (OEG$_2$)$_2$-IP4 does not alter viability (**a**) or induce the release of pro-inflammatory cytokine TNFα from human THP-1-derived macrophages (**b**). Data are expressed as mean ± s.d. (n = 3 independent experiments).

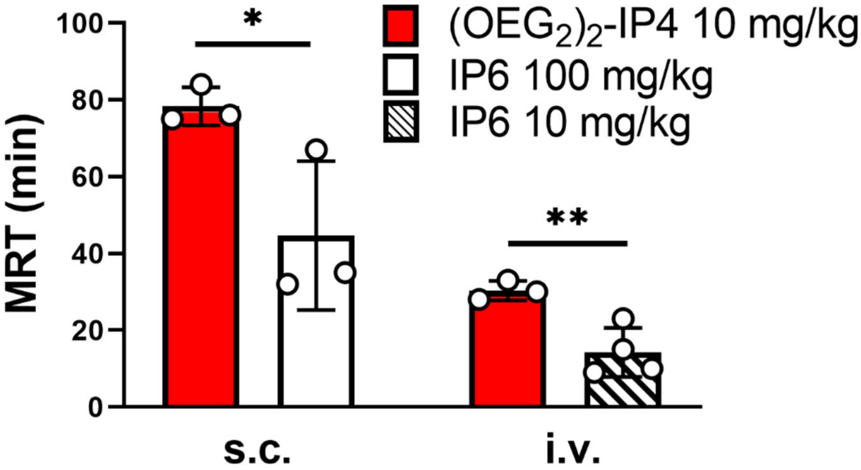

Supplementary Fig. 11: Comparison of the mean residence time (MRT) of $(OEG_2)_2$-IP4 (n = 3) and IP6 (n = 3 for s.c. and n = 4 for i.v. dosing). Data are expressed as mean ± s.d. from n animals. Statistical difference was derived from non-parametric Mann-Whitney test with * $p < 0.05$ and ** $p < 0.01$.

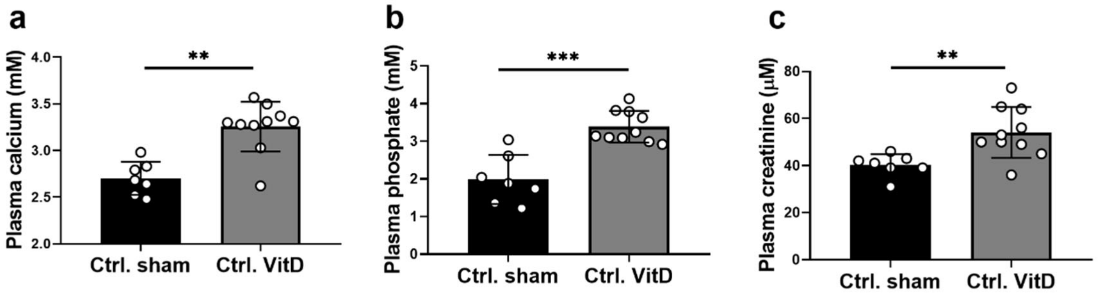

Supplementary Fig. 12: **a**,**b**,**c**, Biochemical and metabolic effects of vitamin D treatment in rats. Each animal received once daily s.c. injection either with a single saline solution (NaCl 0.9% *w/v*, 1 mL/kg, Ctrl. sham, n = 7) or 300,000 IU of vitamin D3 (1 mL/kg, Ctrl. VitD, n = 10) for 5 days. Animals were fed a high-phosphate (1.3%) and low-protein (2.5%) diet. Data are expressed as mean ± s.d. from n animals. Statistical difference was derived from non-parametric Mann-Whitney test with ** $p < 0.01$ and *** $p < 0.001$.

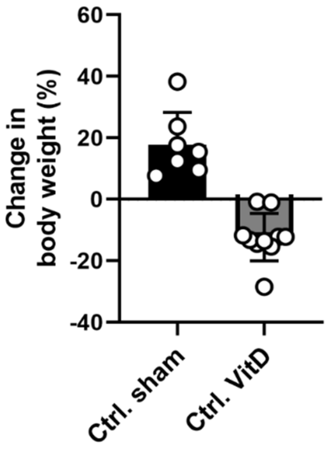

Supplementary Fig. 13: Body weight changes in vitamin D-treated rats. Animals received once daily s.c. injection either with a single saline solution (NaCl 0.9% *w/v*, 1 mL/kg, Ctrl.-sham, n = 7) or 300,000 IU of vitamin D3 (1 mL/kg, Ctrl. VitD, n = 10) for 5 days, and were fed a high-phosphate (1.3%) and low-protein (2.5%) diet and were sacrificed on day 12. Data are expressed as mean ± s.d. from n animals.

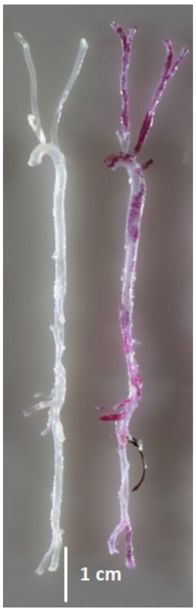

Supplementary Fig. 14: The aortae of vitamin D-treated animals (vitamin D-induced VC rat model) (right) displayed massive calcifications as evident from Alizarin Red staining compared to control (left).

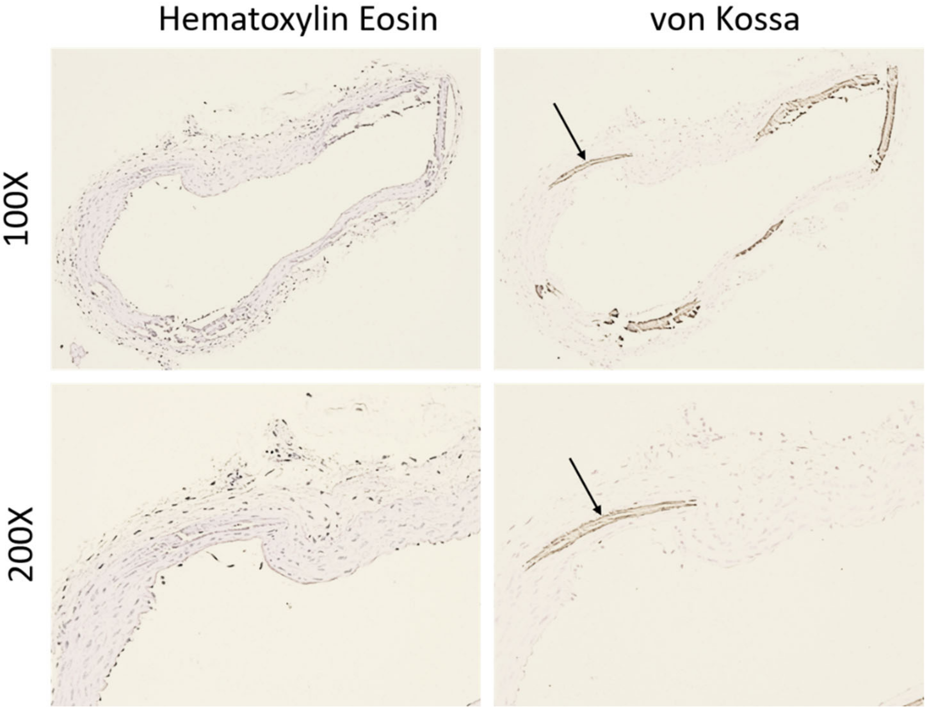

Supplementary Fig. 15: Von Kossa staining on sections of the carotids collected from non-uremic rats with vitamin D3-induced VC, demonstrates that calcified deposits locate primarily in the tunica media (arrows).

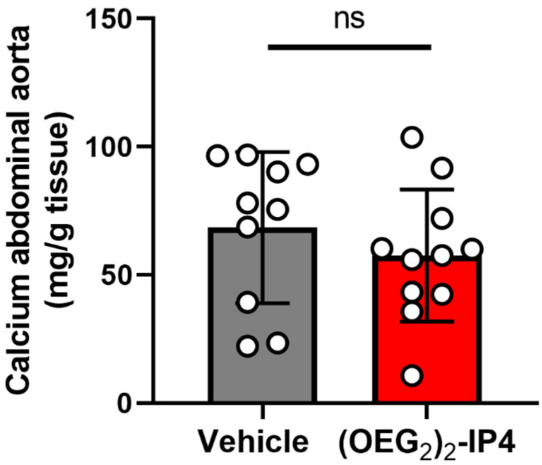

Supplementary Fig. 16: Effect of $(OEG_2)_2$-IP4 treatment on calcium content in the abdominal aorta of vitamin D-induced VC rats. All animals received daily a s.c. injection of 300,000 IU/kg vitamin D3 for 5 days and were fed a high-phosphate (1.3%) and low-protein (2.5%) diet. Concurrently, animals were treated with twice daily injections of vehicle (n = 10) or 30 mg/kg $(OEG_2)_2$-IP4 (n = 11) until they were sacrificed on day 12. Data are expressed as mean ± s.d. from n animals. Statistical difference was derived from non-parametric Mann-Whitney test. NS = not significant.

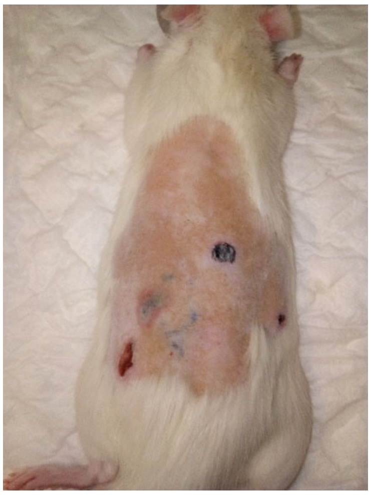

Supplementary Fig. 17: Repeated s.c. administration of 30 mg/kg of IP6 in a volume of 5 mL/kg twice daily (solution was adjusted to pH 6) in different locations on the back of Sprague-Dawley rats (non-uremic vitamin D-induced VC rat model) induced necrotic skin lesions. Therefore, animals had to be sacrificed ahead of schedule on day 10 of the experiment.

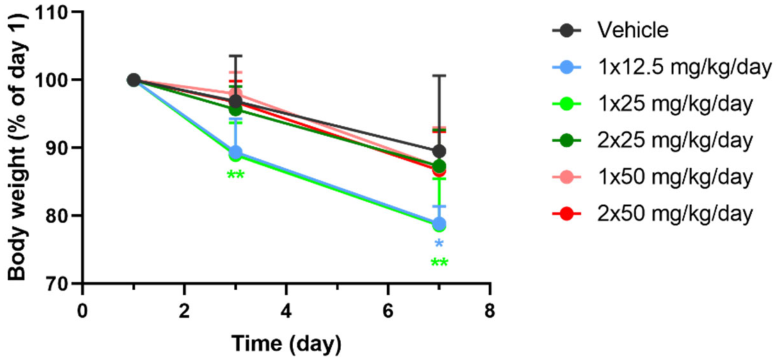

Supplementary Fig. 18: Evolution of the body weight in $(OEG_2)_2$-IP4-treated animals in the vitamin D + warfarin-induced VC model. Treated and untreated animals experienced weight loss on day 7 compared to day 1, which was, however, comparable among treatment groups. Results are expressed as mean ± s.d. with n = 40 for vehicle on day 1 and 3; n = 33 for vehicle on day 7; n = 19 for 1 x 50 mg/kg/day on day 1 and 3 and 2 x 50 mg/kg/day on day 1, 3, n = 18 for 2 x 25 mg/kg/day on day 1, 3, and 7, for 1 x 50 mg/kg/day on day 7, and for 2 x 50 mg/kg/day on day 7; n = 12 for 1 x 12.5 mg/kg/day on day 1, 3 and for 1 x 25 mg/kg/day on day 1,3; n = 8 for 1 x 25 mg/kg/day on day 7 and n = 4 for 1 x 12.5 mg/kg/day on day 7 (from n animals). Statistical difference was derived from non-parametric Kruskal-Wallis test followed by non-parametric Mann-Whitney test with Bonferroni correction, with * $p < 0.05$ and ** $p < 0.01$ *vs*. body weight on day 1.

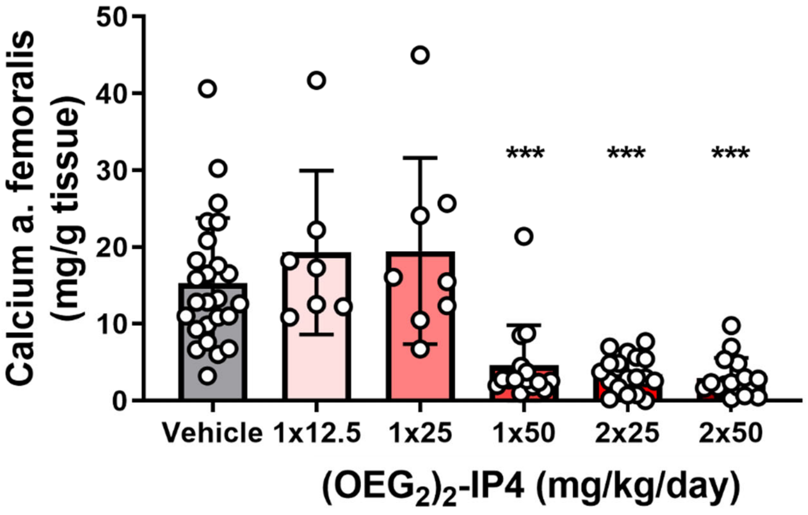

Supplementary Fig. 19. (OEG$_2$)$_2$-IP4 dose-dependently prevents calcification in the femoral artery in a vitamin D-induced rat model of VC (n = 25 for vehicle, n = 7 for 1x12.5 mg/kg/day, n= 8 for 1x25 mg/kg/day, n = 15 for 1x50 mg/kg/day, n = 18 for 2x25 mg/kg/day, and n = 16 for 2x50 mg/kg/day groups, from n animals). Statistical significance was derived from non-parametric Kruskal-Wallis test and multiple comparison was performed by Mann-Whitney test with Bonferroni correction; *** $p < 0.001$ *vs*. vehicle

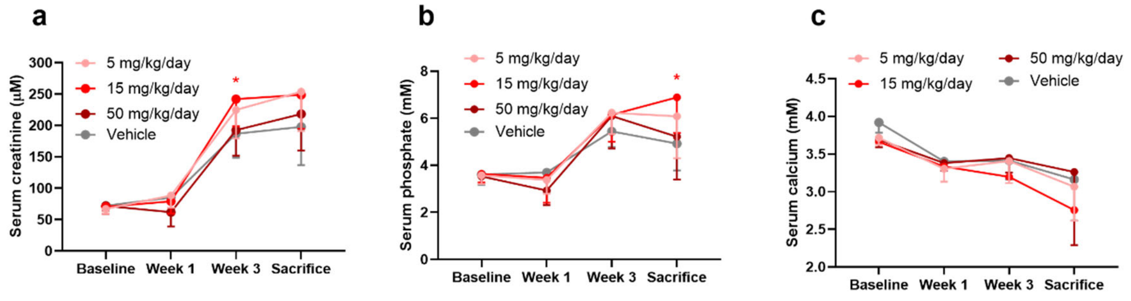

Supplementary Fig. 20: a,**b**,**c**, Biochemical consequences of (OEG$_2$)$_2$-IP4 treatment in the rat model of dietary adenine/high-phosphate-induced uremia with VC presented as serum creatinine (**a**), serum phosphate (n = 11 for vehicle, 15 mg/kg/day and 50 mg/kg dose groups at sacrifice, and n = 12 for all other groups) (**b**), and serum calcium (n = 11 for vehicle and 5 mg/kg/day dose groups at sacrifice, and n = 12 for all other groups) (**c**). Data are expressed as mean ± s.d. from n animals. Statistical difference was derived from non-parametric Kruskal-Wallis test followed by non-parametric Mann-Whitney test with Bonferroni correction with * $p < 0.05$ *vs.* vehicle.

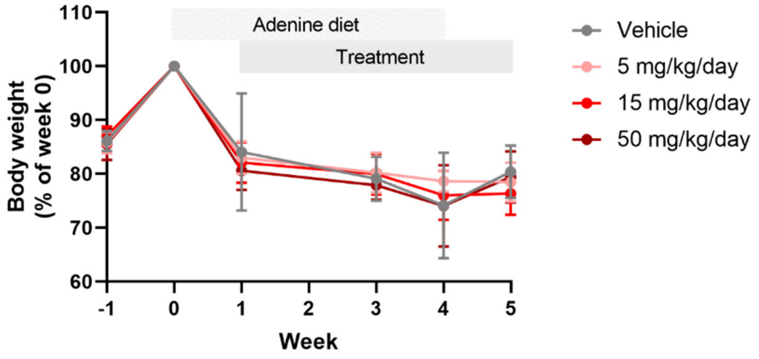

Supplementary Fig. 21: Evolution of the body weight in animals treated with $(OEG_2)_2$-IP4 in the rat model of dietary adenine/high-phosphate-induced uremia and VC. Substantial, but not statistically significant weight loss (51 g on average in one week) occurred as a result of adenine supplementation to the diet. Similar weight losses were observed between different treatment groups. Data are expressed as mean ± s.d. (n = 12 animals). Statistical difference was derived from non-parametric Kruskal-Wallis test followed by non-parametric Mann-Whitney test with Bonferroni correction.

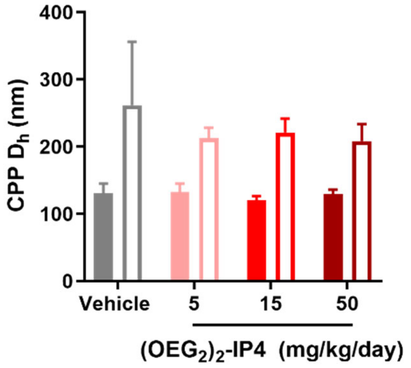

Supplementary Fig. 22: Investigation of the hydrodynamic diameter (D_h) of CPP1 (closed bars) and CPP2 (open bars) derived from sera of vehicle- or $(OEG_2)_2$-IP4-treated rats with dietary adenine/high-phosphate-induced uremia and VC. No statistically significant difference in the hydrodynamic diameter of CPPs was observed between treatment groups. Data are expressed as mean ± s.d. from n animals, with n = 11 for vehicle and the 50 mg/kg/day groups; n = 5 for the 5 and 15 mg/kg/day groups. Statistical difference was derived from ordinary one-way ANOVA followed by Tukey's multiple comparison test.

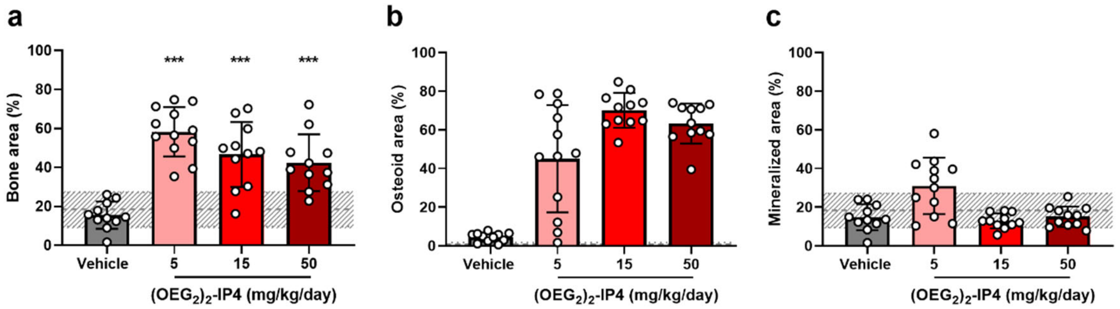

Supplementary Fig. 23: a,**b**,**c**, Effect of $(OEG_2)_2$-IP4 treatment on rat bone structural parameters in the rat model with dietary adenine/high-phosphate-induced uremia and VC, as revealed by histomorphometric quantification. Quantification of bone area (% of total tissue area) (**a**), osteoid area (% of bone area) (**b**) and mineralized area (% of total tissue area) (**c**) revealed that the % bone area was significantly higher in animals treated with $(OEG_2)_2$-IP4 compared with vehicle, which was due to a significant increase in the amount of non-mineralized bone (osteoid) since the amount of mineralized bone was equal in all treatment groups. The grey region in the background presents the values of age-matched non-CKD control animals (mean ± s.d., from n animals). Data are expressed as mean ± s.d. (n = 11 for vehicle, as well as for 15 and 50 mg kg/day and n = 12 for 5 mg/kg/day). Statistical difference was derived from non-parametric Kruskal-Wallis test followed by non-parametric Mann-Whitney test with Bonferroni correction with *** $p < 0.001$ *vs.* vehicle.

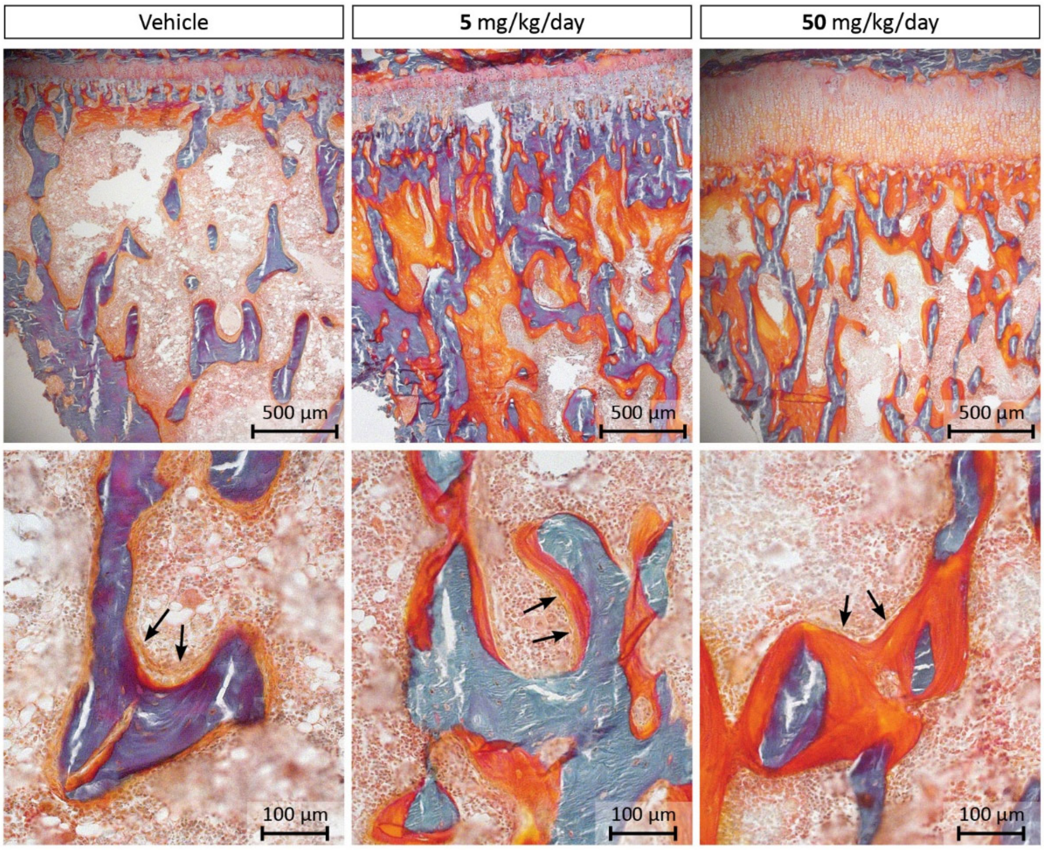

Supplementary Fig. 24: Representative images of Goldner stained tibial sections of the different treatment groups of the experiment using the rat model with dietary adenine/high-phosphate-induced uremia and VC. Non-mineralized bone is stained red, mineralized bone is blue, black arrows indicate healthy, osteoid producing osteoblasts.

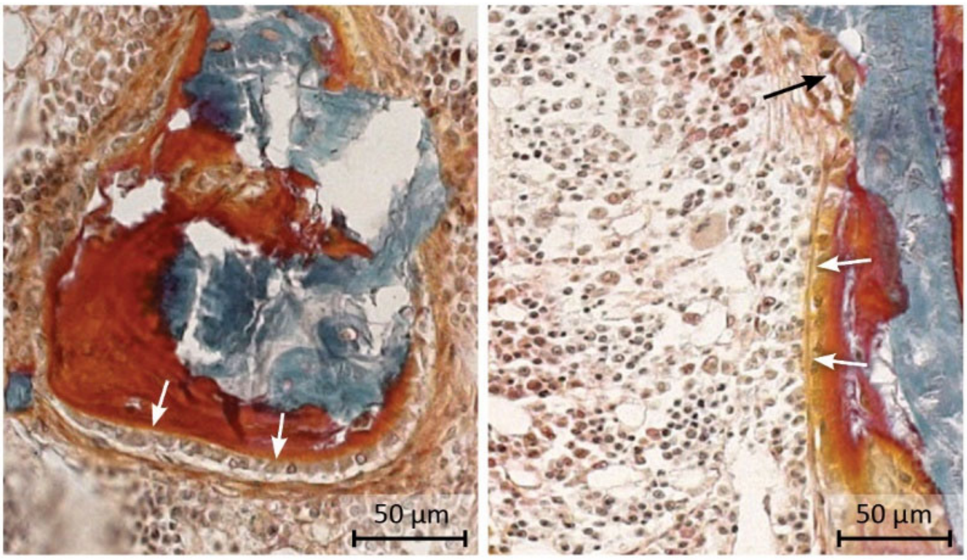

Supplementary Fig. 25: Two representative images of bone cell morphology as seen in rats of the model with dietary adenine/high-phosphate-induced uremia and VC, treated with 50 mg/kg/day $(OEG_2)_2$-IP4. Osteoblasts are indicated by white arrows, osteoclast by black arrows.

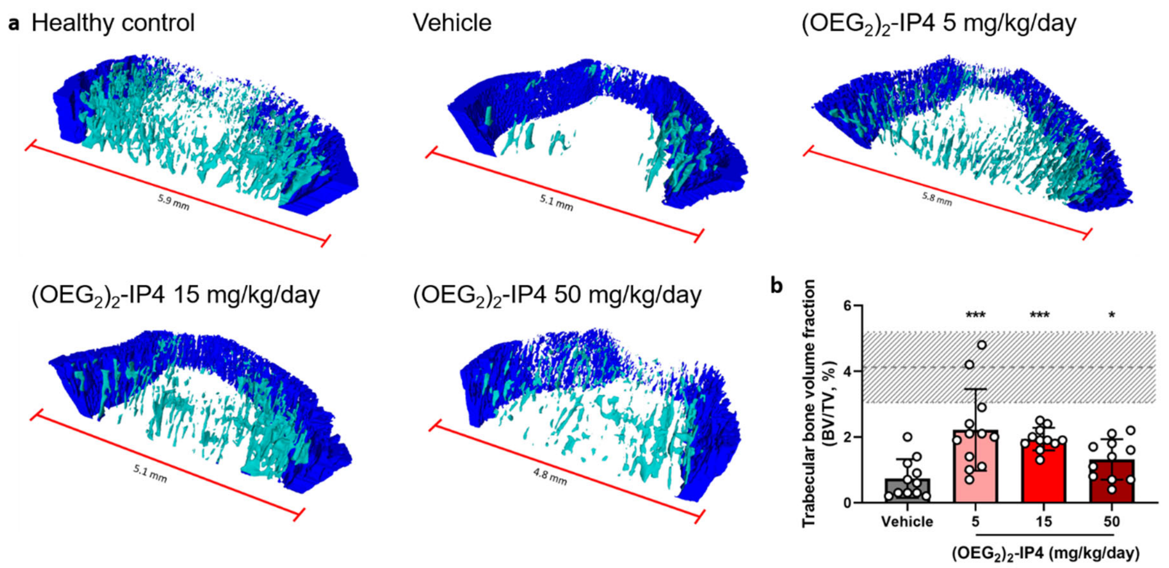

Supplementary Fig. 26: **a**, Effect of CKD (adenine-diet induced) with and without $(OEG_2)_2$-IP4 treatment on rat bone structure as revealed by μCT analysis of mineralized bone structure in proximal tibial metaphysis. Cortical and trabecular bone are colored in blue and green, respectively. **b**, Quantification of the trabecular bone volume fraction. The grey region in the background presents the result of non-CKD (i.e., healthy) control animals (mean ± s.d., from n animals). Data are expressed as mean ± s.d. (n = 12 for 5 mg/kg/day, n = 11 for vehicle and 15 and 50 mg/kg/day and n = 5 for control). Statistical difference was derived from Kruskal-Wallis test followed by non-parametric Mann-Whitney test with * $p < 0.05$ and *** $p < 0.001$ *vs.* vehicle.

2. Supplementary Tables

Supplementary Table 1: Characterization of the synthesized compounds by ESI-QTOF-MS.

**Compound**	**Ion Formula**	**Adduct**	**z**	**Calc. m/z**	**Meas. m/z**	**Err. (ppm)**
OEG_2-IP5	$C_{11}H_{27}NaO_{23}P_5$	M + Na	1+	704.9523	704.9517	0.9
OEG_7-IP5	$C_{21}H_{47}NaO_{28}P_5$	M + Na	1+	925.0834	925.0834	0.0
OEG_{11}-IP5	$C_{29}H_{62}Na_2O_{32}P_5$	M + Na	1+	1123.1702	1123.1672	-2.7
OEG_{12}-IP5	$C_{31}H_{66}Na_2O_{33}P_5$	M + Na	1+	1167.1964	1167.1953	-0.9
$(OEG_2)_2$-IP4	$C_{16}H_{36}NaO_{22}P_4$	M + Na	1+	727.0541	727.0537	0.0
$(OEG_7)_2$-IP4	$C_{36}H_{76}Na_2O_{32}P_4$	M + Na	2+	595.1527	595.1515	-2.0
$(OEG_{11})_2$-IP4	$C_{52}H_{107}Na_4O_{40}P_4$	M + Na	3+	529.1621	529.1622	0.2
OEG_{11}-IP2S3	$C_{29}H_{59}Na_2O_{32}P_2S_3$	M + Na	1+	1123.1417	1123.1412	-0.4
$(OEG_2)_3$-IP3	$C_{21}H_{45}NaO_{21}P_3$	M + H	1+	749.1558	749.1555	0.4
$(OEG_7)_3$-IP3	$C_{51}H_{105}NaO_{36}P_3$	M + H	1+	1409.5491	1409.5466	1.8

Supplementary Table 2: PK parameters of compounds after s.c. administration into healthy rats. Data are expressed as mean ± s.d..

	$(OEG_2)_2$-IP4		IP6	
Dose (mg/kg)	10	50	10	100
$t_{1/2}$ (min)	47 ± 1	190 ± 66	NA	29 ± 15
C_{max} (μM)	12 ± 1	71 ± 26	< LLOQ	33 ± 10
$AUC_{0\text{-}t}$ (μM·min)	1188 ± 36	7176 ± 2598	NA	1165 ± 76
t_{last} (min)	360	1440	NA	360
$AUC_{0\text{-}\infty}$ (μM·min)	1197 ± 36	7296 ± 2724	NA	1346 ± 149
n	3	4	3	3

NA = not applicable, LLOQ = lower limit of quantification = 772 nM

Supplementary Table 3: PK parameters of $(OEG_2)_2$-IP4 following s.c. administration of 50 mg/kg into rats with vitamin D-induced VC and control. Data are expressed as mean ± s.d. (n = 4).

	Healthy	Cardiovascular calcification model
$t_{1/2}$ (min)	235 ± 16	224 ± 44
C_{max} (μM)	49 ± 7	34 ± 8
$AUC_{0\text{-}1440}$ (μM·min)	6049 ± 799	9077 ± 2455
$AUC_{0\text{-}\infty}$ (μM·min)	6082 ± 792	9146 ± 2428
CL/F_{obs} (mL/min/kg)	11 ± 1	7 ± 2
$MRT_{0\text{-}1440}$ (min)	136 ± 11	210 ± 2

Supplementary Table 4: PK parameters of $(OEG_2)_2$-IP4 following i.v. bolus administration of 10 mg/kg into healthy and adenine and high-phosphate diet-induced VC rats (uremic), respectively. Data are expressed as mean ± s.d. (n = 6).

	Healthy	Uremic
$t_{1/2}$ (min)	52 ± 14	83 ± 47
C_{max} (μM)	35 ± 9	43 ± 12
$AUC_{0\text{-}240}$ (μM·min)	1068 ± 841	2220 ± 503
$AUC_{0\text{-}\infty}$ (μM·min)	1110 ± 934	2734 ± 891
$MRT_{0\text{-}240}$ (min)	26 ± 19	53 ± 9
CL (mL/min/kg)	17.9 ± 7.7	5.8 ± 2.0

Supplementary Table 5: PK parameters of $(OEG_2)_2$-IP4 following s.c. administration of 10 mg/kg into healthy and adenine and high-phosphate diet-induced VC rats (uremic). Data are expressed as mean ± s.d. (n = 6).

	**Healthy**	**Uremic**
$t_{1/2}$ (min)	79 ± 27	123 ± 64
C_{max} (μM)	12 ± 4	12 ± 4
$AUC_{0\text{-}360}$ (μM•min)	1152 ± 842	1906 ± 511
$AUC_{0\text{-}\infty}$ (μM•min)	1182 ± 876	2273 ± 694
MRT (min)	66 ± 19	124 ± 28

Supplementary Table 6: Biochemistry and bodyweight in a rat model of vitamin D-induced VC (5 days s.c. 300,000 UI/mL/day + high-phosphate (1.3%) and low-protein (2.5%) diet). Statistical difference was derived from non-parametric Mann-Whitney test.

**Parameters**	**Vehicle (n = 10)**	**$(OEG_2)_2$-IP4 (n = 11)**	***P* value**
Creatinine (mM)	54.1 ± 10.8	39.8 ± 6.6	< 0.001
Total calcium (mM)	3.40 ± 0.12	3.28 ± 0.15	NS
Phosphate (mM)	2.79 ± 0.39	2.61 ± 0.34	NS
Change in body weight (g)	-41 ± 20	-38 ± 7	NS
Final body weight (g)	248 ± 19	232 ± 12	< 0.05

NS = not significant, *p* value *vs.* vehicle

Supplementary Table 7: Survival data of $(OEG_2)_2$-IP4 treated animals from the adenine and high-phosphate diet-induced VC model.

**Intervention (mg/kg/day)**	**Mortality (animals)**
Vehicle	1/12
5	0/12
15	1/12
50	1/12

Supplementary Table 8: Quantitation of the von Kossa staining of aorta thoracalis samples from $(OEG_2)_2$-IP4-treated rats of the adenine and high-phosphate diet-induced VC model. Data are expressed as mean ± s.d..

Intervention	n	Von Kossa positive area in the aorta thoracalis (%)
Vehicle	11	14.50 ± 15.11
5 mg/kg/day	12	17.86 ± 12.46
15 mg/kg/day	11	14.35 ± 11.49
50 mg/kg/day	11	2.72 ± 5.97

Supplementary Table 9: Total calcium content in cardiovascular tissues of $(OEG_2)_2$-IP4-treated rats in the adenine and high-phosphate diet-induced VC model. Data are expressed as mean ± s.d..

Intervention	n	Calcium (mg/g tissue)			
		Heart	A. carotis	A. abdominialis	A. femoralis
Vehicle	11	0.08 ± 0.05	9.55 ± 13.35	14.45 ± 13.89	5.29 ± 8.78
5 mg/kg/day	12	0.05 ± 0.03	11.06 ± 9.10	12.71 ± 11.66	4.47 ± 3.95
15 mg/kg/day	11	0.04 ± 0.01	5.07 ± 6.23	6.24 ± 3.70	4.14 ± 1.92
50 mg/kg/day	11	0.04 ± 0.02	0.84 ± 1.11	1.69 ± 1.80	2.01 ± 1.41

Supplementary Table 10: Trabecular bone parameters assessed from e*x vivo* μCT skeletal morphology of the proximal metaphysis of the left tibia from rats of CKD (adenine-induced) VC model at week 5 (sacrifice). Data are expressed as mean ± s.d. (n = 12 for 5 mg/kg/day, n = 11 for vehicle and 15 and 50 mg/kg/day and n = 5 for control). Statistical difference was derived from Kruskal-Wallis test followed by non-parametric Mann-Whitney test *vs.* vehicle. Data from control age-matched (non-CKD) rats are also included in the table.

	**Control (non-CKD)**	**Vehicle**	**$(OEG_2)_2$-IP4 (mg/kg/day)**		
			**5**	**15**	**50**
Trabecular bone volume fraction (BV/TV, %)	4.12 ± 1.07	0.74 ± 0.59	2.22 ± 1.24 ($p < 0.001$)	1.94 ± 0.34 ($p < 0.001$)	1.32 ± 0.61 ($p < 0.05$)
Trabecular number (Tb.N., mm^{-1})	0.62 ± 0.12	0.13 ± 0.09	0.42 ± 0.20 ($p < 0.001$)	0.44 ± 0.09 ($p < 0.001$)	0.32 ± 0.14 ($p < 0.01$)
Trabecular thickness (Tb.Th., μm)	67 ± 10	58 ± 7	51 ± 7 ($p < 0.05$)	44 ± 2 ($p < 0.001$)	41 ± 3 ($p < 0.001$)
Trabecular separation (Tb.S., μm)	514 ± 114	916 ± 63	662 ± 135 ($p < 0.001$)	588 ± 106 ($p < 0.001$)	627 ± 123 ($p < 0.001$)
Bone mineral density (BMD, mg/cm^2)	356 ± 14	356 ± 7	359 ± 9	362 ± 5	362 ± 14

Supplementary Table 11: Experimental results and biochemical assays used to assess potential off-target activity of 10 μM $(OEG_2)_2$-IP4.

Assay name	Catalog number	Species	% Inhibition
ATPase, Na+/K+, Heart, Pig	107710	Pig	6
Cholinesterase, Acetyl, ACES	104010	Human	5
Cyclooxygenase COX-1	116020	Human	-2
Cyclooxygenase COX-2	118010	Human	8
Monoamine Oxidase MAO-A	140010	Human	3
Monoamine Oxidase MAO-B	140120	Human	0
Peptidase, Angiotensin Converting Enzyme	107300	Rabbit	-23
Peptidase, CTSG (Cathepsin G)	112510	Human	23
Phosphodiesterase PDE3	152000	Human	-1
Phosphodiesterase PDE4	154000	Human	12
Protein Serine/Threonine Kinase, PKC, Non-selective	178010	Rat	-18
Protein Tyrosine Kinase, Insulin Receptor	174990	Human	-22
Protein Tyrosine Kinase, LCK	176020	Human	4
Adenosine A_1	200510	Human	4
Adenosine A_{2A}	200610	Human	5
Adenosine α_{1A}	203100	Rat	1
Adenosine α_{1B}	203200	Rat	5
Adenosine α_{1D}	203400	Human	10
Adenosine α_{2A}	203630	Human	14
Adenosine α_{2B}	203710	Human	3
Adrenergic β_1	204010	Human	2
Adrenergic β_2	204110	Human	5
Androgen (Testosterone)	206000	Human	12
Angiotensin AT_1	210030	Human	0
Bradykinin B_2	212320	Human	-17
Calcium Channel L-Type, Benzothiazepine	214510	Rat	-7
Calcium Channel L-Type, Dihydropyridine	214600	Rat	1
Calcium Channel L-Type, Phenylalkylamine	215000	Rat	-4
Calcium Channel N-Type	216000	Rat	5
Cannabinoid CB_1	217030	Human	-7
Cannabinoid CB_2	217100	Human	12
Chemokine CCR1	217510	Human	-2
Chemokine CXCR2 (IL-$8R_B$)	244500	Human	6
Cholecystokinin CCK_1 (CCK_A)	218030	Human	6
Cholecystokinin CCK_2 (CCK_B)	218130	Human	-6
Dopamine D_1	219500	Human	-1
Dopamine D_{2L}	219500	Human	-10
Dopamine D_{2S}	219700	Human	7
Endothelin ET_A	224010	Human	8
Estrogen ERα	226010	Human	-1
$GABA_A$, Chloride Channel, TBOB	226810	Rat	-19
$GABA_A$, Flunitrazepam, Central	226600	Rat	-7
$GABA_A$, Ro-15-1788, Hippocampus	226630	Rat	5

$GABA_{B1A}$	228610	Human	-7
Glucocorticoid	232030	Human	5
Glutamate, AMPA	232600	Rat	8
Glutamate, Kainate	232710	Rat	17
Glutamate, Metabotropic, $mGlu_5$	237000	Human	24
Glutamate, NMDA, Agonism	232810	Rat	7
Glutamate, NMDA, Glycine	232910	Rat	6
Glutamate, NMDA, Phencyclidine	233000	Rat	7
Glutamate, NMDA, Polyamine	234000	Rat	17
Glycine, Strychnine-Sensitive	239000	Rat	-2
Histamine H_1	239610	Human	4
Histamine H_2	239710	Human	-6
Leukotriene, Cysteinyl $CysLT_1$	250460	Human	0
Melanocortin MC_1	251100	Human	9
Melanocortin MC_4	251100	Human	3
Muscarinic M_1	252610	Human	-6
Muscarinic M_2	252710	Human	4
Muscarinic M_3	252810	Human	-6
Muscarinic M_4	252910	Human	1
Neuropeptide Y Y_1	257010	Human	-9
Nicotinic Acetylcholine	258590	Human	-5
Nicotinic Acetylcholine α1, Bungarotoxin	258700	Human	18
Opiate δ_1 (OP1, DOP)	260130	Human	-2
Opiate κ (OP2, KOP)	260210	Human	1
Opiate μ (OP3, MOP)	260410	Human	11
Platelet Activating Factor (PAF)	265010	Human	-7
Potassium Channel [K_{ATP}]	265600	Hamster	14
Potassium Channel hERG	265900	Human	11
PPARγ	267500	Human	2
Progesterone PR-B	299005	Human	-4
Serotonin (5-Hydroxytryptamine) $5\text{-}HT_{1A}$	271110	Human	11
Serotonin (5-Hydroxytryptamine) $5\text{-}HT_{1B}$	271230	Human	-4
Serotonin (5-Hydroxytryptamine) $5\text{-}HT_{2A}$	271650	Human	-1
Serotonin (5-Hydroxytryptamine) $5\text{-}HT_{2B}$	271700	Human	1
Serotonin (5-Hydroxytryptamine) $5\text{-}HT_{2C}$	271800	Human	-5
Serotonin (5-Hydroxytryptamine) $5\text{-}HT_3$	271910	Human	8
Sodium Channel, Site 2	279510	Rat	-1
Tachykinin NK_1	255520	Human	-14
Transporter, Adenosine	202000	Guinea pig	-14
Transporter, Dopamine (DAT)	220320	Human	19
Transporter, GABA	226400	Rat	4
Transporter, Norepinephrine (NET)	204410	Human	20
Transporter, Serotonin (5-Hydroxytryptamine) (SERT)	274030	Human	2
Vasopressin V_{1A}	287560	Human	-6

Supplementary Table 12: Compound register.

Abbreviation	Chemical name	MW of anion (g/mol)	Salt
IP6	*myo*-Inositol hexakisphosphate	647.94	12 Na
IP5	*myo*-Inositol-1,2,3,5,6-pentakisphosphate	569.98	5 Na
IP4	*myo*-Inositol-2,3,5,6-tetrakisphosphate	492.01	4 Na
IP3	*myo*-Inositol-1,4,5-triphosphate	414.05	3 Na
IP2	*myo*-Inositol-2,4-diphosphate	336.08	2 Na
IT6	*myo*-Inositol hexathiophosphate	750.31	6 Na
OEG_2-IP5	(±)-6-*O*-(methoxy-diethyleneglycol)-*myo*-inositol-1,2,3,4,5-pentakis(phosphate)	672.11	10 Na
OEG_7-IP5	(±)-6-*O*-(methoxy-heptaethyleneglycol)-*myo*-inositol-1,2,3,4,5-pentakis(phosphate)	892.37	10 Na
OEG_{11}-IP5	(±)-6-*O*-(methoxy-undecaethyleneglycol)-*myo*-inositol-1,2,3,4,5-pentakis(phosphate)	1068.59	10 Na
OEG_{12}-IP5	(±)-6-*O*-(methoxy-dodecaethyleneglycol)-*myo*-inositol-1,2,3,4,5-pentakis(phosphate)	1112.64	10 Na
$(OEG_2)_2$-IP4	4,6-Di-*O*-(methoxy-diethyleneglycol)-*myo*-inositol-1,2,3,5-tetrakis(phosphate)	696.28	8 Na/ 4 Na*
$(OEG_7)_2$-IP4	4,6-Di-*O*-(methoxy-heptaethyleneglycol)-*myo*-inositol-1,2,3,5-tetrakis(phosphate)	1136.81	8 Na
$(OEG_{11})_2$-IP4	4,6-Di-*O*-(methoxy-undecaethyleneglycol)-*myo*-inositol-1,2,3,5-tetrakis(phosphate)	1489.23	8 Na
OEG_{11}-IP2S3	(±)-4-*O*-(methoxy-undecaethyleneglycol)-1,3,5-*O*-tris(sulfato)-*myo*-inositol-2,6-*O*-bis(phosphate)	1071.84	7 Na
$(OEG_2)_3$-IP3	2,4,6-Tri-*O*-(methoxy-diethyleneglycol)-*myo*-inositol-1,3,5-tris(phosphate)	720.45	2 Mg
$(OEG_7)_3$-IP3	2,4,6-Tri-*O*-(methoxy-heptaethyleneglycol)-*myo*-inositol-1,3,5-tris(phosphate)	1381.24	3 Na

* The 8·Na salt was used in the screening assay and the 4·Na salt in all subsequent assays.

Supplementary Table 13: Description of PK parameters derived from non-compartmental analysis.

Abbreviation	Definition
**C_{max}**	Maximum plasma concentration after single administration
**t_{max}**	Time of maximum plasma concentration
**$t_{1/2}$**	Biological half-life
**AUC_{0-t}**	Area under the curve from time zero to the time of the last quantifiable plasma concentration
**$AUC_{0-\infty}$**	AUC from time zero extrapolated to infinity
**MRT**	Mean Residence Time

Supplementary Table 14: Chromatographic gradient applied for $(OEG_2)_2$-IP4.

Time (min)	Mobile phase B (%)	Flow (mL/min)
0.5	2	0.3
1.0	2	0.3
2.0	98	0.3
3.5	98	0.3
4.0	2	0.3
6.0	2	0.1

Supplementary Table 15: Chromatographic gradient applied for IP6.

Time (min)	Mobile phase B (%)	Flow (mL/min)
0.01	2	0.3
0.5	2	0.3
1.0	20	0.3
2.0	20	0.3
2.5	98	0.3
3.0	98	0.3
3.5	2	0.3
4.7	2	0.3

Supplementary Table 16: MS/MS parameters for $(OEG_2)_2$-IP4. OEG_{11}-IP5 was used as internal standard.

Compound	Precursor ion (Q1) (*m/z*)	Product ion (Q3) (*m/z*)	Dwell time (ms)	CE (eV)
$(OEG_2)_2$-IP4	703.1	525.1	300	-46
$(OEG_2)_2$-IP4	703.1	605.1	300	-33
$(OEG_2)_2$-IP4	703.1	158.8	300	-45
OEG_{11}-IP5	1077.3	881.2	300	-61
OEG_{11}-IP5	1077.3	899.2	300	-59
OEG_{11}-IP5	1077.3	979.3	300	-51

Supplementary Table 17: MS/MS parameters for IP6.

Compound	Precursor ion (Q1) (*m/z*)	Product ion (Q3) (*m/z*)	Dwell time (ms)	CE (eV)
IP6	659.0	560.8	300	-46
IP6	659.0	480.8	300	-48
IP6	659.0	463.1	300	-46

Supplementary Table 18: UHPLC gradient applied for $(OEG_2)_2$-IP4.

Time (min)	Mobile phase B (%)
0.0	75
3.0	30
5.0	75

Supplementary Table 19: MS/MS parameters for $(OEG_2)_2$-IP4 of the optimized bioanalytical method. OEG_{11}-IP5 was used as internal standard.

Compound	Precursor ion (Q1) (*m/z*)	Product ion (Q3) (*m/z*)	CE (V)	RF Lens (V)
$(OEG_2)_2$-IP4	703.1	158.9	39	
$(OEG_2)_2$-IP4	703.1	507.1	33	
$(OEG_2)_2$-IP4	703.1	525.1	32	118
$(OEG_2)_2$-IP4	703.1	587.1	30	
$(OEG_2)_2$-IP4	703.1	605.7	23	
OEG_{11}-IP5	1077.3	800.7	49	
OEG_{11}-IP5	1077.3	881.2	42	
OEG_{11}-IP5	1077.3	899.1	37	118
OEG_{11}-IP5	1077.3	960.3	38	
OEG_{11}-IP5	1077.3	979.1	31	

3. Supplemental Methods

3.1. Chemistry

3.1.1. Synthesis of (±)-6-*O*-(methoxy-dodecaethyleneglycol)-*myo*-inositol-1,2,3,4,5-pentakis(phosphate) ((±)-OEG_{12}-IP5)

Compound **(±)-2** (Supplemental Methods Fig. 1). Compound **1** 1,3,5-*O*-methylidyne-*myo*-inositol (651 mg, 3.4 mmol, 1.0 eq.) was added to a suspension of NaH (163 mg, 60% in mineral oil, 4.1 mmol, 1.2 eq.) in 6 mL DMF and stirred for 30 min. Subsequently, a solution of CH_3-$(OCH_2CH_2)_{12}$-OTs (OEG_{12}-OTs, 4.1 mmol, 1.2 eq.) in DMF was added and stirred overnight, after which the reaction was quenched with water and extracted with DCM. The organic layer was washed with brine and dried over Na_2SO_4. The mixture was purified by column chromatography using a 0-15% MeOH/DCM gradient. The crude compound was then dissolved in 1 mL of DCM and a solution of 0.2 mL water in 5 mL TFA was added slowly to the reaction mixture, which was then stirred for 35 min at room temperature (RT) before it was co-evaporated with toluene. The resulting residue was purified by column chromatography using a 1-30% MeOH/DCM gradient to afford 168 mg of product. ^{1}H-NMR (400 MHz; D_2O): δ 4.02 (t, J = 2.9 Hz, 1H), 3.98-3.89 (m, 2H), 3.73-3.65 (m, 43H), 3.63-3.56 (m, 4H), 3.50-3.44 (m, 2H), 3.36-3.31 (m, 4H).

Compound **(±)-3**. Compound **(±)-2** (0.23 mmol, 1.0 eq.) and tetrazole (5.7 mmol, 25.0 eq.) were combined in anhydrous DCM (12 mL) and dried over molecular sieves overnight. *o*-Xylylene-*N*,*N*-diethylphosphoramidite (*o*XEP) (617 mg, 2.57 mmol, 11.0 eq.) was added and left to react for 48 h at RT. Subsequently, *o*XEP (224 mg, 0.9 mmol, 4.0 eq.), tetrazole (1.3 mmol, 5.9 eq.) and anhydrous DCM (3 mL) were added and the mixture was allowed to react for 48 h at RT. *m*-chloro-perbenzoic acid (*m*-CPBA, 11.6 mmol, 50 eq) was dissolved in DCM and dried over Na_2SO_4. The dried *m*-CPBA solution was added dropwise to the reaction mixture and left to react for 20 min at -10 °C and then for 40 min at 0 °C, after which the reaction was quenched with Na_2SO_3. The reaction mixture was extracted with DCM and washed with 1 M $Na_2S_2O_4$, water, saturated $NaHCO_3$, and brine. The aqueous layer was backwashed with DCM. The combined organic layer was dried over Na_2SO_4, and the crude product was purified *via* column chromatography using a gradient of 1%-20% MeOH/DCM, yielding 30 %. ^{1}H-NMR (400 MHz, chloroform-*d*) δ 7.42 – 7.27 (m, 14H), 5.72 – 4.66 (m, 25H), 3.68 – 3.52

(m, 39H), 3.48 (s, 2H, MeOH), 3.37 (s, 3H). ^{13}C-NMR (126 MHz, chloroform-*d*) δ 136.04 – 134.26 (m), 129.42 – 128.44 (m), 71.93, 70.76 – 70.21 (m), 59.03, 53.47. ^{31}P-NMR (162 MHz, chloroform-*d*) δ -0.60, -1.88, -2.01, -3.05, -4.12.

Compound **4** (**(±)-OEG$_{12}$-IP5,** Supplemental Methods Fig. 1). Compound **(±)-3** (0.055 mmol, 1.0 eq.) was dissolved in MeOH (7.5 mL) and water (0.75 mL). The solution was placed under a hydrogen atmosphere (1 atm) over Pd/C catalyst for 48 h at RT. The mixture was filtered over celite and washed with water, MeOH, and diluted aqueous triethylamine. The crude was purified *via* reverse phase chromatography (Strata C-18 columns, 1 g, 70 Å), and size exclusion chromatography (GE Healthcare, PD-10 Columns, 8.3 mL Sephadex, G-25 resin). The sodium salt was prepared by eluting an aqueous solution through a column packed with Dowex 50WX8 and the final sodium form was purified with a PD-10 column. The product is obtained as a distribution of oligoethylene glycols with an average of 12 units. ^{1}H-NMR (500 MHz; D_2O): δ 4.89-4.87 (m, 1H), 4.49-4.43 (m, 2H), 4.26-4.14 (m, 4H), 3.92-3.85 (m, 3H), 3.80-3.74 (m, 2H), 3.70-3.48 (m, 49H), 3.28 (s, 3H). ^{31}P-NMR (162 MHz; D_2O): δ 0.4, -0.38, -0.50, -0.9, -1.1.

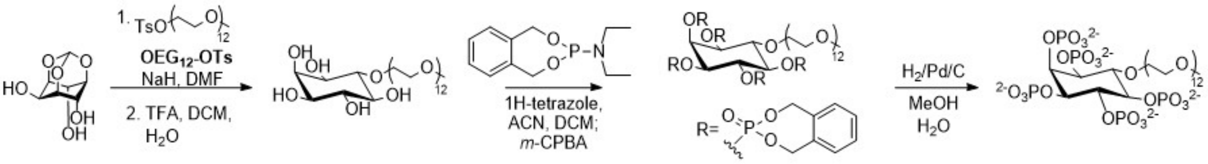

1 (±)-2 (±)-3 4 (±)-OEG$_{12}$-IP5

Supplemental Methods Fig. 1: Synthesis of (±)-OEG$_{12}$-IP5.

3.1.2. Synthesis of 4,6-Di-*O*-(methoxy-diethyleneglycol)-*myo*-inositol-1,2,3,5-tetrakis(phosphate) ((OEG$_2$)$_2$-IP4)

Compound **5** (Supplemental Methods Fig. 2) was prepared as reported previously[2].

Compound **6.** A solution of compound **5** (1.64 mmol, 1.0 eq.) in dry DMF (4 mL) was added dropwise to a solution of sodium hydride (2.1 eq. 60% dispersion in mineral oil) at 0 °C. The resulting mixture was stirred for 30 min at 0 °C, and OEG$_2$-OTs (2.2 eq.) was then added dropwise. Stirring was continued for a further 15 h with warming to RT, after which time TLC (95:5, DCM/methanol) showed the complete conversion of starting material. The reaction mixture was then diluted with

dichloromethane (20 mL), washed with a saturated NH_4Cl solution (10 mL). After separation of the layers, the organic layer was extracted with dichloromethane (2 x 15 mL). The combined organic layers were dried (Na_2SO_4), and evaporated in high vacuo. The resulting compound was purified by column chromatography (DCM/MeOH, 98:2 to 97:3 to 96:4) to afford compound **6,** 2-*O*-*tert*-butyldimethylsilyl 4,6-di-*O*-(2-(2-methoxyethoxy)ethoxy) *myo*-inositol 1,3,5-orthoformate (74%) as yellow oil. ^{1}H-NMR (400 MHz; chloroform-*d*): δ 5.53 (d, *J* = 1.3 Hz, 1H, orthoformate), 4.45 (tt, *J* = 3.4, 1.7 Hz, 1H, Ins), 4.29-4.24 (m, 3H, Ins), 4.15-4.13 (m, 2H, Ins), 3.81-3.59 (m, 12H. OEG), 3.58-3.51 (m, 4H, OEG), 3.39 (s, 6H, OEG-Me), 0.95 (s, 9H, Si-*t*-Bu), 0.15 (s, 6H, Si-Me). ^{13}C-NMR (101 MHz; chloroform-*d*): δ 103.1, 75.2, 73.3, 71.9, 70.7, 69.3, 67.9, 61.6, 59.0, 25.9, 18.4, -4.7

Compound **7.** A solution of compound **6** (0.79 mmol, 1.0 eq.) in methanol (5 mL/mL HCl) and 2 N HCl (15.0 eq.) was stirred at 65 °C for 2 hours. TLC (90:10, DCM/methanol) showed the complete conversion of starting material. The reaction mixture was then neutralized with 1 N sodium hydroxide solution, and evaporated in vacuo. The resulting compound was purified by column chromatography (DCM/MeOH, 92:8 to 90:10 to 85:15 to 80:20) to afford compound **7**, 4,6-Di-*O*-(2-(2-methoxyethoxy)ethoxy) *myo*-inositol (73%) as colorless oil. ^{1}H-NMR (400 MHz; D_2O): δ 3.96 (t, *J* = 2.7 Hz, 1H, H2), 3.93-3.82 (m, 4H, OEG), 3.69-3.60 (m, 8H, OEG), 3.58-3.54 (m, 4H, OEG), 3.53-3.47 (m, 2H, H1/3), 3.47-3.34 (m, 3H, H4/6 + H5), 3.30 (s, 6H, MeO). ^{13}C-NMR (101 MHz; D_2O): δ 81.8, 73.5, 72.0, 71.4, 71.0, 70.5, 70.1, 69.3, 58.0.

Compound **8.** Protocols are based on Godage et al.[3] To a solution of compound 7 (0.49 mmol, 1.0 eq.) and 5-phenyltetrazole (12-16 eq.) in dry dichloromethane (5 mL/mmol) under an atmosphere of argon was added dibenzyl *N*,*N*-diisopropylphosphoramidite (6-8 eq.). Stirring was continued for 1-3 days at RT, after which time TLC (90:10, DCM/methanol) confirmed the complete consumption of starting material. The reaction mixture was cooled to 0 °C and 30% H_2O_2 (12-16 eq.) was added dropwise while stirring. The cooling bath was removed, and the mixture was allowed to reach room temperature. After 45 min, TLC (90:10, DCM/methanol) showed complete oxidation of pentaphosphite to pentaphosphate, the reaction mixture was diluted with dichloromethane (20 mL), washed with 10% sodium sulfite solution (2 x 15 mL), dried (Na_2SO_4), and solvent was evaporated in vacuo. The residue was purified by column chromatography (dichloromethane/methanol, 99:1 to 98:2

to 97:3 to 96:4, to 95:5 to 94:6) to afford compound **8**, 4,6-di-*O*-(2-(2-Methoxyethoxy)ethoxy) 1,2,3,5-tetrakis-*O*-[bis(benzyloxy)phosphoryl]-*myo*-inositol (44%) as a colorless oil. ^{1}H-NMR (400 MHz; chloroform-*d*): .43-7.21 (m, 40H, Ph), 5.38 (dt, J = 8.4, 2.0 Hz, 1H, H2), 5.17-5.02 (m, 16H, PhCH2), 4.28-4.23 (m, 3H, H1/3 + H5), 3.84-3.77 (m, 6H, OEG + H4/6), 3.52-3.47 (m, 4H, OEG), 3.41-3.37 (m, 4H, OEG), 3.34-3.29 (m, 4H, OEG), 3.23 (s, 6H, OCH3). ^{31}P-NMR (162 MHz; chloroform-*d*): δ -1.5, -1.86, -1.90

Compound **9** (**(OEG_2)$_2$-IP4**, Supplemental Methods Fig. 2). Compound **8** (0.48 mmol, 1.0 eq.) was dissolved in methanol (10 mL), and water (3 mL) and 10% palladium on activated charcoal (0.4 – 0.5 eq.) was added. The resulting suspension was stirred at RT overnight under an atmosphere of hydrogen (balloon). The catalyst was filtered through a GF/A glass microfiber filters paper, washed with methanol (3 x 3 mL), water (3 x 2 mL) and 1 N sodium hydroxide solution (3 x 2 mL). The pH was adjusted to 8 with 2 N HCl, and the filtrate was evaporated under reduced pressure. The residue was purified by dissolving in a minimal amount of water (2-3 mL), filtering through a GF/A glass microfiber filters paper, and size-exclusion chromatography (30g sephadex G-15), eluting with water. Fractions containing product were detected by Ceric Ammonium Molybdate stain (CAM) and $KMnO_4$ stain on thin-layer chromatography, and lyophilized. Additional purification was performed using RP18 silica gel (30 g) eluting with water and yielding compound **9**, 4,6-Di-*O*-(methoxy-diethyleneglycol)-*myo*-inositol-1,2,3,5-tetrakis(phosphate) (70%) as white solid. ^{1}H-NMR (400 MHz; D_2O): δ 4.84-4.81 (m, 1H, H2), 4.06-3.91 (m, 5H, H1/3 + H5 + 2 OEG), 3.88-3.83 (m, 2H, OEG), 3.70-3.63 (m, 10H, H4/6 + 8 OEG), 3.58-3.50 (m, 4H, OEG), 3.30 (s, 6H, MeO). ^{31}P-NMR (162 MHz; D_2O): δ 0.4, -0.25, -1.16.

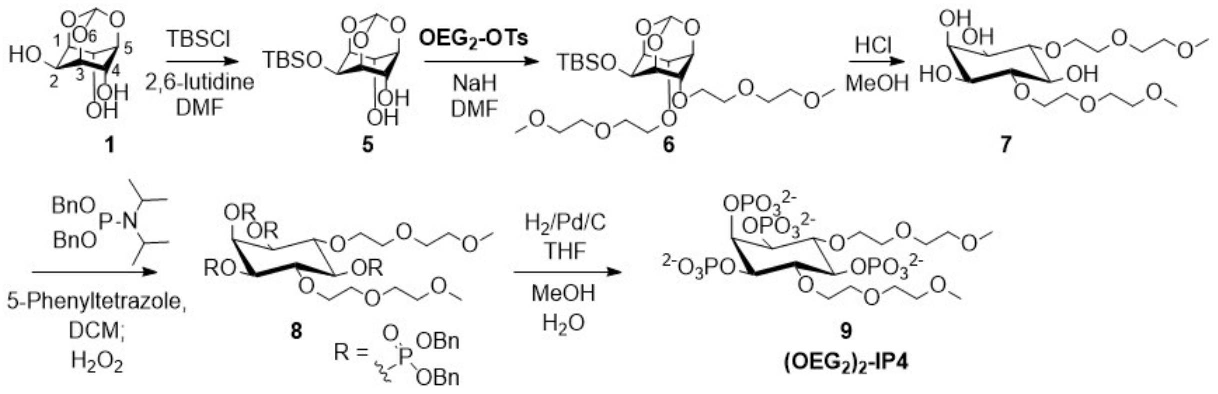

Supplemental Methods Fig. 2: Synthesis of $(OEG_2)_2$-IP4. Compounds are presented in the thermodynamically more stable five-equatorial, one-axial conformation.

3.1.3. Synthesis of 2,4,6-tri-*O*-(methoxy-heptaethyleneglycol)-*myo*-inositol-1,3,5-tris(phosphate) (($(OEG_7)_3$-IP3)

Compound **10** (Supplemental Methods Fig. 3). 1,3,5-*O*-methylidyne-*myo*-inositol (34 mg, 0.18 mmol, 1 eq.) and OEG_7-OTs (400 mg, 0.81 mmol, 4.5 eq.) were dissolved in DMF (2 mL) and dried over molecular sieves overnight. NaH (0.81 mmol, 4.5 eq.) was added to dry DMF (1 mL). The solution was then added to the sodium hydride suspension and stirred for 48 h, after which it was quenched with water and extracted with DCM. The organic layer was washed with brine and dried over Na_2SO_4. The crude product was purified by silica gel column chromatography using a gradient of 0-7% MeOH/DCM, yielding 134 mg (64%). ^{1}H-NMR (500 MHz, chloroform-*d*) δ 5.50 (d, *J* = 1.3 Hz, 1H), 4.51 – 4.48 (m, 1H), 4.37 – 4.34 (m, 2H), 4.29 (t, *J* = 3.7 Hz, 2H), 3.85 – 3.83 (m, 1H), 3.68 – 3.63 (m, 84H), 3.40 (s, 9H). ^{13}C-NMR (126 MHz, chloroform-*d*) δ 103.12, 74.91 (2C), 70.86 – 70.34 (m), 69.19 (2C), 68.96, 68.67, 59.05 (3C).

Compound **11.** Compound **10** (40 mg, 0.0346 mmol) was dissolved in DCM (0.2 mL) and TFA (2 mL) was dissolved in water (0.2 mL). The TFA solution was slowly added to the reaction mixture and allowed to react for 1 h at RT. The mixture was co-evaporated with toluene under vacuum and purified by silica gel chromatography using a gradient of 0-10% MeOH/DCM, yielding 32 mg (81%). ^{1}H-NMR (400 MHz; D_2O): δ 3.92-3.82 (m, 6H), 3.78 (t, *J* = 2.8 Hz, 1H), 3.68-3.58 (m, 74H), 3.55-3.50 (m, 8H), 3.43 (t, *J* = 9.6 Hz, 2H), 3.35-3.30 (m, 1H), 3.30 (s, 9H).

Compound **12**. Compound **11** (32 mg, 0.0282 mmol, 1 eq.) was dissolved in anhydrous DCM (1 mL) and tetrazole (0.423 mmol, 15 eq.) was added. The reaction mixture was dried over molecular sieves overnight. *o*XEP (0.2115 mmol, 7.5 eq.) was added and allowed to react overnight. The reaction mixture was cooled to -10 °C. *m*CPBA (0.846 mmol, 30 eq.) was dissolved in DCM and dried over Na_2SO_4 for 30 min. The *m*CPBA mixture was added to the cooled reaction mixture and allowed to react at -10 °C for 20 min. The reaction was warmed to RT and allowed to react for 40 min, then filtered and Na_2SO_3 (1 M) was added to the filtrate. The crude product was extracted with DCM and washed with water, saturated $NaHCO_3$ and brine, dried over $Na_2S_2O_3$, and chromatographed over silica gel using a gradient of 0-10% MeOH/DCM. ^{1}H-NMR (500 MHz, chloroform-*d*) δ 7.41 – 7.29 (m, 12H), 5.45 – 5.29 (m, 6H), 5.23 – 5.11 (m, 6H), 4.52 (t, *J* = 2.6 Hz, 1H), 4.42 (q, *J* = 9.2 Hz, 1H), 4.27 (ddd, *J* = 9.9, 7.3, 2.5 Hz, 2H), 4.02 (dd, *J* = 5.8, 4.0 Hz, 2H), 3.94 – 3.40 (m, 84H), 3.37 (s, 9H). ^{13}C-NMR (126 MHz, chloroform-*d*) δ 136.77 – 127.97 (m), 71.94, 70.81 – 70.03 (m), 59.05.

Compound **13** (**(OEG$_7$)$_3$-IP$_3$**). Compound **12** (65 mg, 0.039 mmol) was dissolved in 15 mL MeOH/water (3:2 *v/v*) and Pd/C was added. The reaction was purged with H_2, and allowed to react under H_2 (1 atm) for 48 h. The reaction was subsequently purged with argon, neutralized with a few drops of triethylamine, and filtered over celite, which was washed with water, MeOH, water, and aqueous NH_4OH. The crude product was concentrated under vacuum, and purified with size exclusion chromatography (GE Healthcare, PD-10 Columns, 8.3 mL Sephadex, G-25 resin). The sodium salt was prepared by eluting an aqueous solution through a column packed with Dowex 50WX8 Na form, yielding 15.8 mg. ^{1}H-NMR (500 MHz; D_2O): δ 4.21-4.19 (m, 1H), 4.01-3.87 (m, 8H), 3.87-3.79 (m, 3H), 3.75-3.52 (m, 90H), 3.31 (s, 9H). ^{31}P-NMR (203 MHz; D_2O): δ 0.4, -1.2.

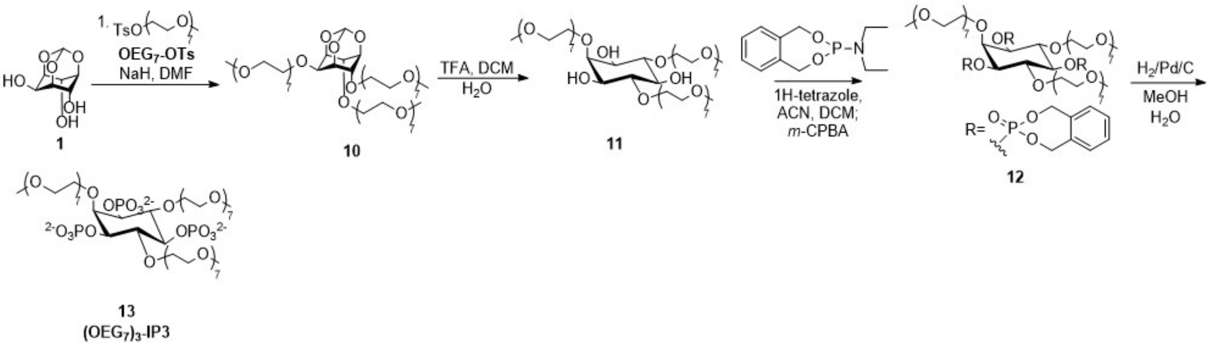

Supplemental Methods Fig. 3: Synthesis of (OEG$_7$)$_3$-IP3.

3.2. Off-target screening assay

The experiments were conducted by a contract research organization (Eurofins Panlabs Discovery Services Taiwan, Ltd.) and details on the respective assays can be retrieved via the catalog number provided in Supplementary Table 11 from the company's webpage (https://www.eurofinsdiscoveryservices.com/).

3.3. Protein binding of $(OEG_2)_2$-IP4 in human plasma

Healthy human plasma (EDTAK3 plasma, Research Laboratory of the Service of Nephrology, CHUV, Lausanne, Switzerland) were spiked (5% *v/v*) using dedicated dilutions of $(OEG_2)_2$-IP4 in water at 3 and 30 µg/mL and incubated during 20 min at 37 °C to allow protein binding. Then, each sample were split into two aliquots and one aliquot underwent ultrafiltration using instrumentation and method described previously[4], whereas the other aliquote was used for total concentrations measurements. Ultrafiltrated spiked plasma aliquots of 50 µL were processed with 150 µL MeOH including OEG_{11}-IP5 as internal standard (final concentration 5000 ng/mL). Concentrations of unbound $(OEG_2)_2$-IP4 in ultrafiltrated fractions and total $(OEG_2)_2$-IP4 concentrations were measured using a calibration prepared in ultrafiltrated pooled plasma and non-ultrafiltrated pooled plasma, respectively and LC-MS/MS quantification as described in Methods 6.3.

3.4. Bone analysis

At sacrifice the left tibia of animals of the rat model with dietary adenine/high-phosphate-induced uremia and VC was collected, fixed with 70% *v/v* EtOH and embedded in methyl methacrylate. The trabecular bone compartment is known to be the most metabolic active i.e. actively re-structured bone compartment in CKD. Therefore, histomorphometric and micro-computed tomography (µCT) analysis of bone was performed on the trabecular compartment. Tibial sections were Goldner-stained for histomorphometric analysis of the proximal metaphysis using the AxioVision Release 4.5 software, as described previously[5]. The total bone area (as percentage of total tissue area), osteoid area (as percentage of total bone area), and mineralized bone area (as percentage of total tissue area) were calculated from the primary measurements. Images of osteoclast and osteoblast were taken (Leica

DMR microscope, 200x magnification). In order to perform 3D analysis of mineralized bone structure, *ex vivo* µCT analyses of the methylmethacrylate embedded left tibia was performed using a high-resolution µCT scanner (Skyscan 1076, SkyScan, Belgium) also at the proximal metaphysis. The X-ray source was operated at 80 kV and 110 µA and a 25µm titanium filter was used. Images were captured using 10 megapixel CCD-camera.

Samples were scanned at a magnification resulting in a voxel size of 9 µm. Cross-sections along the specimen's long-axis were reconstructed using NRecon (v1.7.4.6; SkyScan). Ctan (v1.18.8.0; Skyscan) was used to analyze and to perform 3D rendering, respectively and to generate the pseudocolor images. Calibration of BMD was performed by scanning appropriate phantoms using identical X-ray settings.

In order to be able to compare the bone analysis results with that of control (non-CKD) rats, bone of control rats (n = 5) of the same age and genetic background as used for the current study was also analyzed.

4. Appendix NMR Spectra

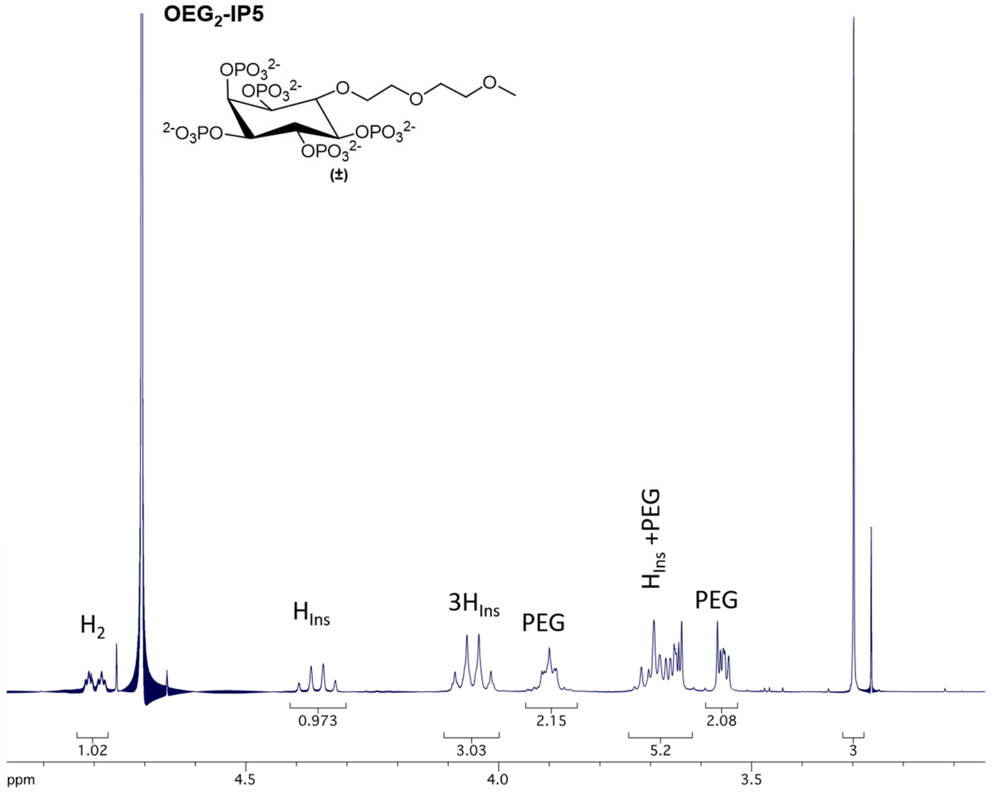

^{1}H NMR spectrum of OEG$_2$-IP5

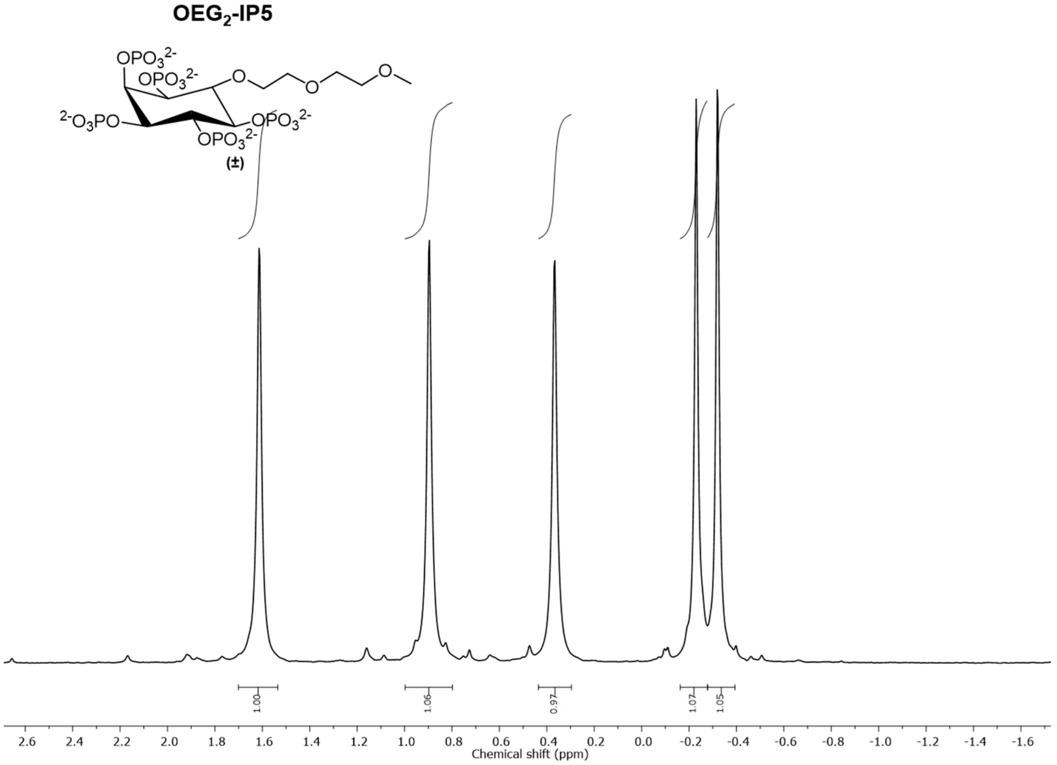

^{31}P NMR spectrum of OEG$_2$-IP5

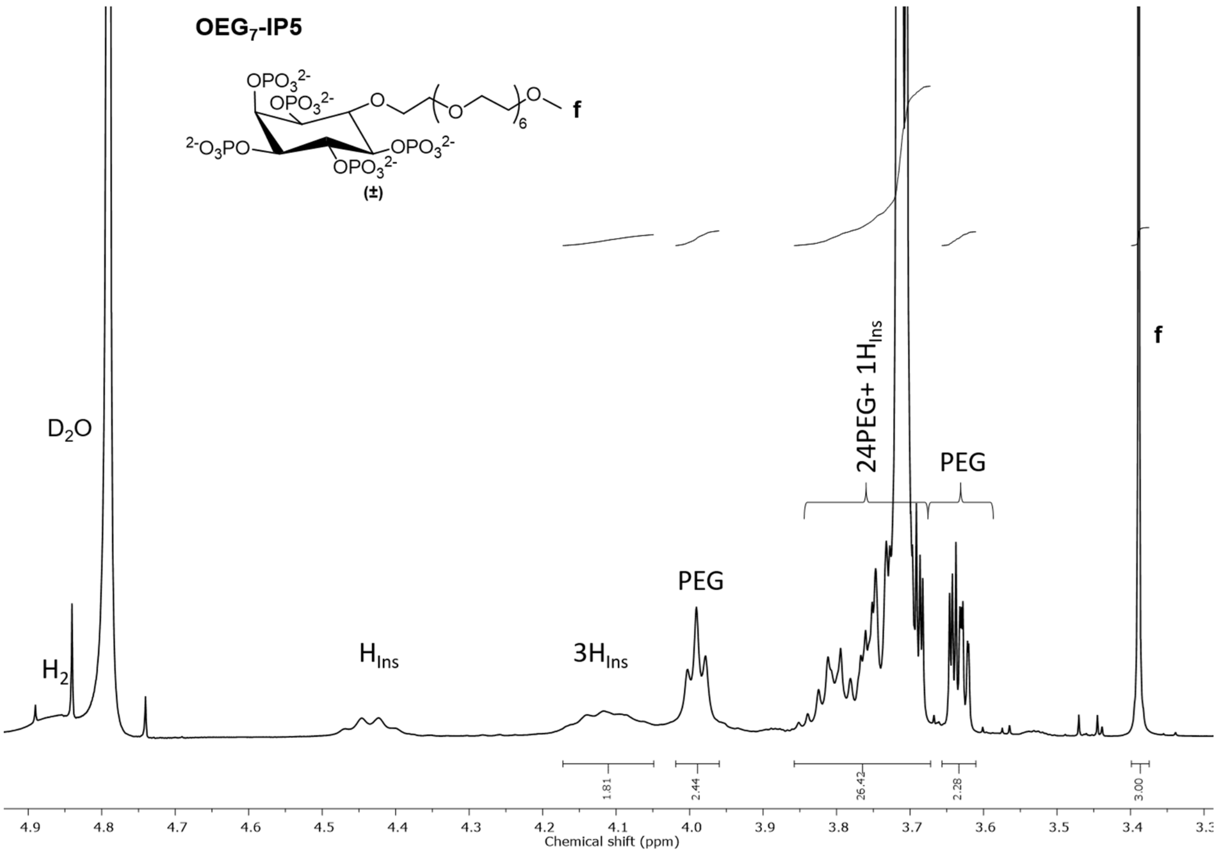

^{1}H NMR spectrum of OEG$_7$-IP5

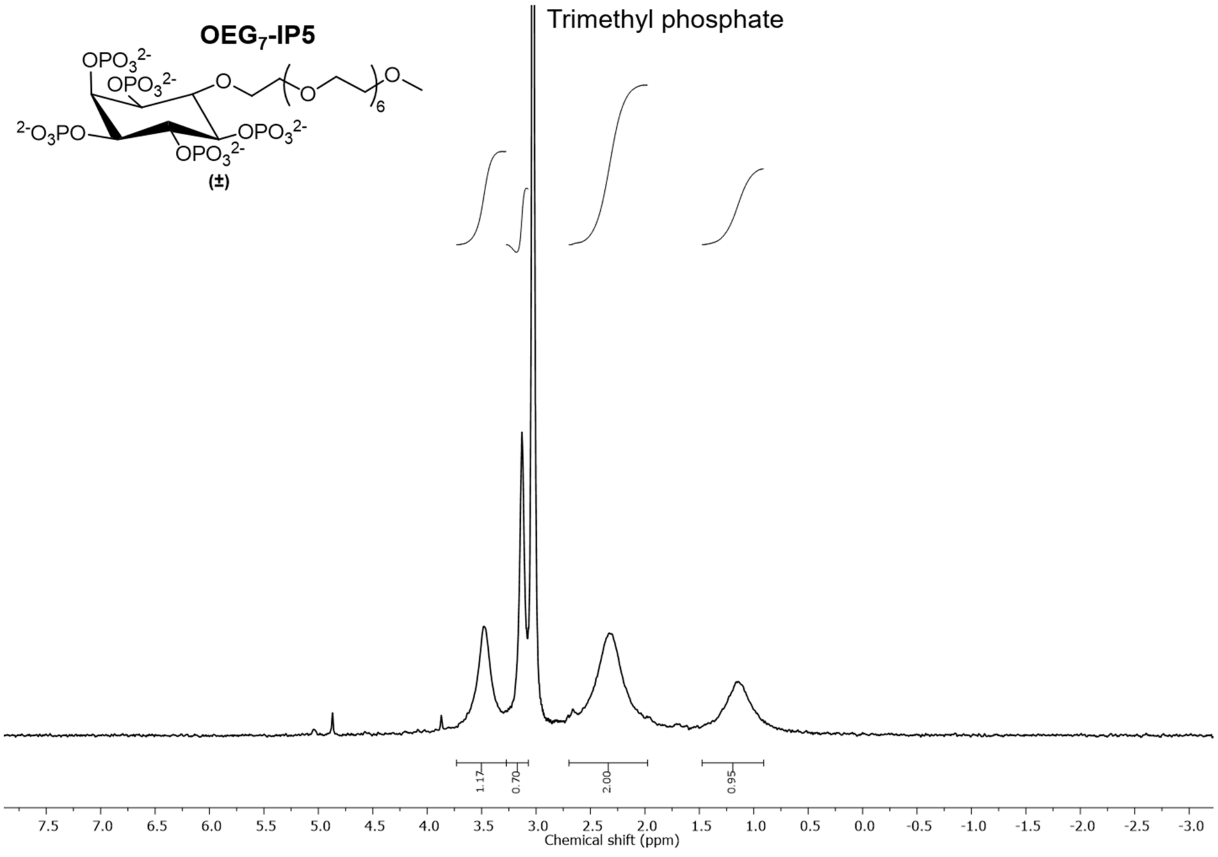

^{31}P NMR spectrum of OEG$_7$-IP5

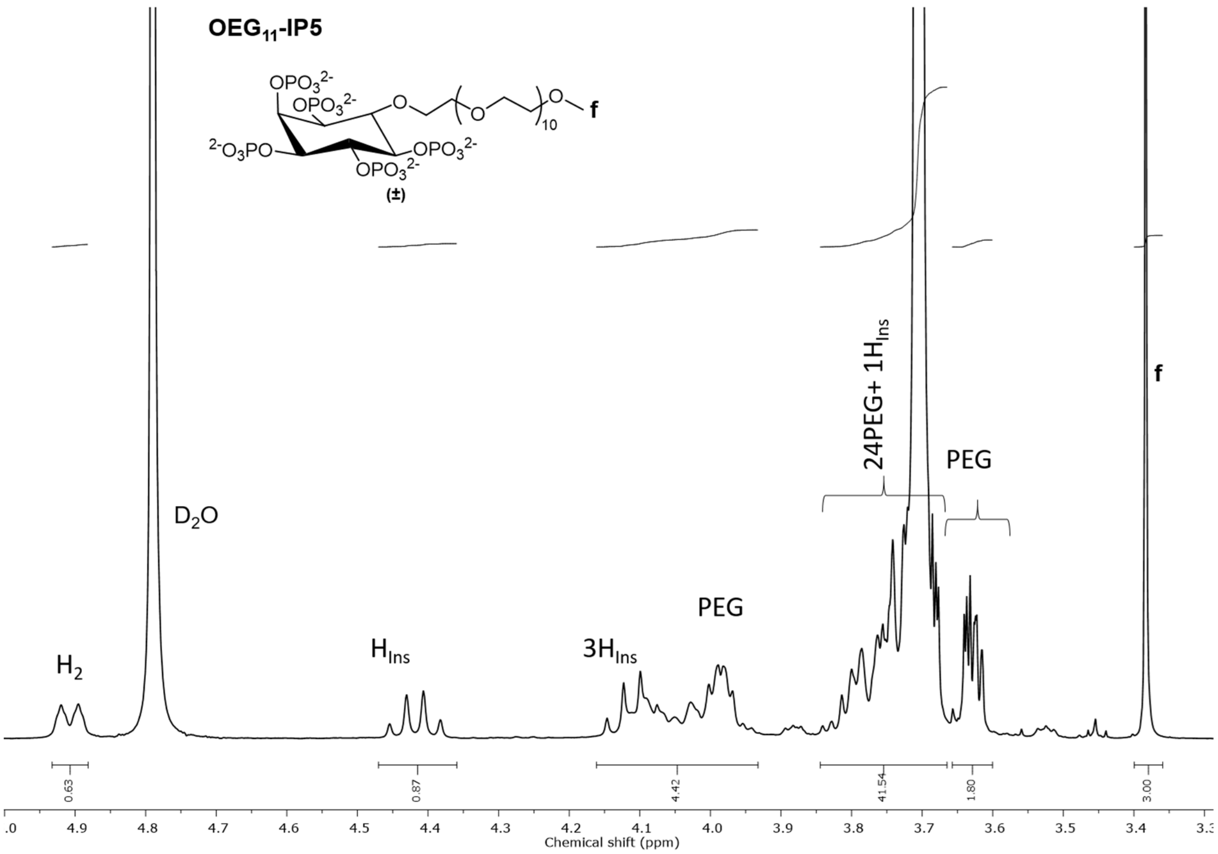

^{1}H NMR spectrum of OEG$_{11}$-IP5

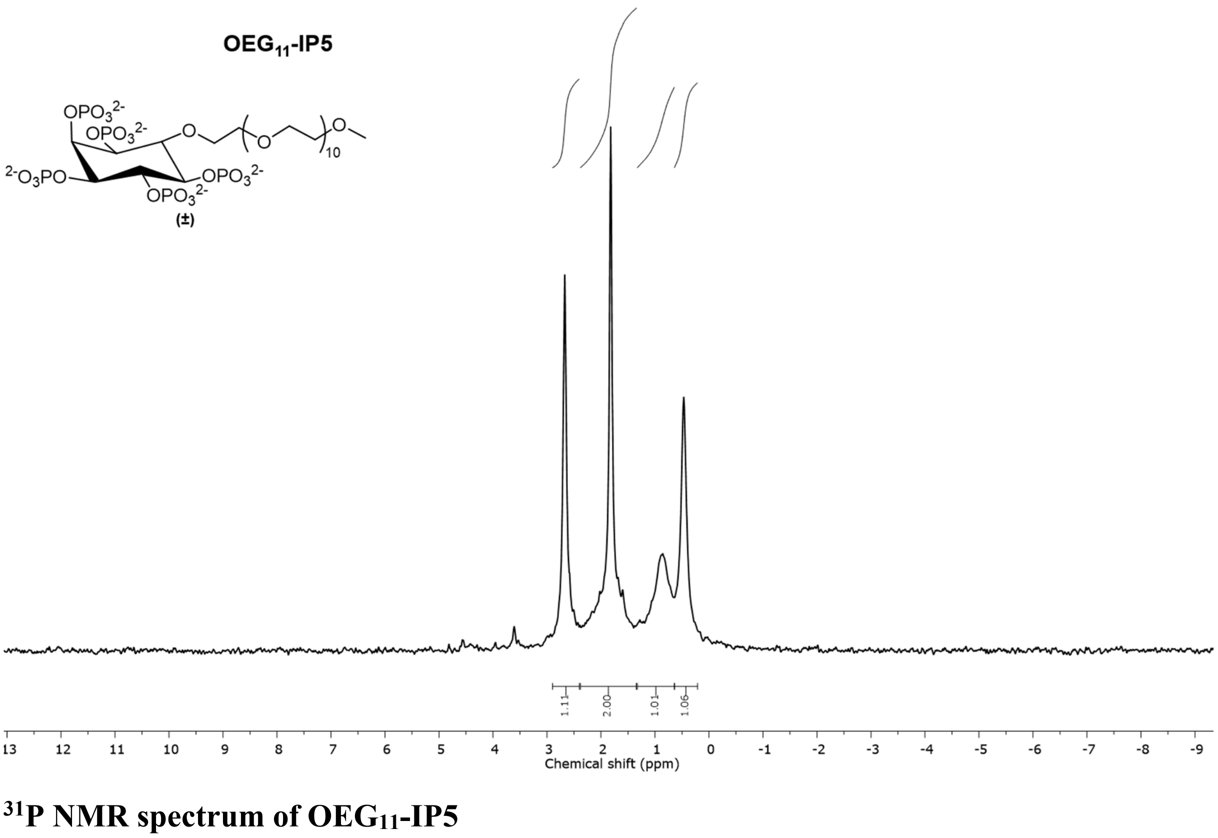

^{31}P NMR spectrum of OEG$_{11}$-IP5

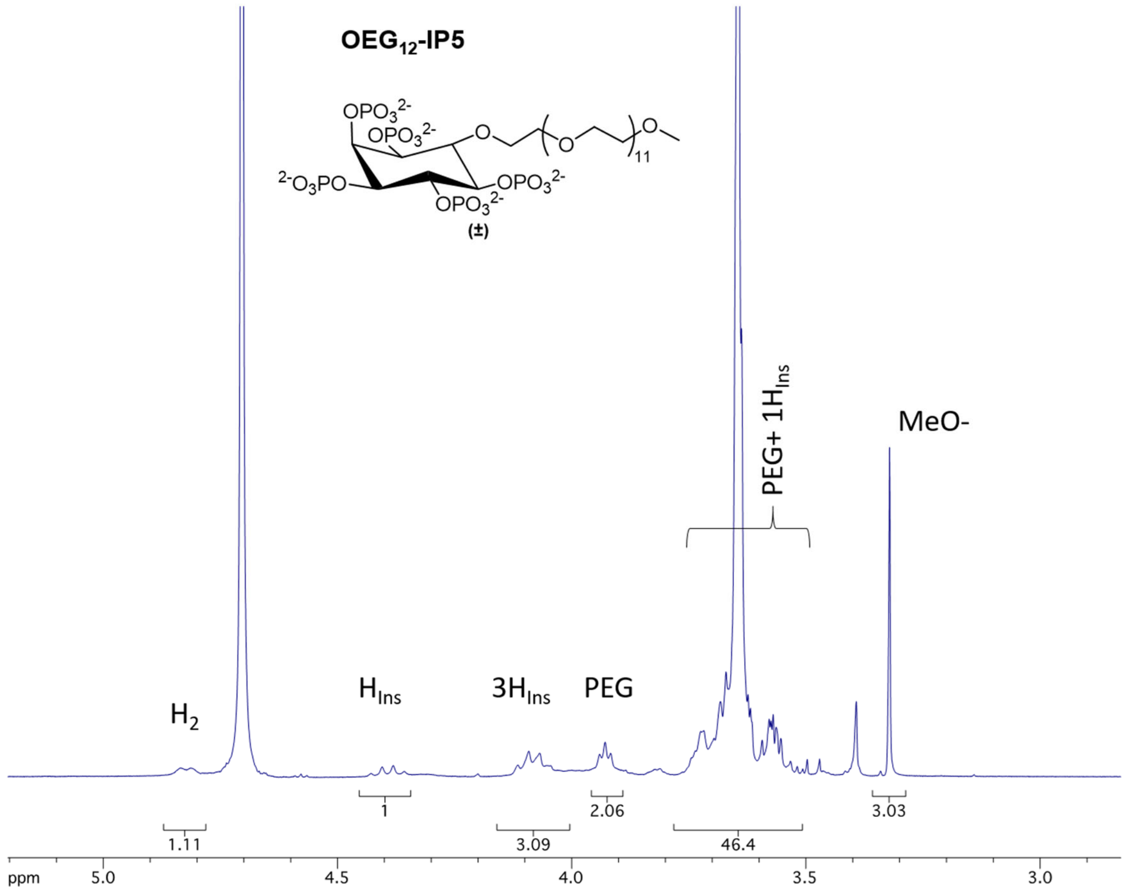

^{1}H NMR spectrum of OEG_{12}-IP5

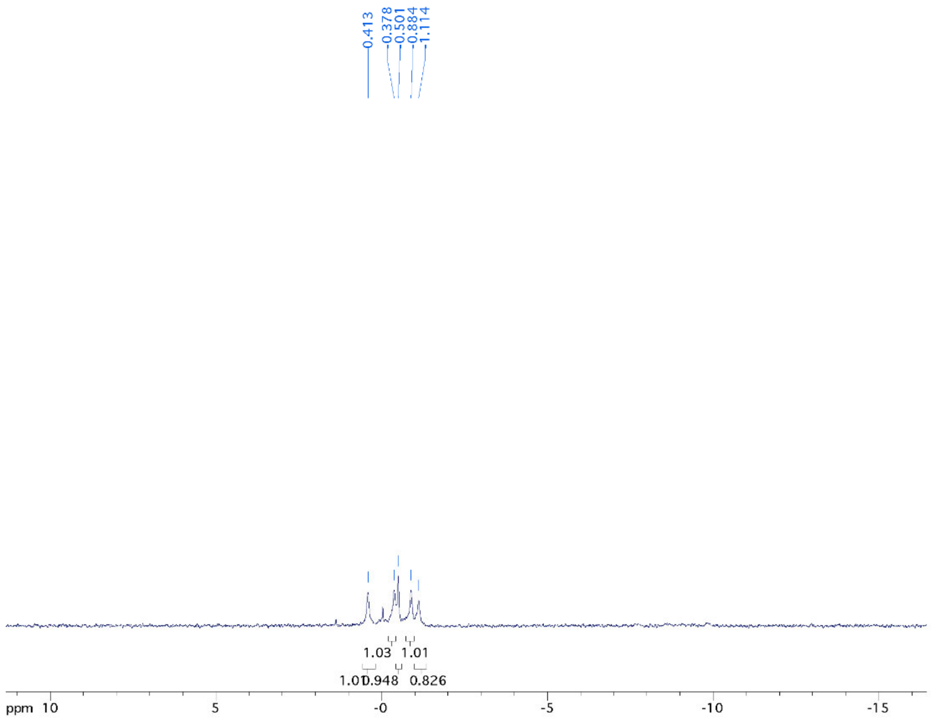

^{31}P NMR spectrum of OEG_{12}-IP5

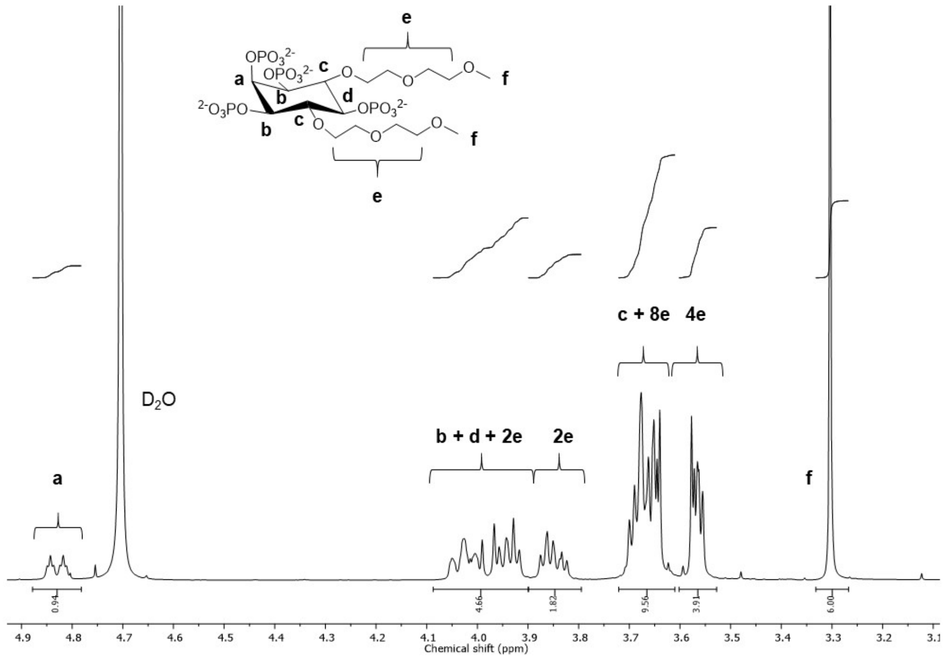

^{1}H NMR spectrum of $(OEG_2)_2$-IP4

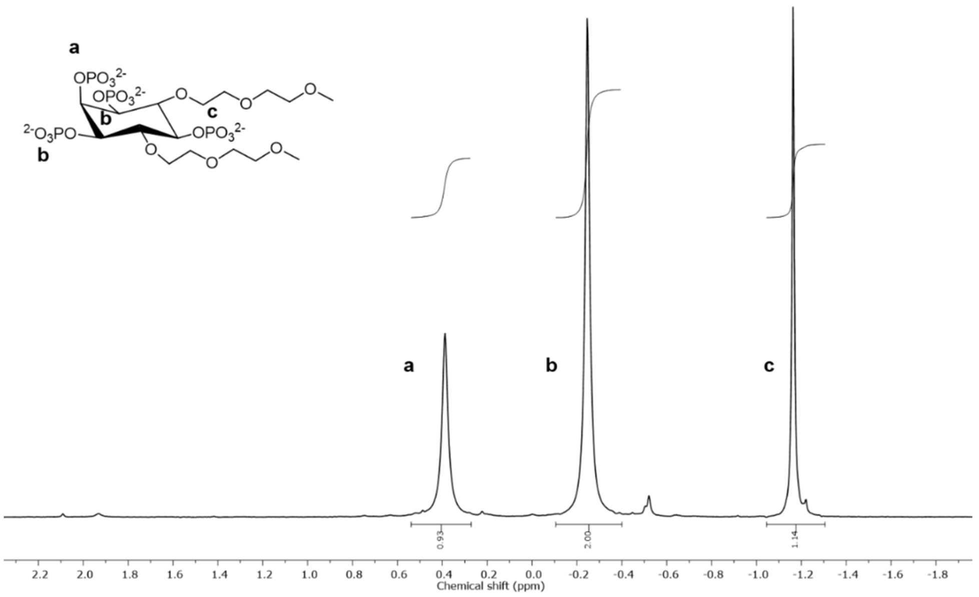

^{31}P NMR spectrum of $(OEG_2)_2$-IP4

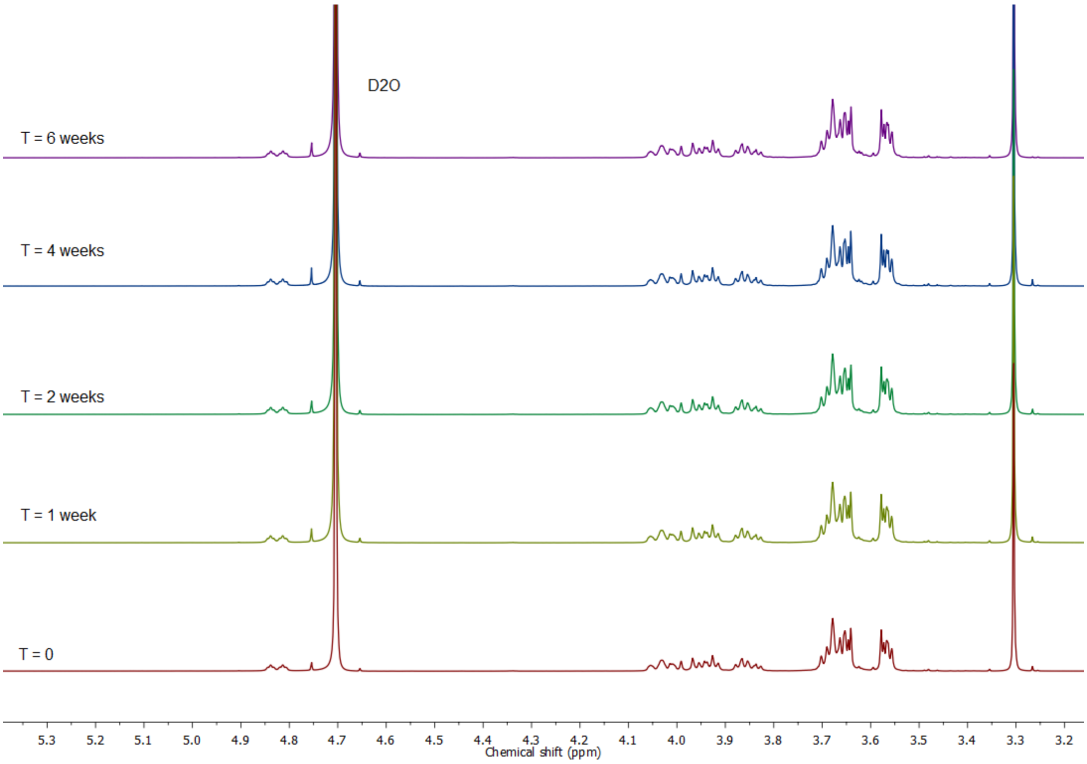

Monitoring the stability of $(OEG_2)_2$-IP4 in aqueous solution at 37 °C for up to 6 weeks by ^{1}H-NMR spectroscopy. No compound degradation was evident.

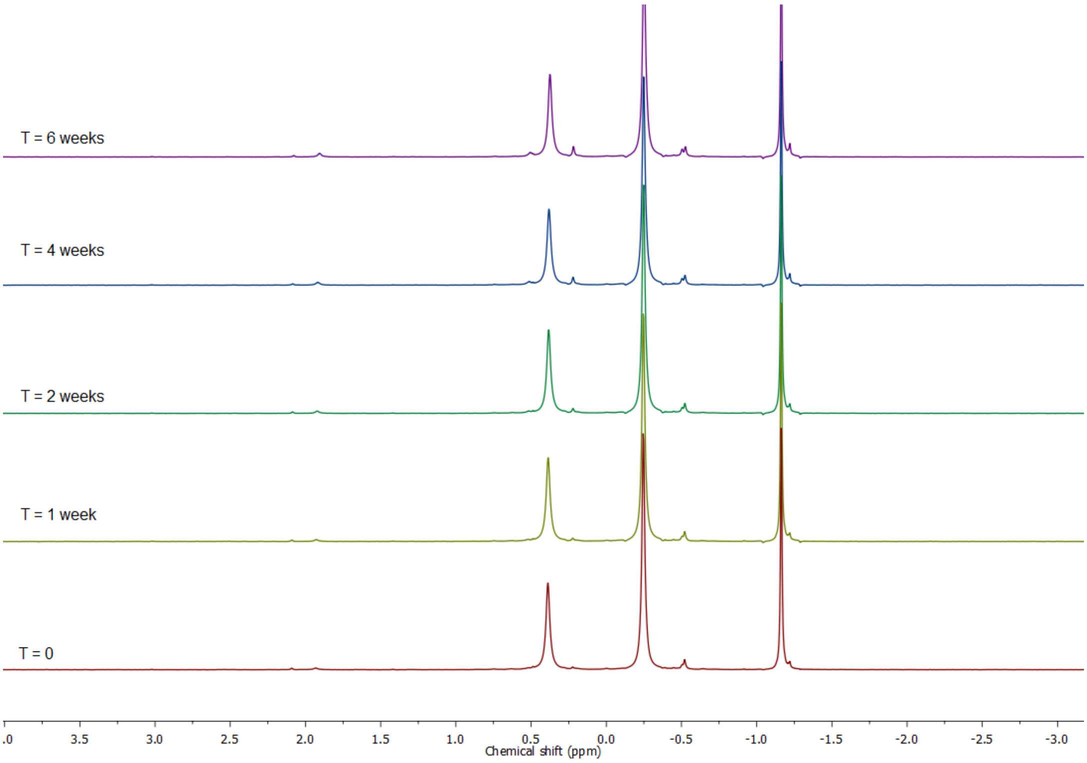

Monitoring the stability of $(OEG_2)_2$-IP4 in aqueous solution at 37 °C for up to 6 weeks by ^{31}P-NMR spectroscopy. No compound degradation was evident.

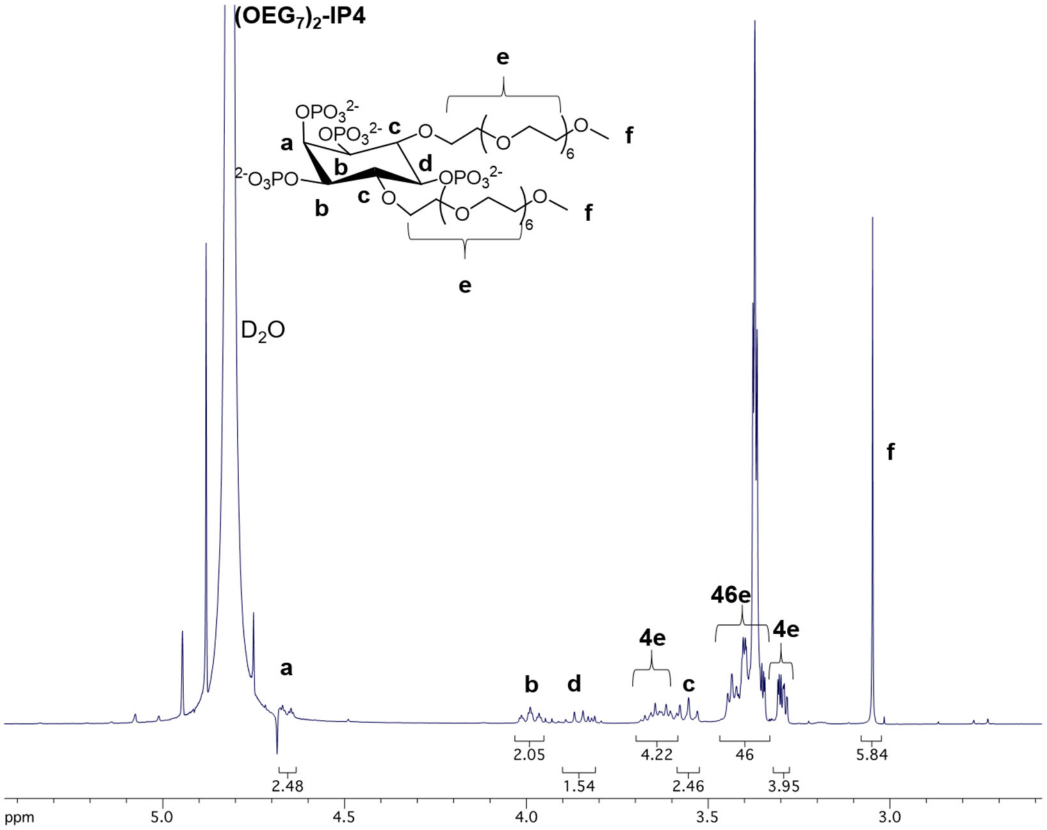

^{1}H NMR spectrum of (OEG_7)$_2$-IP4

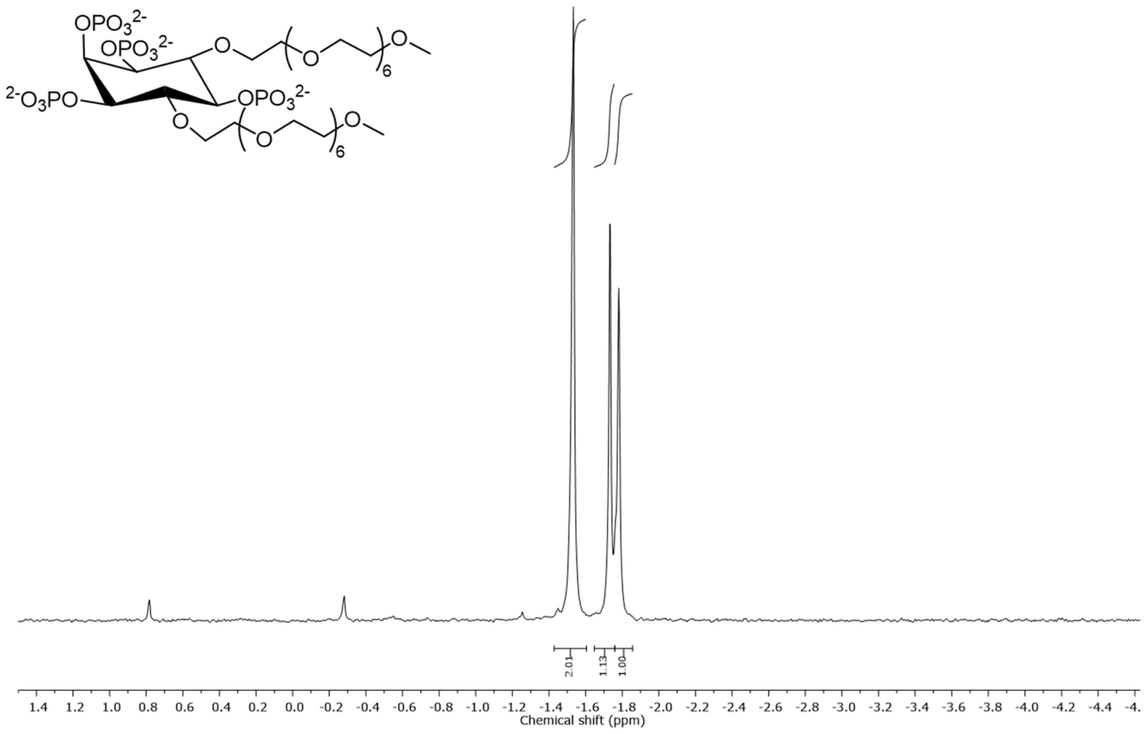

^{31}P NMR spectrum of (OEG_7)$_2$-IP4

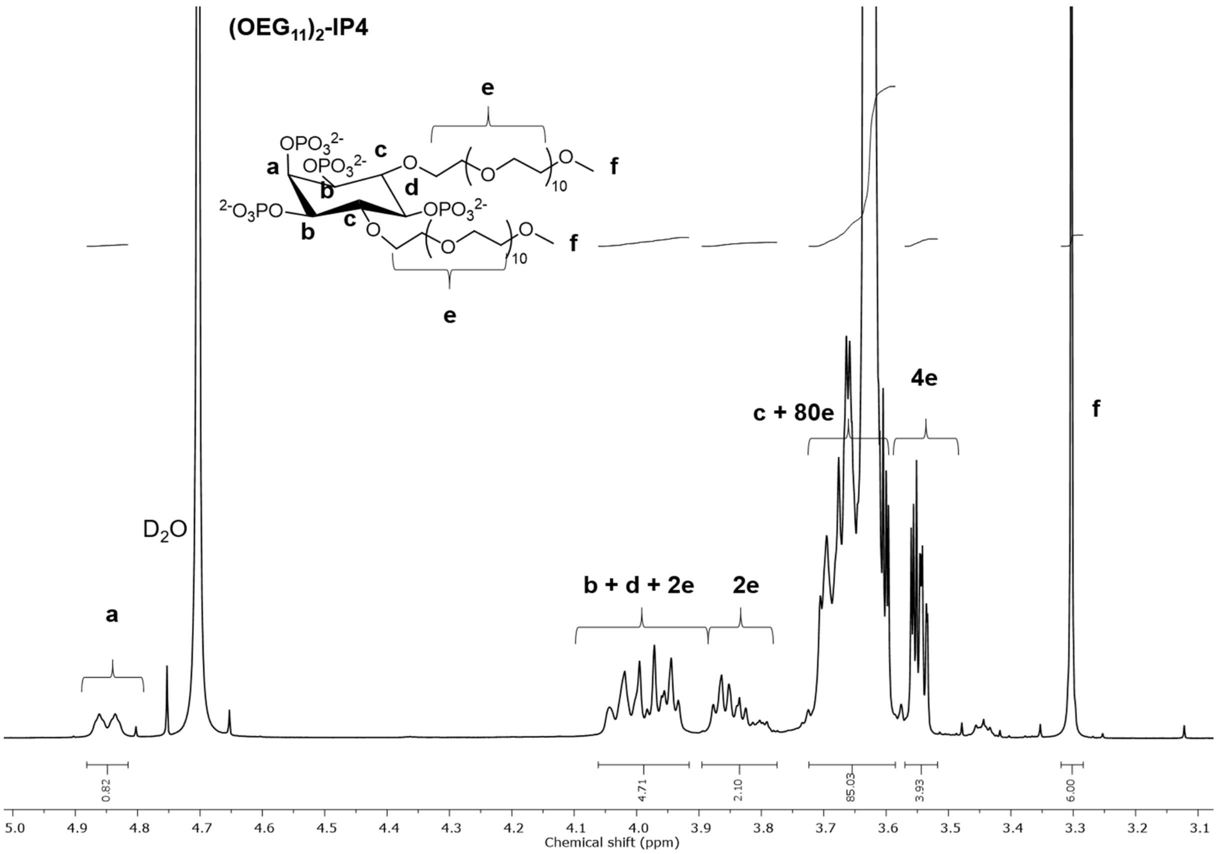

^{1}H NMR spectrum of $(OEG_{11})_2$-IP4

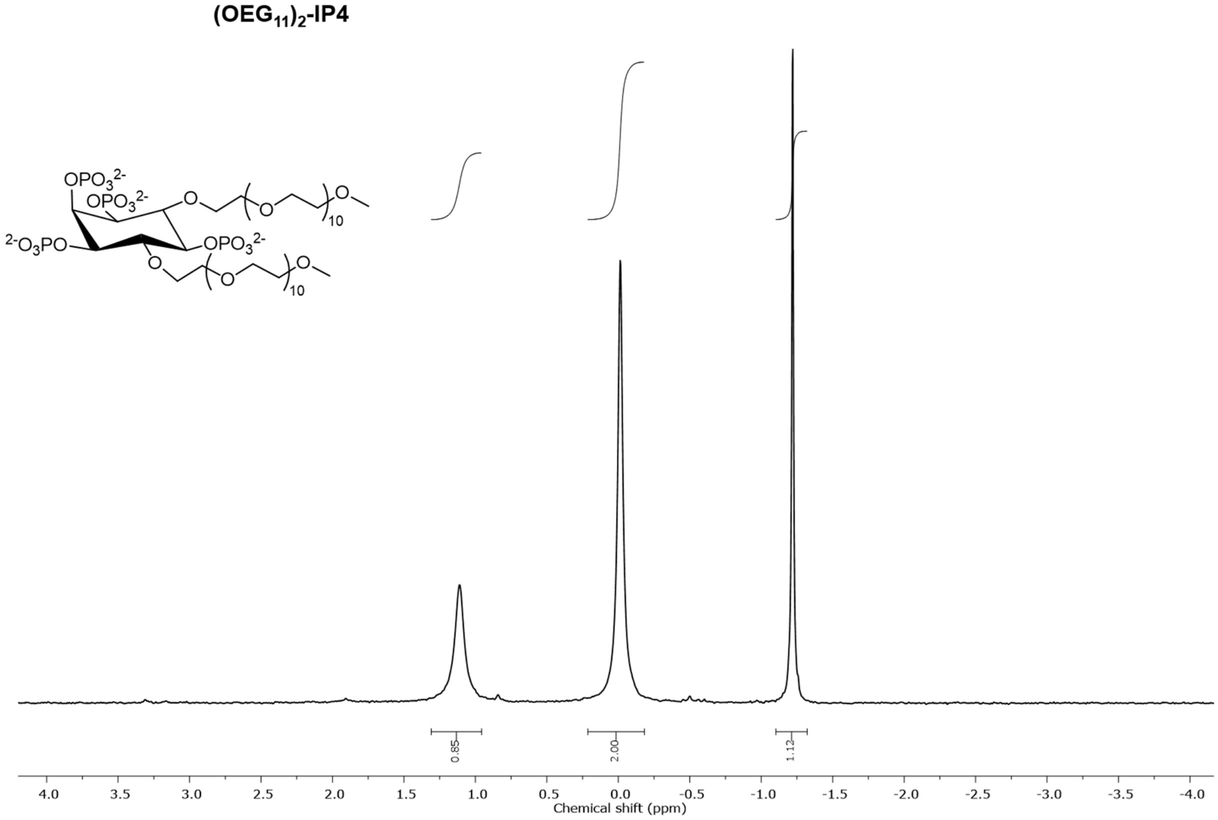

^{31}P NMR spectrum of $(OEG_{11})_2$-IP4

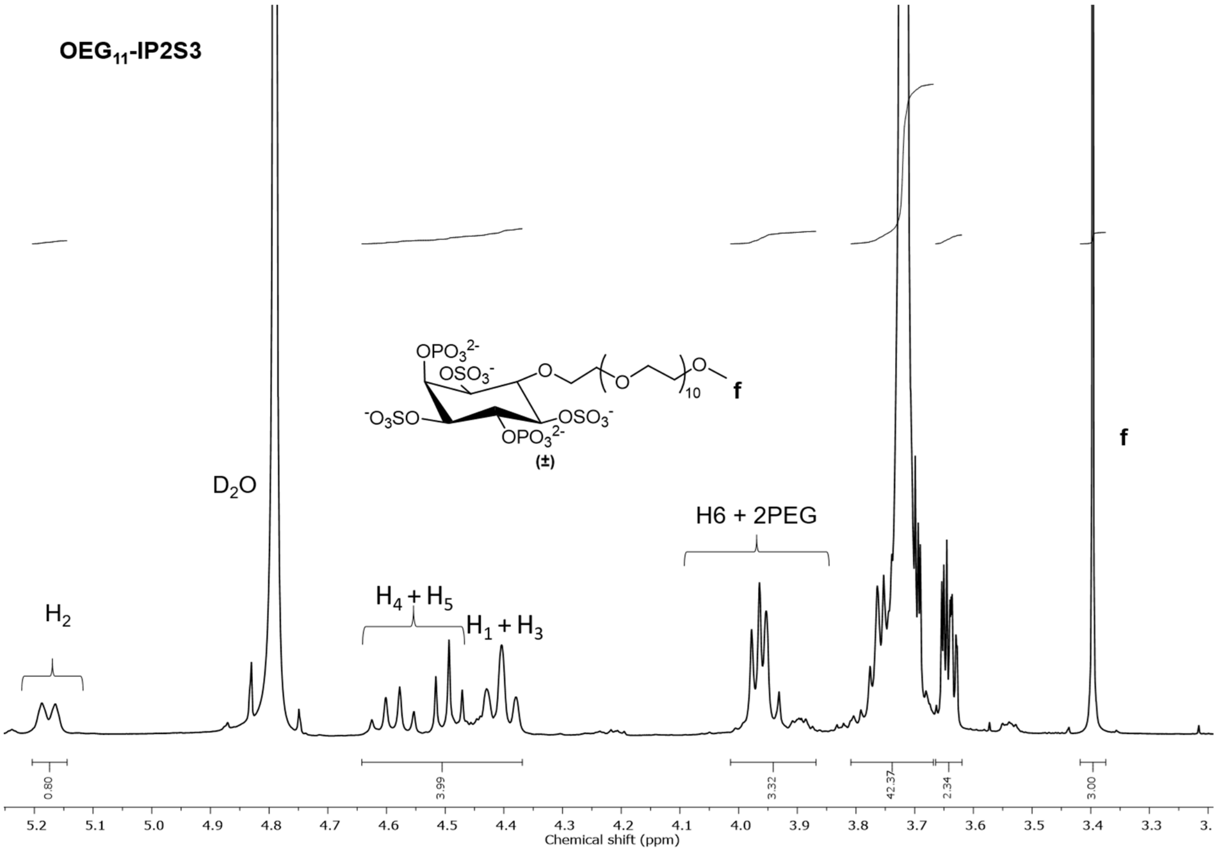

^{1}H NMR spectrum of OEG_{11}-IP2S3

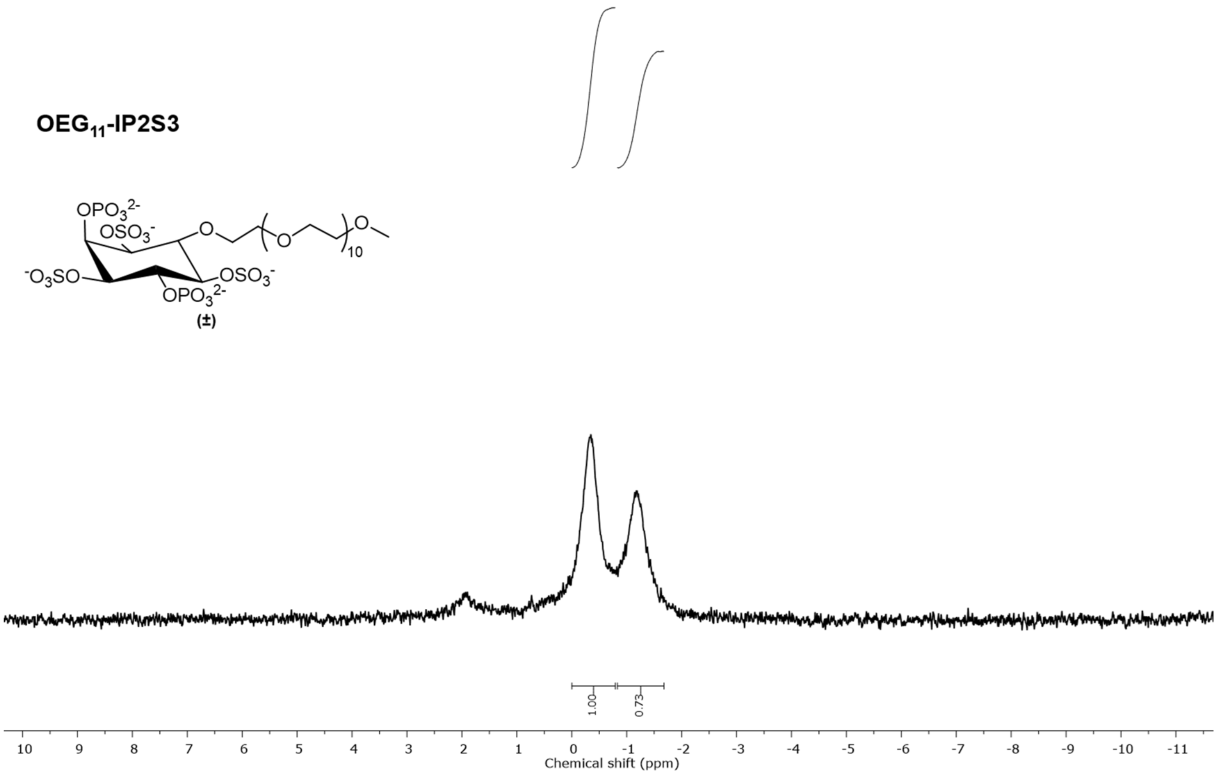

^{31}P NMR spectrum of OEG_{11}-IP2S3

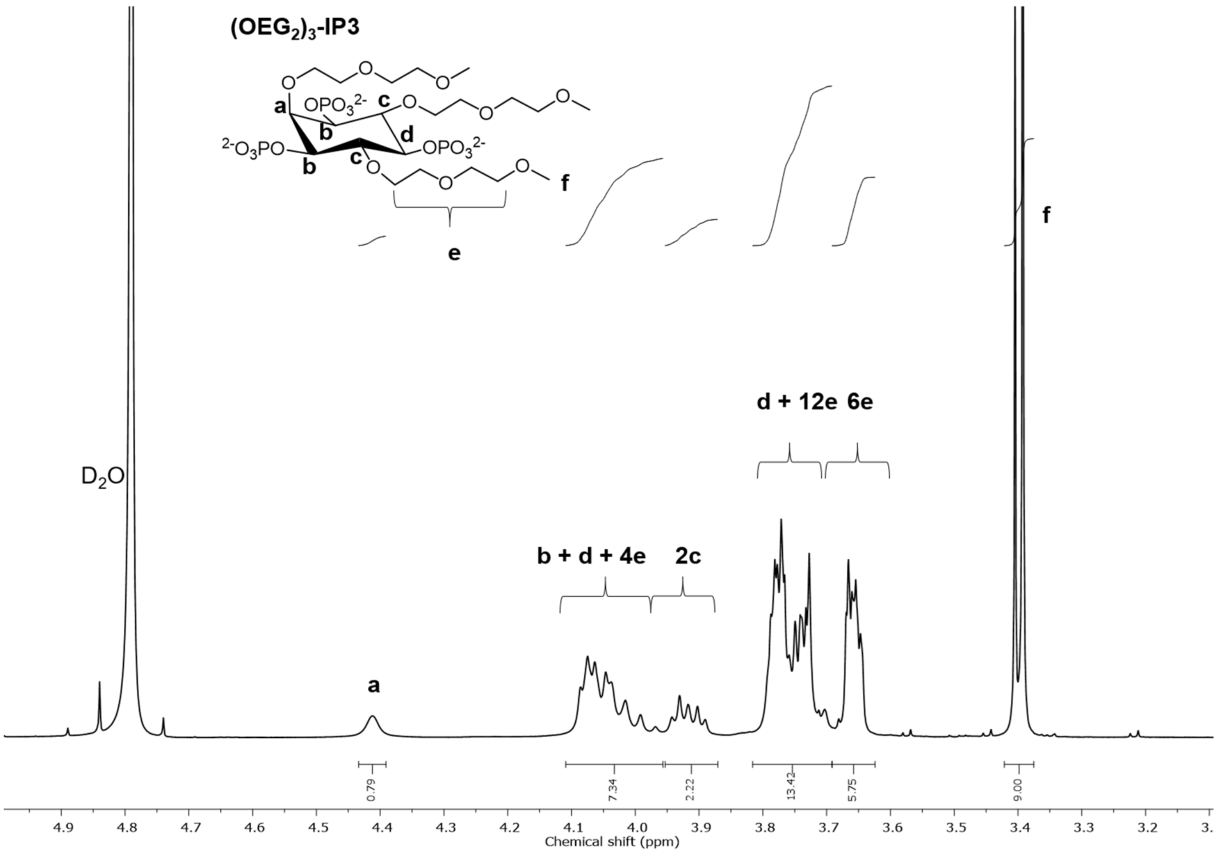

^{1}H NMR spectrum of $(OEG_2)_3$-IP3

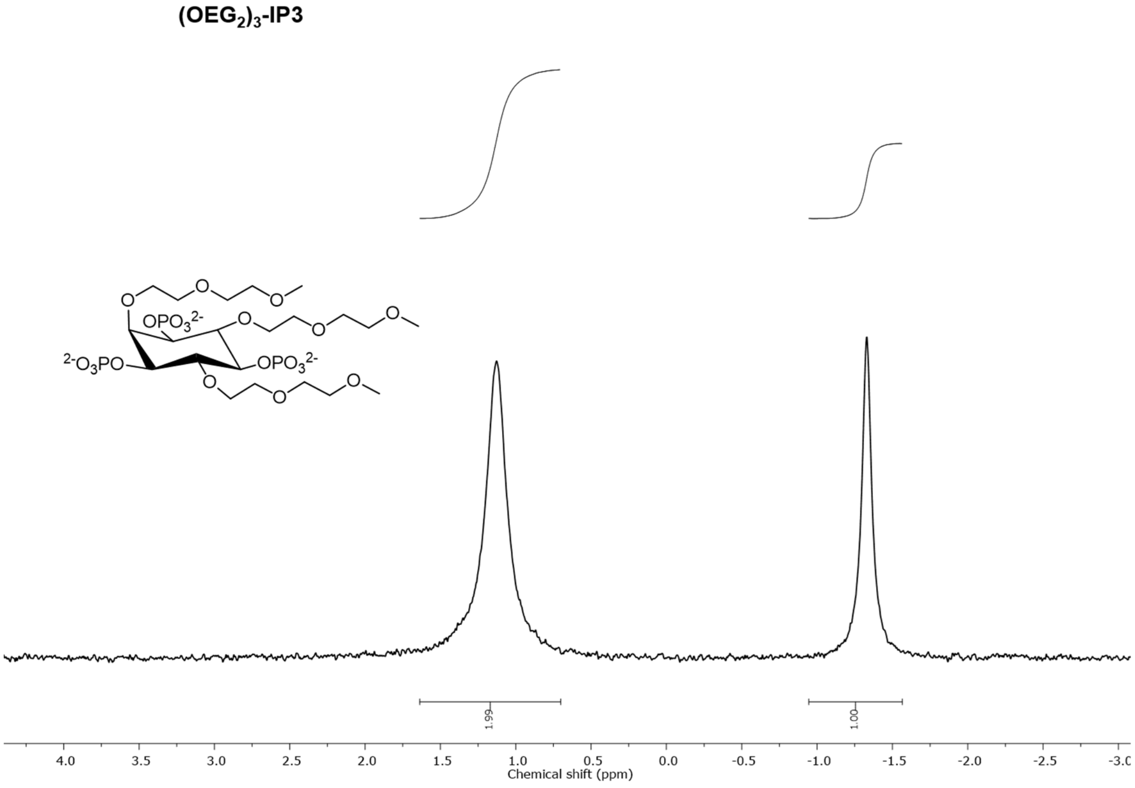

^{31}P NMR spectrum of $(OEG_2)_3$-IP3

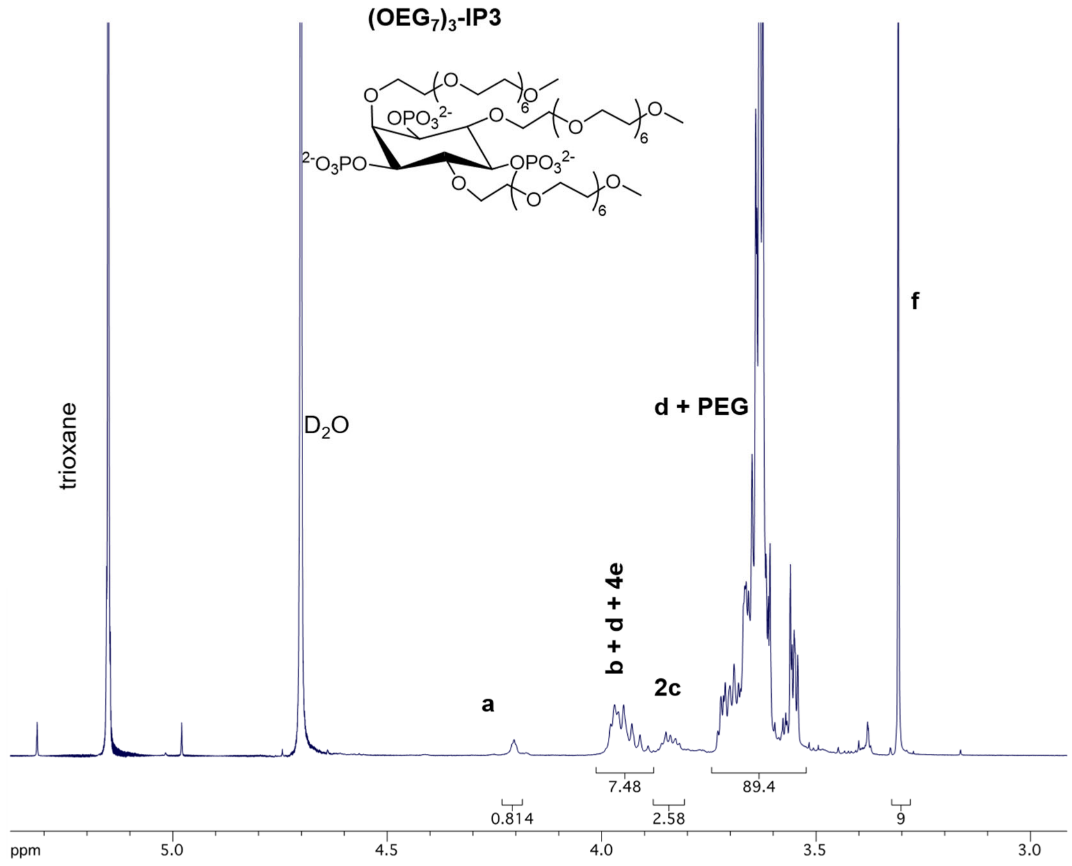

^{1}H NMR spectrum of $(OEG_7)_3$-IP3

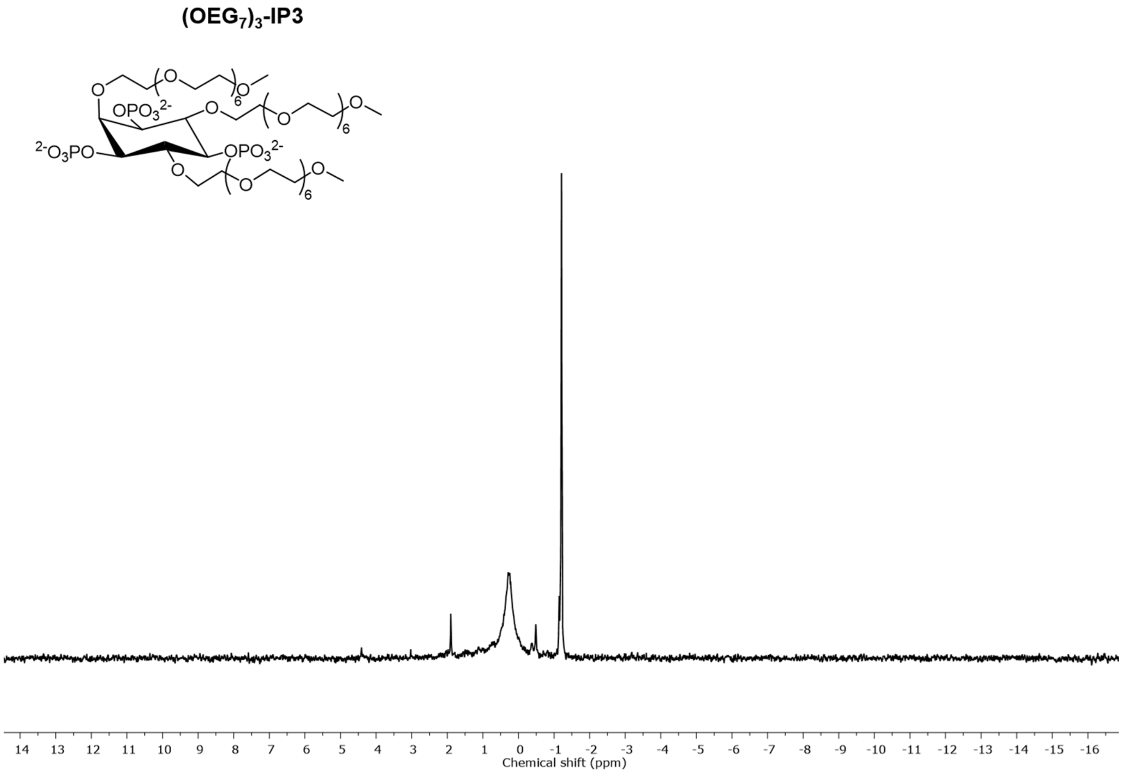

^{31}P NMR spectrum of $(OEG_7)_3$-IP3

5. Appendix ESI-MS Spectra

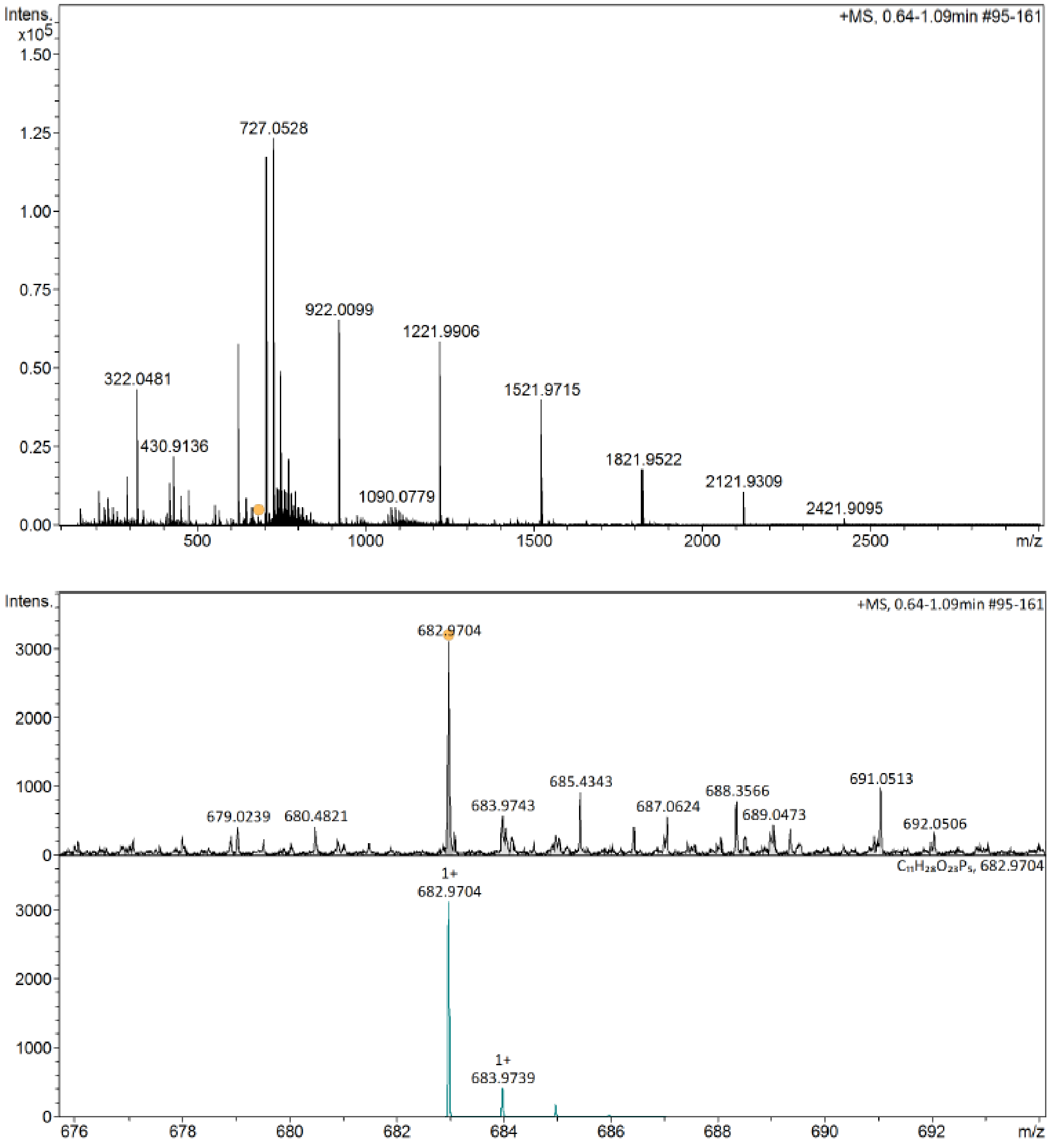

ESI-MS Spectrum of OEG$_2$-IP5

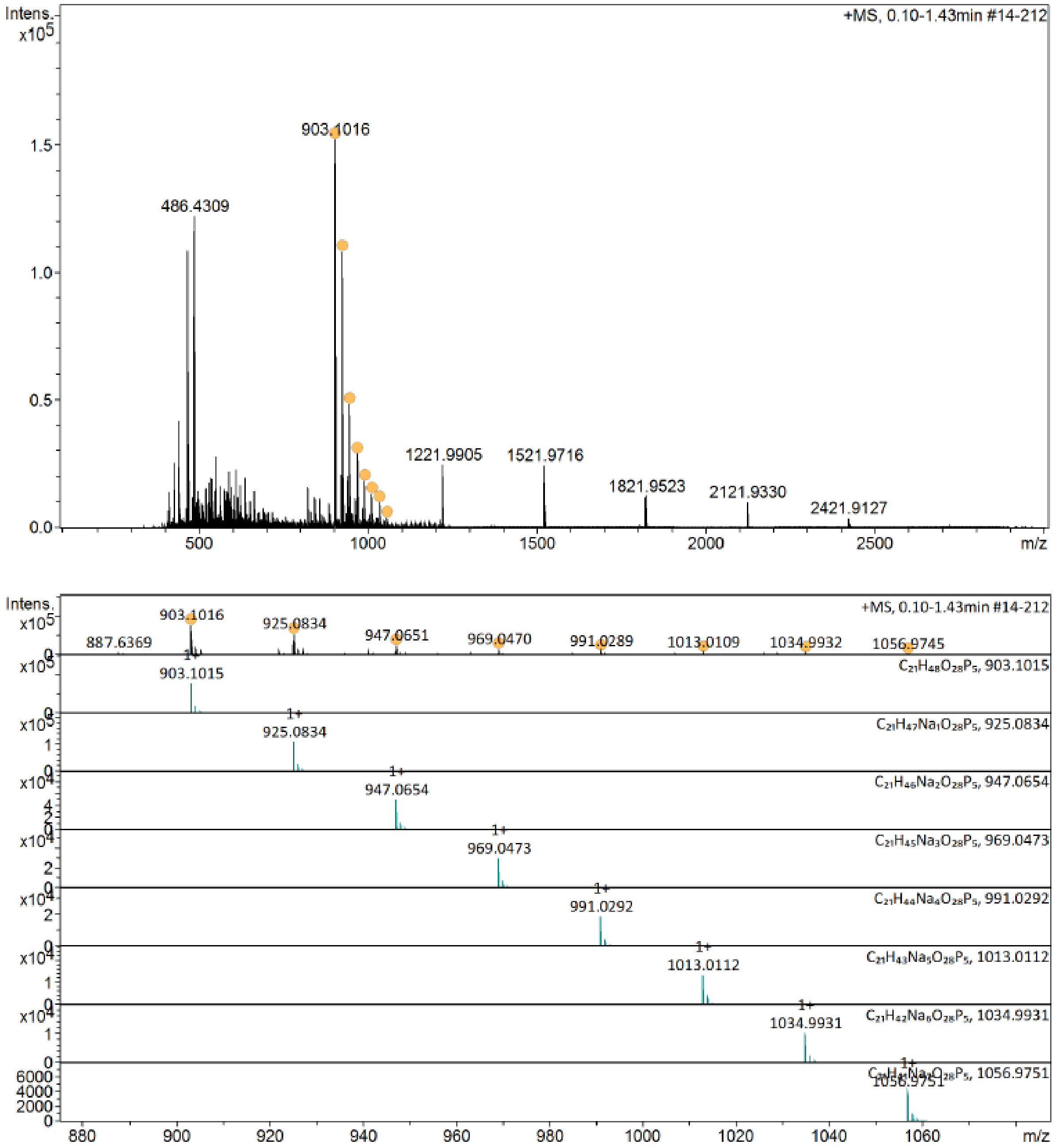

ESI-MS Spectrum of OEG$_7$-IP5

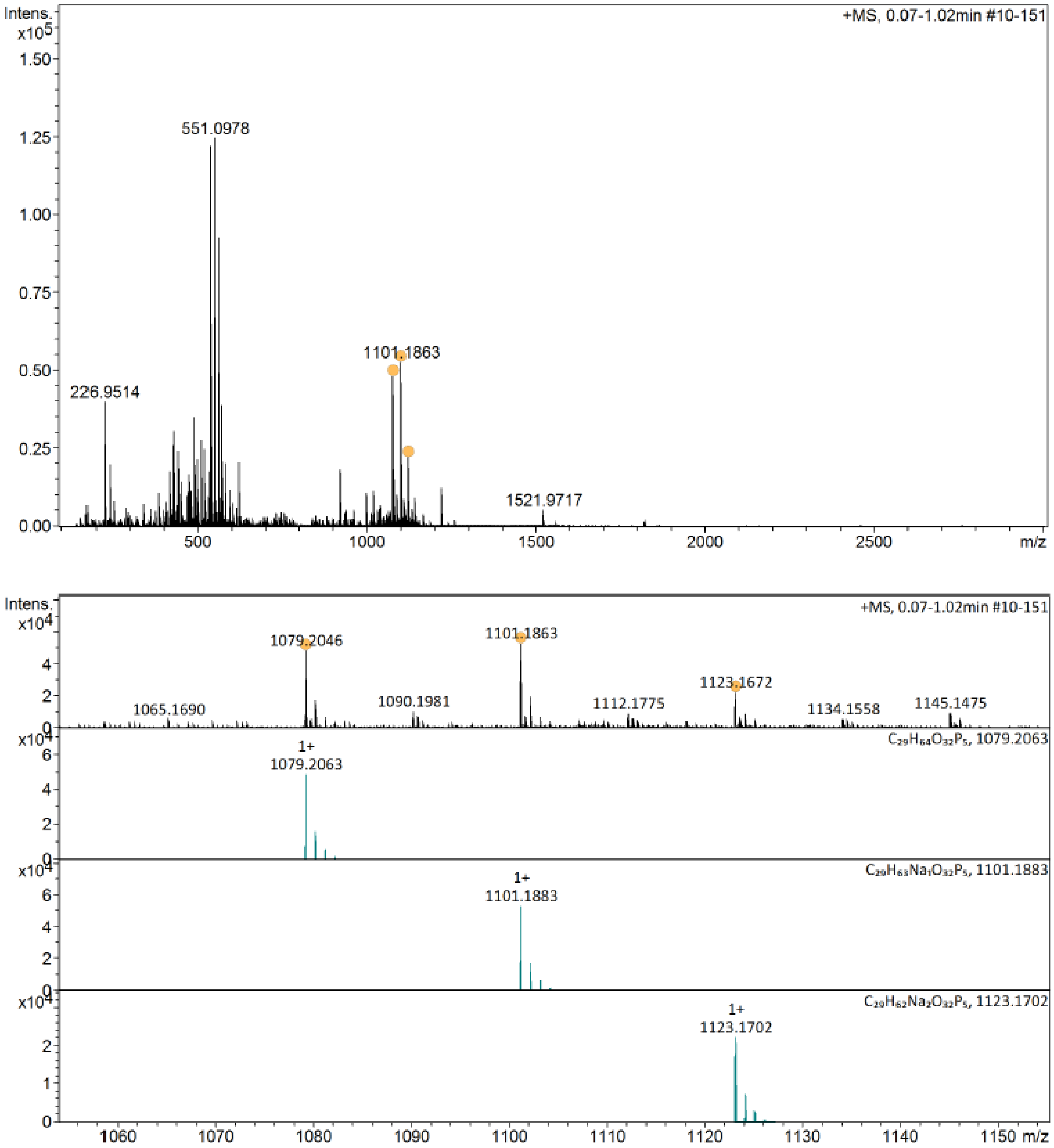

ESI-MS Spectrum of OEG$_{11}$-IP5

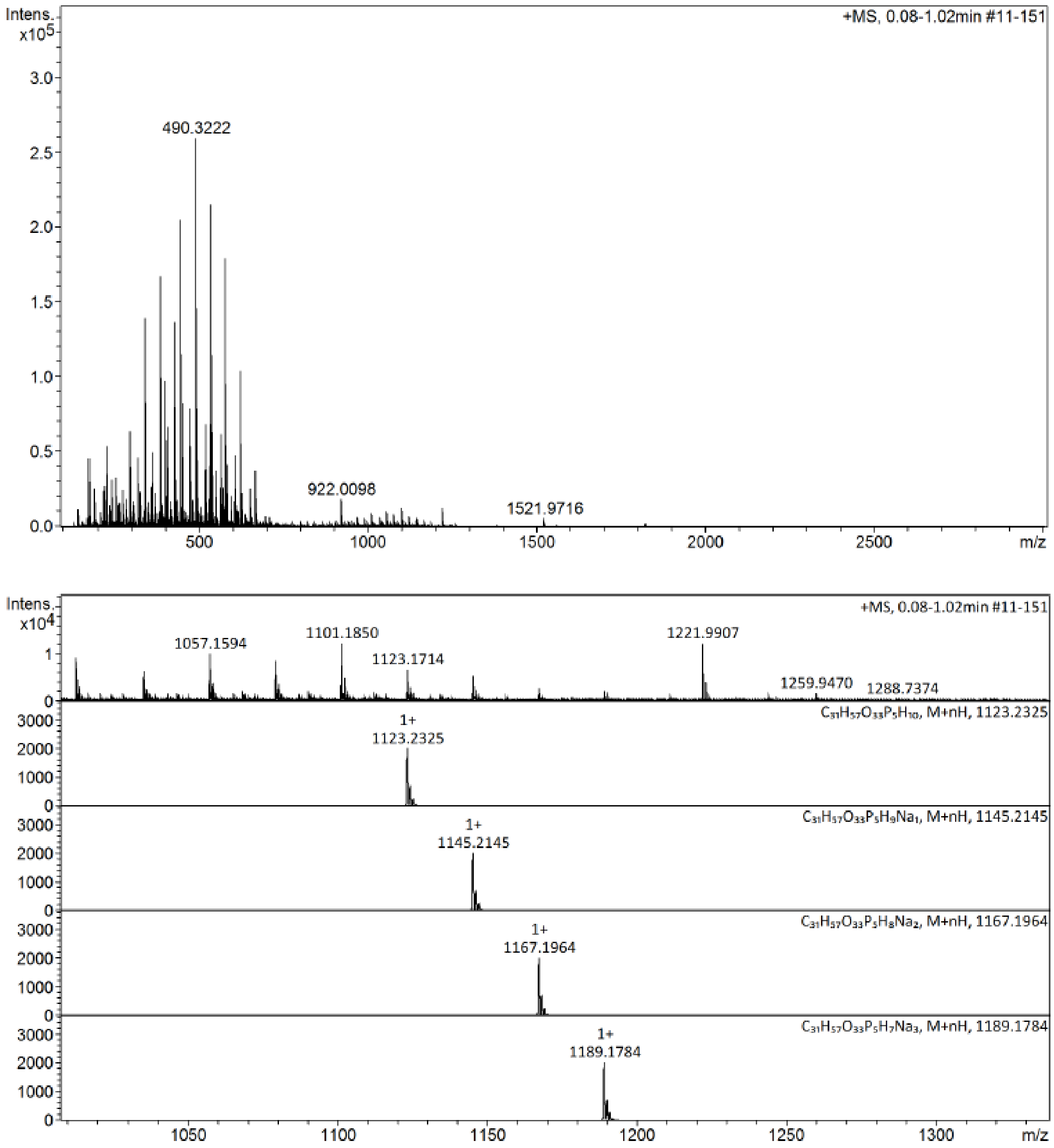

ESI-MS Spectrum of OEG_{12}-IP5

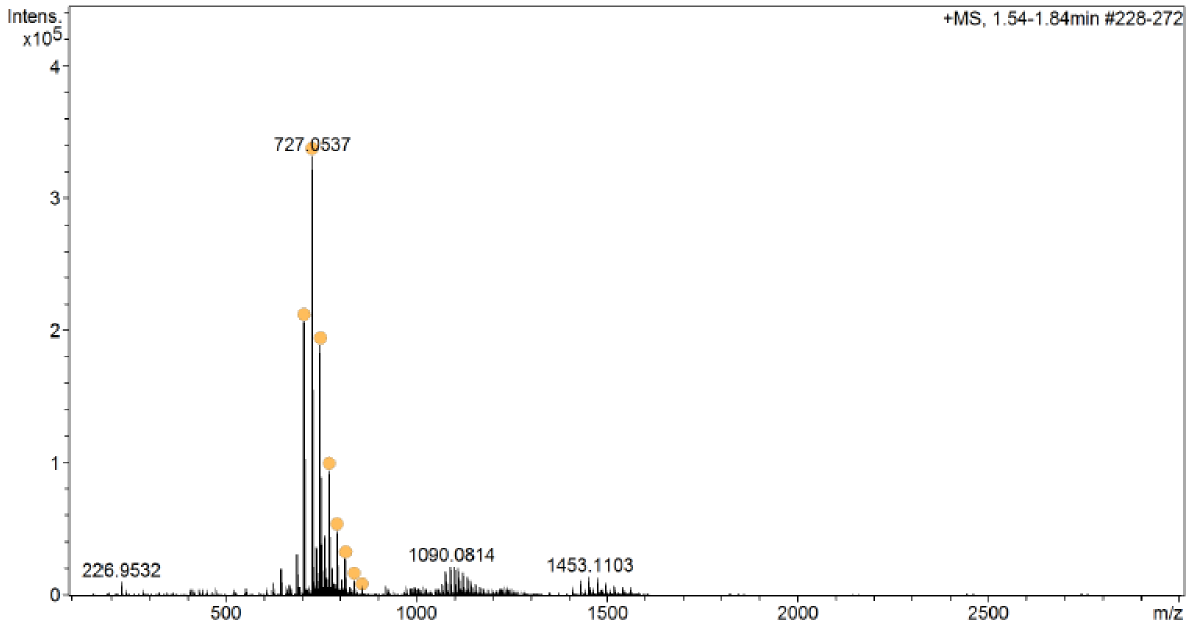

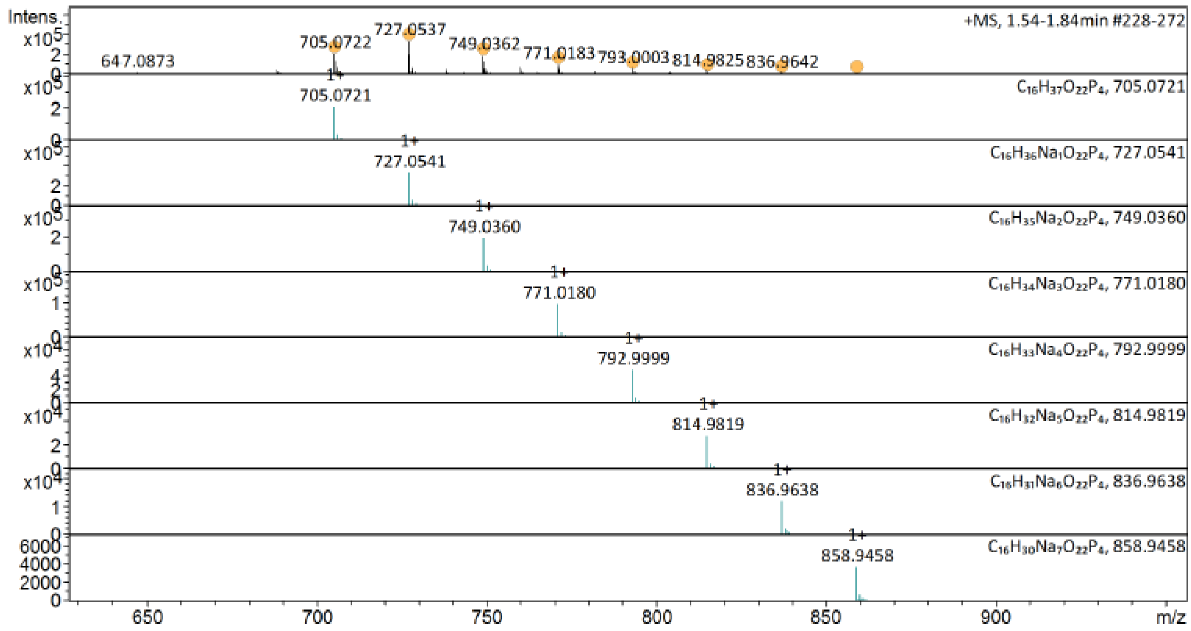

ESI-MS Spectrum of $(OEG_2)_2$-IP4

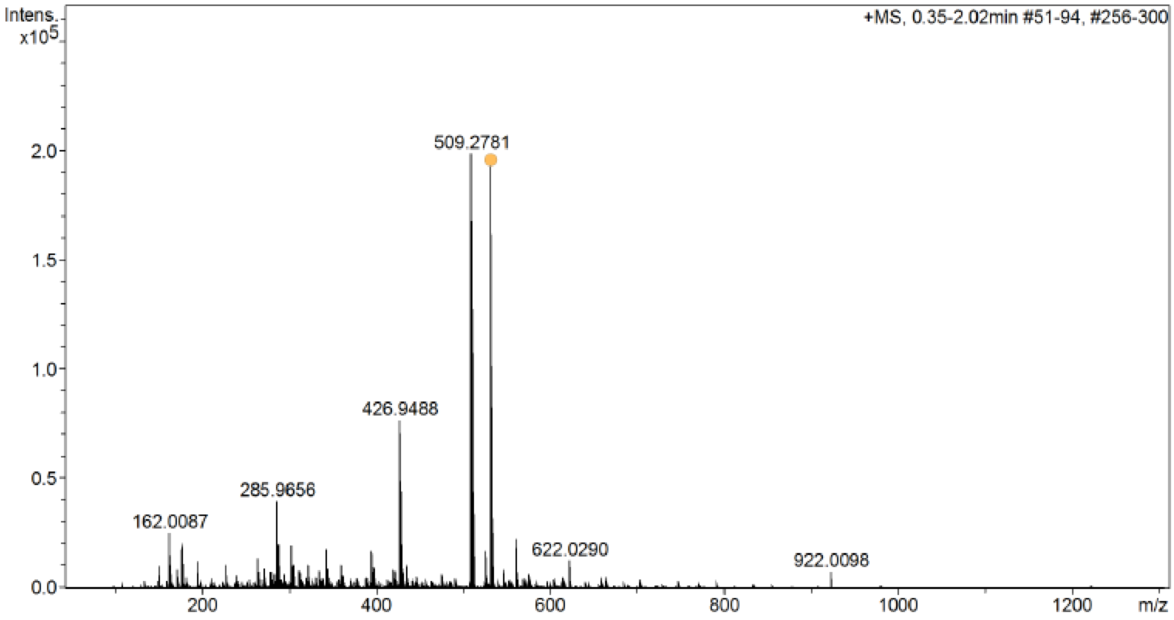

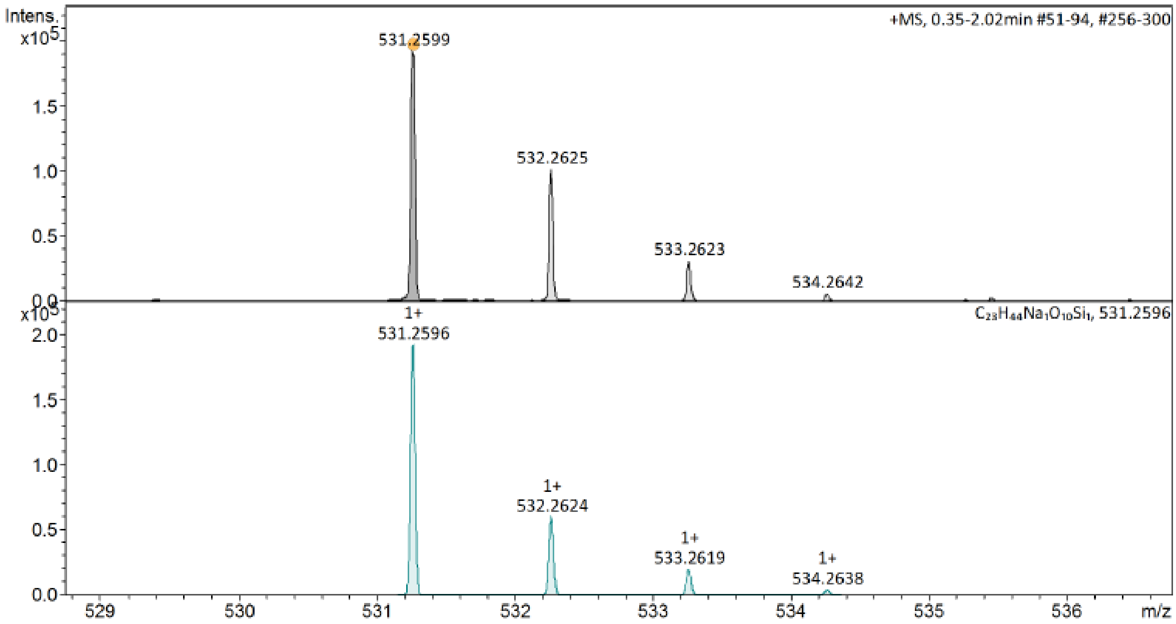

ESI-MS Spectrum of Cp5 (intermediate of $(OEG_2)_2$-IP4 synthesis)

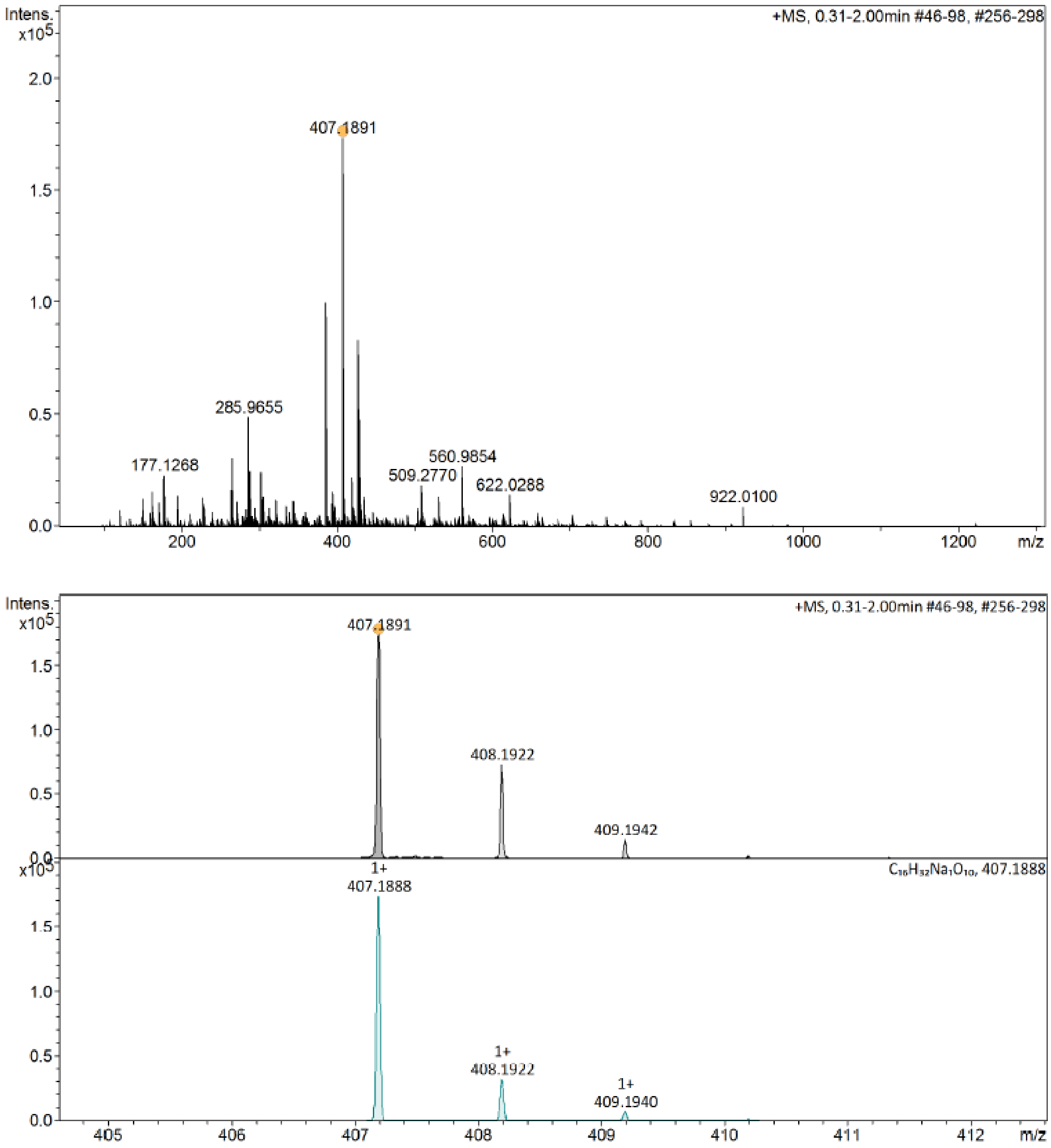

ESI-MS Spectrum of Cp6 (intermediate of $(OEG_2)_2$-IP4 synthesis)

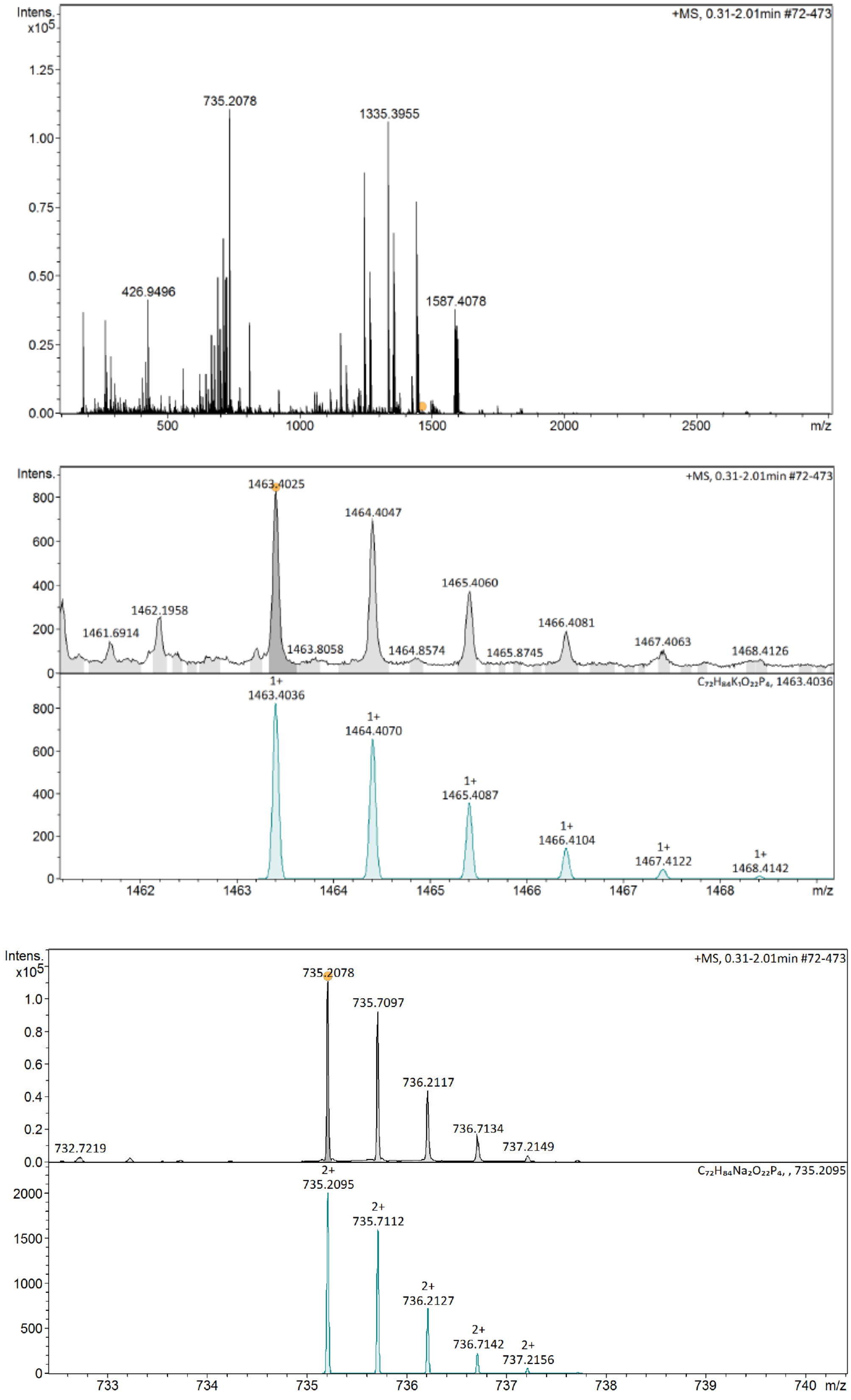

ESI-MS Spectrum of Cp7 (intermediate of $(OEG_2)_2$-IP4 synthesis)

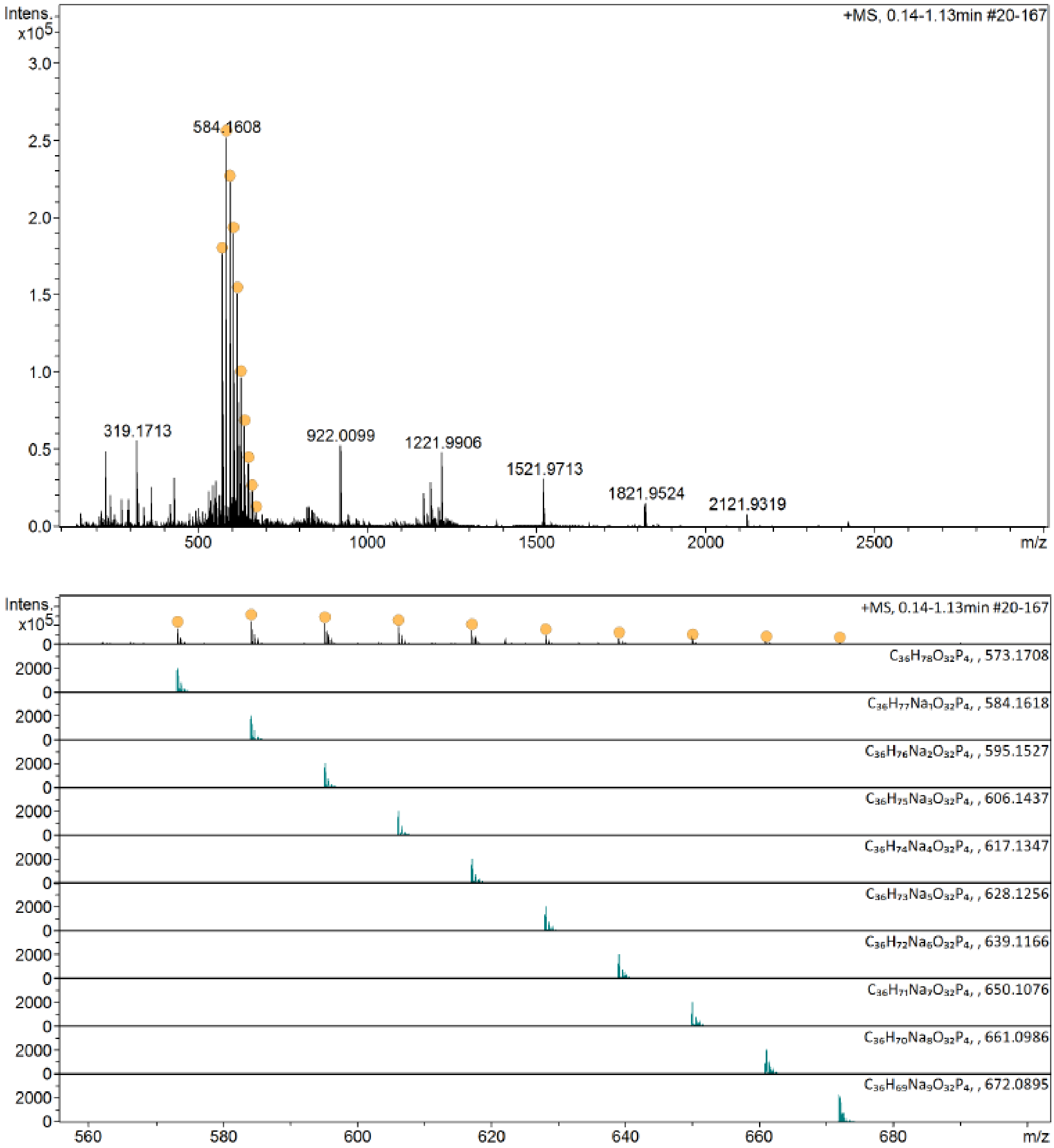

ESI-MS Spectrum of $(OEG_7)_2$-IP4

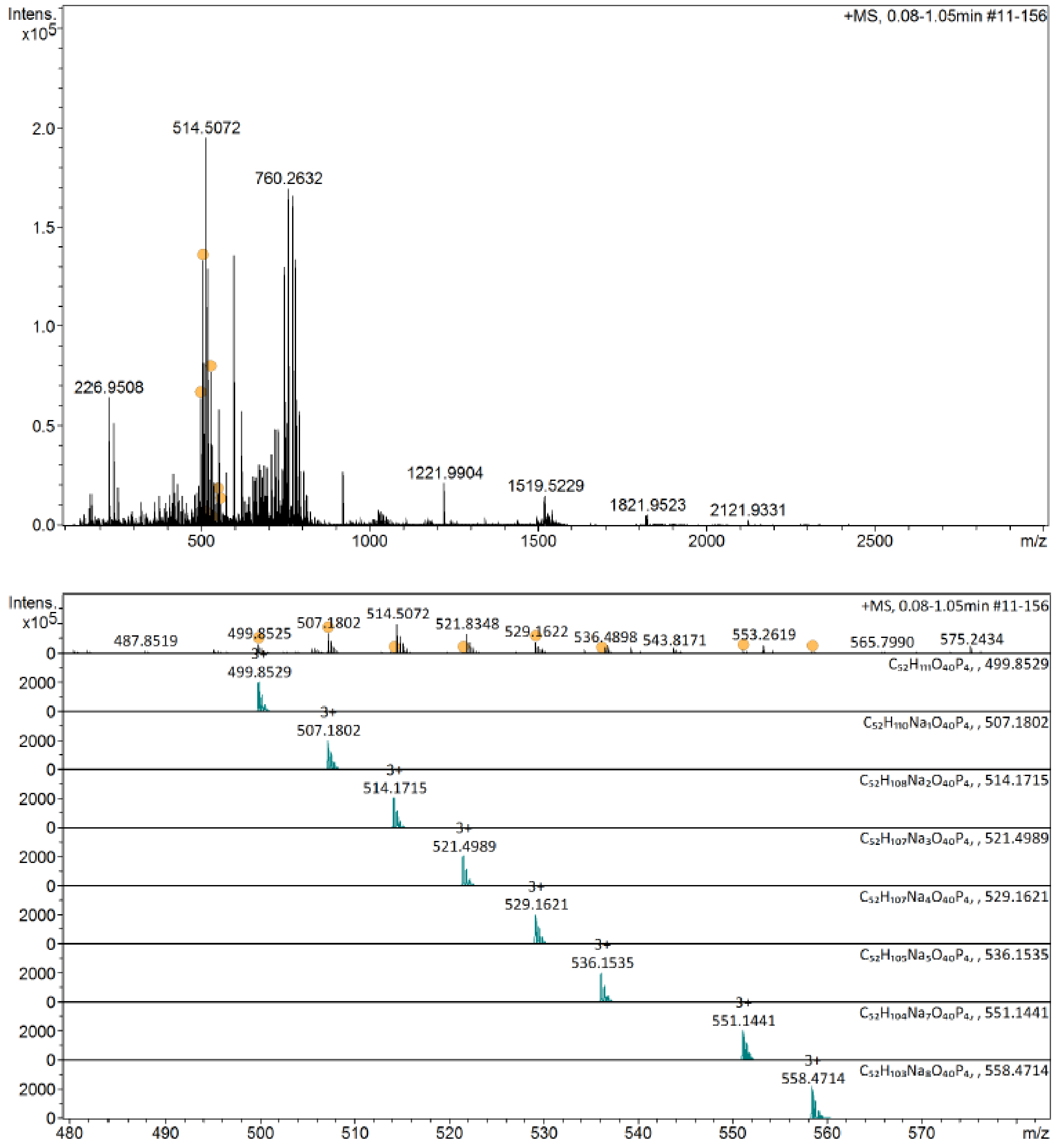

ESI-MS Spectrum of $(OEG_{11})_2$-IP4

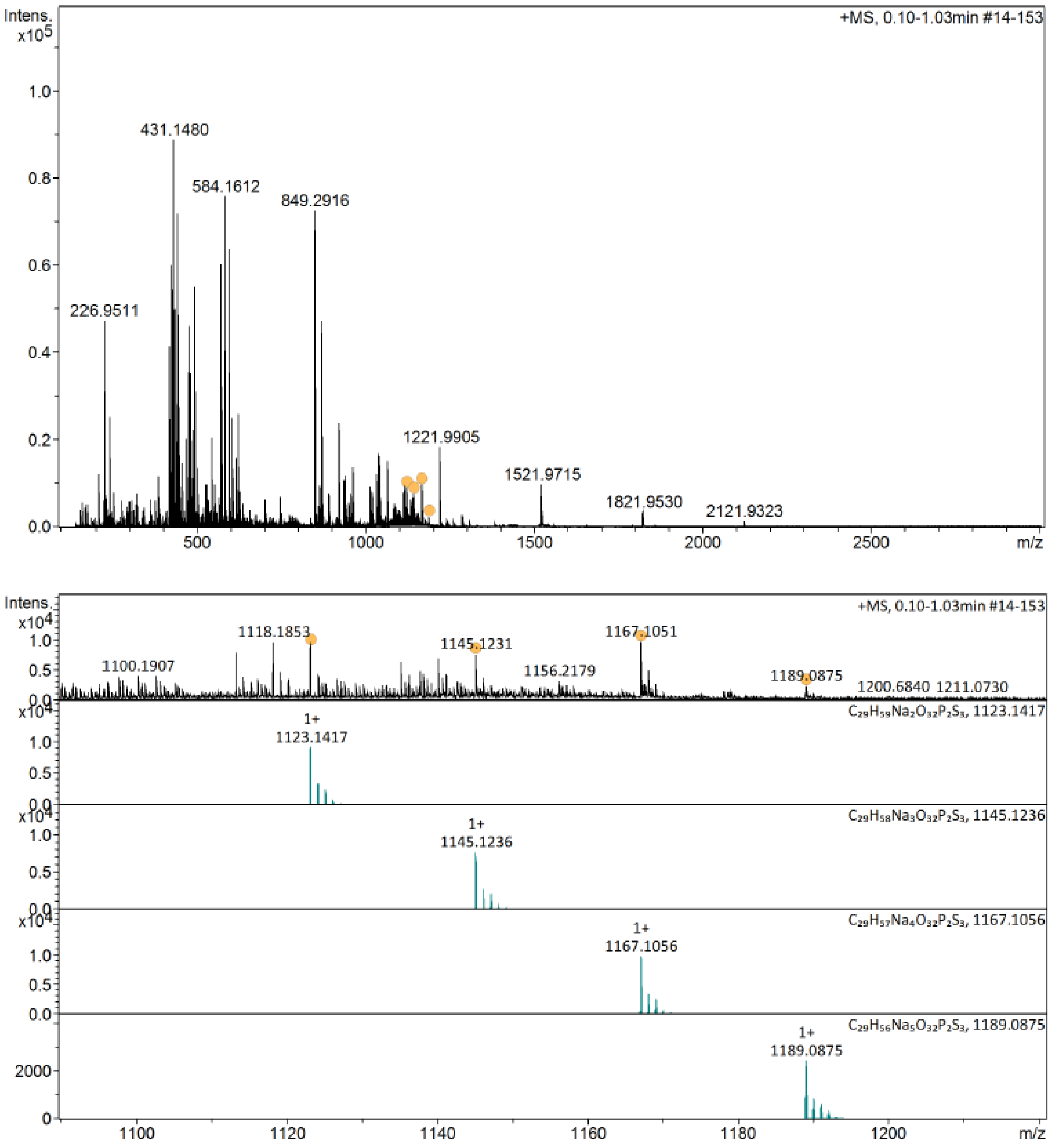

ESI-MS Spectrum of OEG$_{11}$-IP2S3

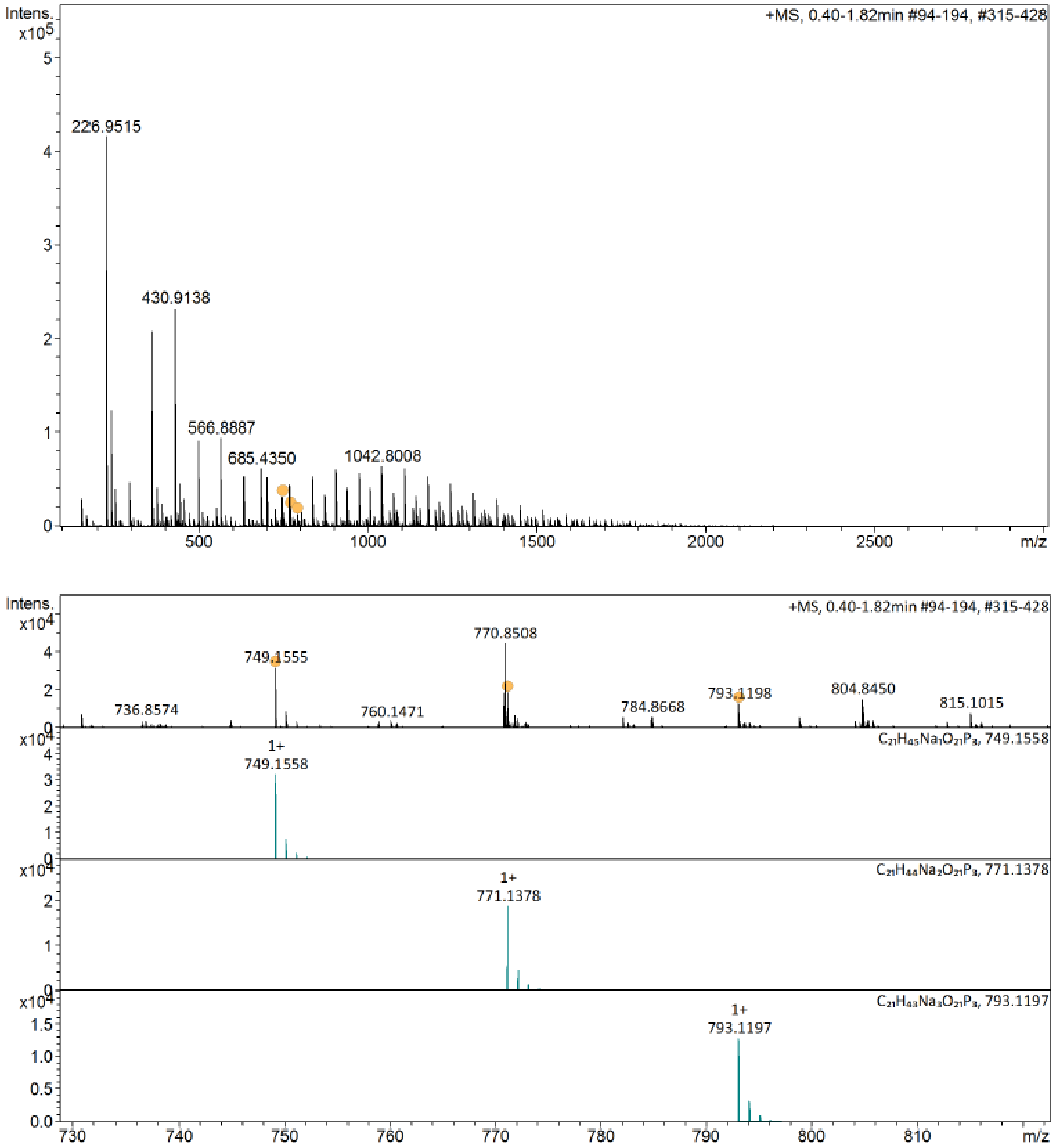

ESI-MS Spectrum of $(OEG_2)_3$-IP3

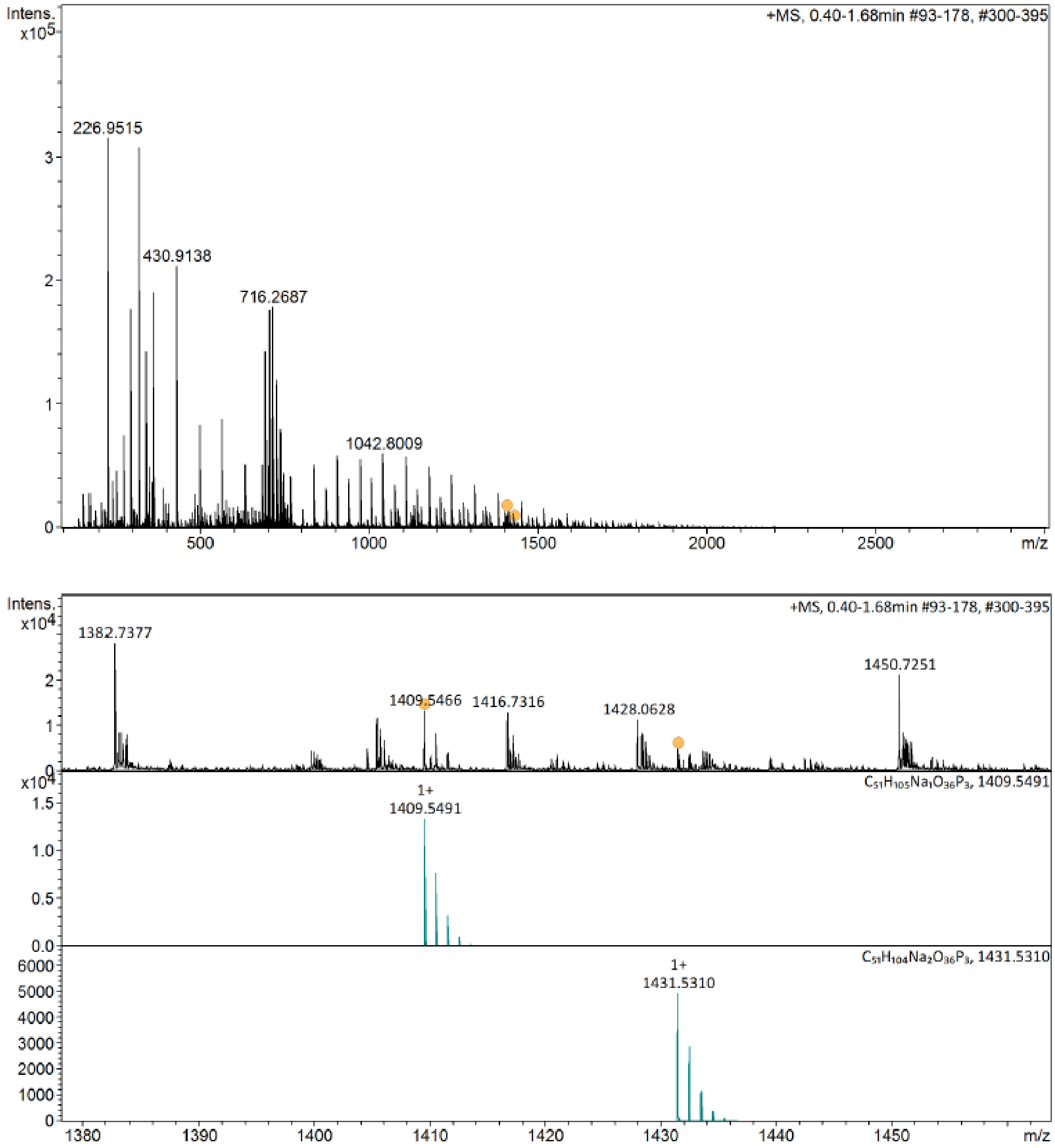

ESI-MS Spectrum of $(OEG_7)_3$-IP3

6. Appendix HPLC MS/MS Spectrum

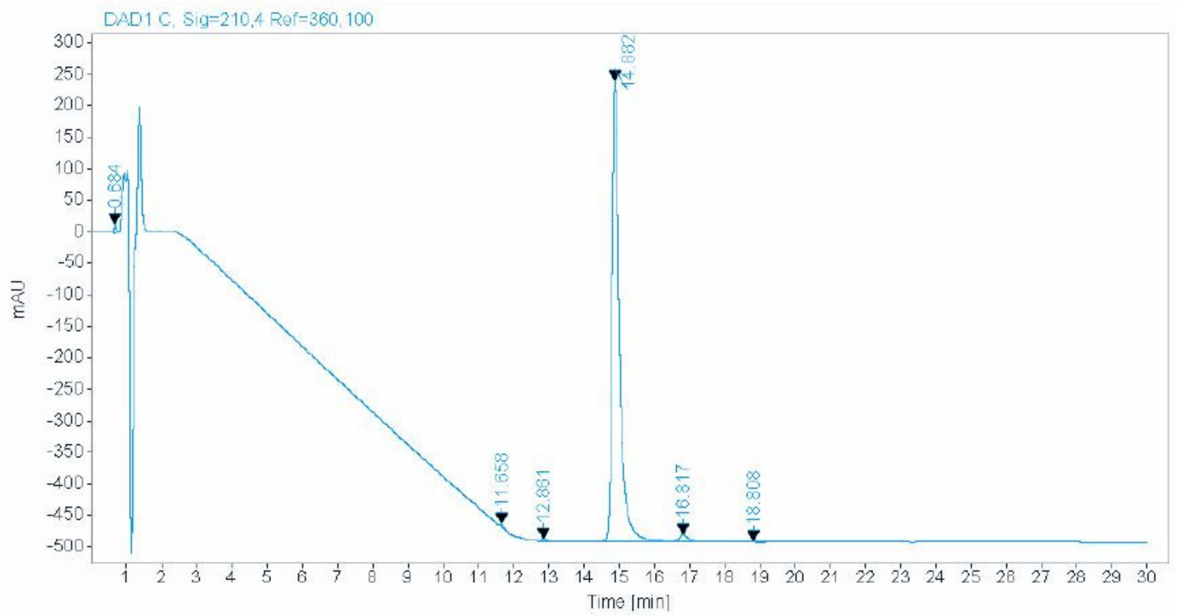

Signal: DAD1 C, Sig=210,4 Ref=360,100

Name	Peak Relative Ret Time	RT [min]	RF	Peak Area Percent	Area
	0.046	0.684		0.49	54.13186
	0.783	11.658		0.29	32.04406
	0.864	12.861		0.25	27.83403
	1.000	14.882		97.18	10716.88379
	1.130	16.817		1.56	171.62502
	1.264	18.808		0.23	25.76692

Appendix Fig. 1.: Representative HPLC chromatogram as well as peak retention times of the benzylated precursor of $(OEG_2)_2$-IP4 are presented. Purity of all synthesized compounds was confirmed by HPLC-UV analysis of the corresponding benzylated precursor, due to the fact that the synthesized inositol phosphates lack a good chromophore for UV detection whereas the benzylated precursors can absorb light.

7. Supplemental References

1. Suvorova, E. & Buffat, P. Electron diffraction and high resolution transmission electron microscopy in the characterization of calcium phosphate precipitation from aqueous solutions under biomineralization conditions. *Eur. Cells Mater.* **41**, 27–42 (2001).
2. Ivarsson, M. E. *et al.* Small-molecule allosteric triggers of clostridium difficile toxin B auto-proteolysis as a therapeutic strategy. *Cell Chem. Biol.* **26**, 17–26 (2019).
3. Godage, H. Y. *et al.* Regioselective opening of myo-inositol orthoesters: Mechanism and synthetic utility. *J. Org. Chem.* **78**, 2275–2288 (2013).
4. Fayet, A. *et al.* Determination of unbound antiretroviral drug concentrations by a modified ultrafiltration method reveals high variability in the free fraction. *Ther. Drug Monit.* **30**, 511–522 (2008).
5. Neven, E. *et al.* Disturbances in bone largely predict aortic calcification in an alternative rat model developed to study both aascular and bone pathology in chronic kidney disease. *J. Bone Miner. Res.* **30**, 2313–2324 (2015).
